# Supplementary material for: General Construction of Amine via Reduction of N=X (X = C, O, H) Bonds Mediated by Supported Nickel Boride Nanoclusters
Source: Int J Mol Sci. 2022 Aug 19;23(16):9337. doi: 10.3390/ijms23169337 (PMC9408822; doi:10.3390/ijms23169337)
Supplement: Supplementary file 1 [file ijms-23-09337-s001.zip › ijms-1846182-supplementary.pdf]

General Construction of Amine via Reduction of N=X (X = C, O, H) Bonds Mediated by Supported Nickel Boride Nanoclusters

Da Ke,[1,2] and Shaodong Zhou [1,2,*]

[1]Zhejiang Provincial Key Laboratory of Advanced Chemical Engineering Manufacture Technology, College of Chemical and Biological Engineering, Zhejiang University, Hangzhou 310027, China

[2]Institute of Zhejiang University—Quzhou, Zheda Rd. #99, Quzhou 324000, China

* Correspondence: szhou@zju.edu.cn

General information

All the chemical compounds were purchased from the following supplier: Aladdin (Shanghai, China), Macklin(Shanghai, China), Sinopharm(Shanghai, China), Meryer(Shanghai, China). All the chemicals used were of analytical grade without any further purification. ^{1}H NMR and ^{13}C NMR spectrum data were recorded using Bruker DRX-400 instrument using $CDCl_3$ or DMSO-d_6 as solvent at 298K. Gas chromatography (GC) was performed on a Agilent chromatography with a SE54 column. ICP data was obtained from Agilent-ICPOES730.The X-ray diffraction (XRD) patterns were measured at room temperature using D/max-rA with Cu-Kα radiation generated at 10mA and 40kV. The X-ray photoelectron spectroscopy (XPS) analysis was carried out by using Thermo Scientific K-Alpha with Al-Kα radiation. Transmission electron microscope (TEM) was conducted using Talos F200S with X-ray spectroscopy (EDS).

General procedure for catalyst

A typical process for catalyst preparation: 0.50 g $NiCl \cdot 6H_2O$ (2.1 mmol) was dissolved in 50 mL deionized water, adding 2.00 g TS-1 molecular sieve (XFNANO, BET:350-450 m^2/g, pore diameter:0.56~0.58 nm), stirring for 0.5 h at 30°C. After that, 40mL 1M $NaBH_4$ solution was added to the suspension keeping stirring and temperature constant for 2 h. Finally, the suspension was filtered and washed to obtain catalyst solid, the catalyst was vacuum dried at 50°C for 2 h. This catalyst was named $Ni_{6.2}$-30, “6.2” and “30” represented the mass content of nickel and the temperature of catalyst preparation, respectively. The catalysts are listed in the

Table S1.

Table S1 The parameters of different catalysts

Name	$NiCl\cdot 6H_2O$/g	TS-1/g	Temperature/°C	$NaBH_4$ solution/mL
$Ni_{6.2}$-30	0.50	2.00	30	40
$Ni_{6.2}$-50	0.50	2.00	50	40
$Ni_{6.2}$-70	0.50	2.00	70	40
$Ni_{6.2}$-100	0.50	2.00	100	40
$Ni_{2.5}$-30	0.20	2.00	30	16
$Ni_{12.4}$-30	1.00	2.00	30	80
$Ni_{18.6}$-30	1.50	2.00	30	120
$Ni_{24.8}$-30	2.00	2.00	30	160

The activity of catalyst for reduction

Nitrile compound: nitrile (5 mmol), catalyst (50 mg) and isopropyl alcohol (20 mL) were mixed in a 100 mL volume autoclave (Labe instrument, Shanghai) equipped with PTFE and magnetic pellet. The kettle is filled with 0.5 MPa ammonia gas and heated to 120°C, at this temperature, 4.0 MPa H_2 was pressed in, then reaction was started. During the process, the system pressure was controlled between 4.0±0.1 MPa. After reaction (the pressure was not changing), the autoclave was cooled and degassed, the reaction solution was filtered to recover the catalyst, and the filtrate was concentrated to determine the conversion by GC. The crude product was purified by column chromatography using silica gel. It should be noted that some products can be detected by NMR without further purification.

Nitro compound: the operation process was the same as the above, the difference is the reaction temperature, the temperature of this group of experiments was 120°C. Of course, this

set of experiments did not require an atmosphere of ammonia.

Aldehyde and ammonia reaction: coincidentally, this set of experiments was consistent with the reaction operation of nitrile compounds, however, in this group of experiments, the main role of ammonia was to participate in the reaction as a reactant, not just to inhibit side reactions. The reaction temperature was 120°C.

Supplementary result

Solvent selection

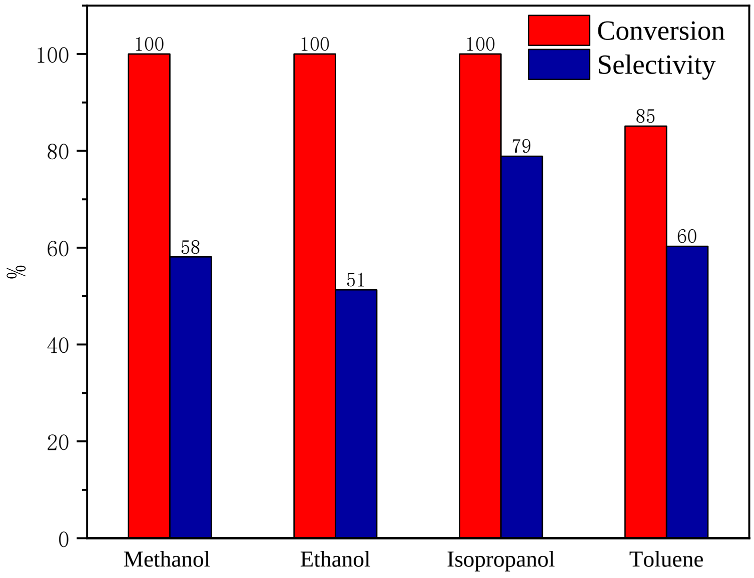

Figure S1 Solvent selection result, reaction condition: 5.0 mmol benzonitrile, 50 mg $Ni_{12.4}$-30 catalyst (about 2.0 mol% Ni), 0.5 MPa NH_3 and 4.0 MPa H_2, 20 mL solvent, 120°C, 3h, conversion and selectivity were calculated by GC.

Temperature optimization

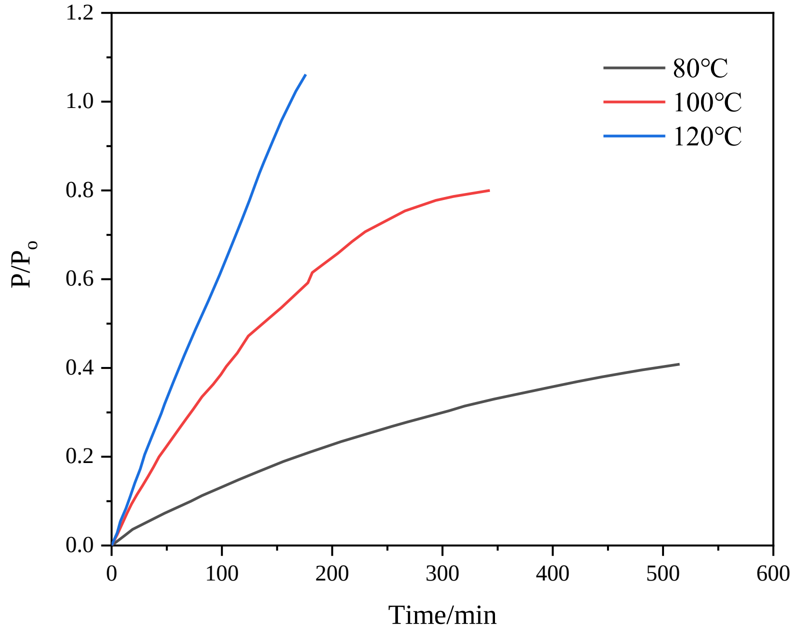

Figure S2 Temperature optimization result, reaction condition: 25.0 mmol benzonitrile, 250 mg $Ni_{12.4}$-30 catalyst (about 2.0 mol% Ni), 4.0 MPa H_2, 20 mL isopropanol. P is cumulative hydrogen absorption, P_0 is theoretical hydrogen absorption

Pressure optimization

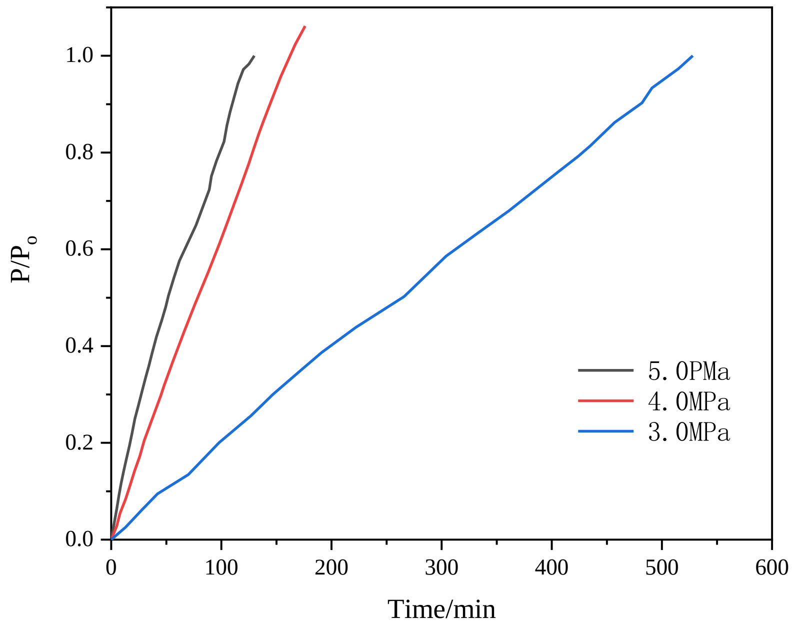

Figure S3 Pressure optimization result, reaction condition: 25.0 mmol benzonitrile, 250 mg $Ni_{12.4}$-30 catalyst (about 2.0 mol% Ni), 120°C, 20 mL of isopropanol. P is cumulative hydrogen absorption, P_0 is theoretical hydrogen absorption

ICP-OES

Table S2 The data of ICP-OES

	Mass m_0（g)	Volume V_0（mL)	Element	Concentration C_o（mg/L)	Dilution factor f	Original concentration C_1（mg/L)	Element content C_x（mg/kg)	Weight content W（%)
Ni(6.2)-100	0.0524	25	Ni	2.85	50	142.74	68100.43	6.81%
Ni(6.2)-70	0.0488	25	Ni	2.26	50	112.82	57798.16	5.78%
Ni(6.2)-50	0.0711	25	Ni	3.15	50	157.66	55435.30	5.54%
Ni(6.2)-30	0.0534	25	Ni	2.41	50	120.47	56399.58	5.64%
Ni(24.8)-30	0.0543	25	Ni	10.16	50	507.78	233782.23	23.38%
Ni(18.6)-30	0.0556	25	Ni	7.96	50	397.78	178855.67	17.89%
Ni(12.4)-30	0.0681	25	Ni	6.58	50	329.00	120778.27	12.08%
Ni(2.5)-30	0.0701	25	Ni	1.19	50	59.48	21211.84	2.12%

XRD patterns

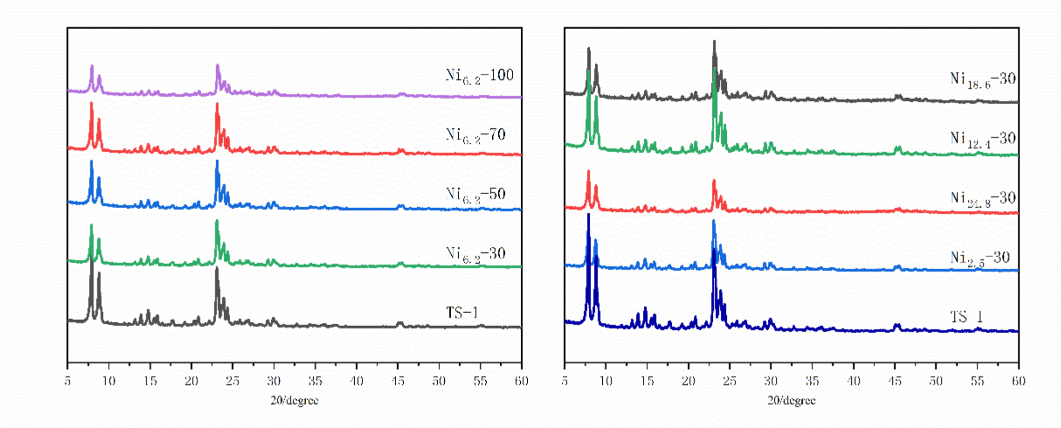

Figure S4 XRD patterns of other samples in in different prepared conditions

Comparison between $Ni_{12.4}$-30 and Raney Ni

A commercial Raney nickel catalyst (≤150μm, purchase from Aladdin) was used for performance comparison with the catalysts prepared in this work. Nickel content in the reaction

system kept constant with 2.0mol% around. The result was shown in Table S 3.

Table S3 The comparison between two catalysts

	Catalyst	Weight /mg	Time /h	Conversion/%	Yield/%
Benzonitrile	$Ni_{12.4}$-30	50	3.5	100	77
	Raney Ni	10	4	100	26
Nitrobenzene	$Ni_{12.4}$-30	50	5	100	97
	Raney Ni	10	2	100	97
Benzaldehyde	$Ni_{12.4}$-30	50	2	100	96
	Raney Ni	10	3.5	100	92

Condition: Benzonitrile 5.0 mmol, 120°C, 4.0 MPa H_2, 20 mL of isopropanol. Nitrobenzene 5.0 mmol, 120°C, 4.0 MPa H_2, 20 mL of isopropanol. Benzaldehyde 5.0 mmol,120°C, 0.5 MPa NH_3 and 4.0 MPa H_2, 20 mL of isopropanol.

Characteristic data for all products

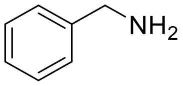

^{1}H NMR (400 MHz, Chloroform-*d*) δ 7.35 – 7.18 (m, 5H), 3.82 (s, 2H), 1.46 (brs, 2H).
^{13}C NMR (101 MHz, Chloroform-*d*) δ 143.40, 128.53, 127.07, 126.77, 46.52.

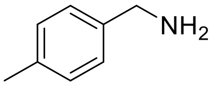

^{1}H NMR (400 MHz, Chloroform-*d*) δ 7.20 – 7.06 (m, 4H), 3.78 (s, 2H), 2.31 (s, 3H), 1.41 (brs, 2H).
^{13}C NMR (101 MHz, Chloroform-*d*) δ 140.50, 136.26, 129.20, 127.04, 46.27, 21.05.

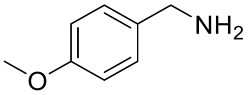

^{1}H NMR (400 MHz, DMSO-d_6) δ 7.27 (d, J = 8.3 Hz, 2H), 6.88 (d, J = 8.6 Hz, 2H), 3.74 (s, 3H), 3.71 (s, 2H), 3.01 (brs, 2H).
^{13}C NMR (101 MHz, DMSO-d_6) δ 157.89, 135.91, 128.04, 113.35, 54.72, 45.05.

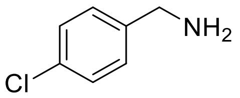

^{1}H NMR (400 MHz, DMSO-d_6) δ 7.36 (d, *J* = 9.2 Hz, 2H), 7.33 (d, *J* = 8.4 Hz, 2H), 3.71 (s, 2H), 2.01 (brs, 2H).

^{13}C NMR (101 MHz, DMSO-d_6) δ 143.22, 130.63, 128.74, 127.88, 44.92.

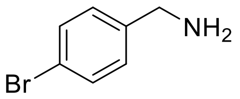

^{1}H NMR (400 MHz, Chloroform-*d*) δ 7.42 (d, *J* = 8.4 Hz, 2H), 7.16 (d, *J* = 8.4 Hz, 2H), 3.79 (brs, 2H), 1.55 (s, 2H).

^{13}C NMR (101 MHz, Chloroform-*d*) δ 142.16, 131.50, 128.83, 120.44, 45.77.

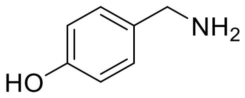

^{1}H NMR (400 MHz, Chloroform-*d*) δ 7.16 (m, 2H), 6.76 (m, 2H), 3.80 (s, 2H).

^{13}C NMR (101 MHz, Chloroform-*d*) δ 128.57, 115.52, 45.82.

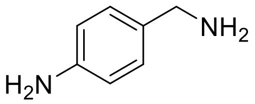

^{1}H NMR (400 MHz, Chloroform-*d*) δ 7.08 (d, *J* = 8.4 Hz, 2H), 6.64 (d, *J* = 8.4 Hz, 2H), 3.73 (s, 2H).

^{13}C NMR (101 MHz, Chloroform-*d*) δ 145.23, 133.57, 128.22, 115.23, 46.04.

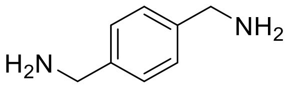

^{1}H NMR (400 MHz, DMSO-d_6) δ 7.28 (s, 4H), 3.73 (s, 4H), 2.01 (brs, 4H).

^{13}C NMR (101 MHz, DMSO-d_6) δ 142.09, 126.78, 45.53.

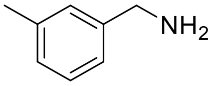

^{1}H NMR (400 MHz, Chloroform-*d*) δ 7.20 (t, *J* = 7.5 Hz, 1H), 7.13 – 6.97 (m, 3H), 3.79 (s, 2H), 2.33 (s, 3H), 1.45 (brs, 2H).

^{13}C NMR (101 MHz, Chloroform-*d*) δ 143.39, 138.13, 128.46, 127.87, 127.51, 124.11, 46.52, 21.40.

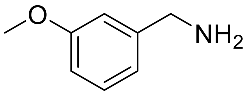

^{1}H NMR (400 MHz, DMSO-d_6) δ 7.27 (t, *J* = 7.8 Hz, 1H), 7.07 – 6.79 (m, 3H), 3.80 (s, 2H), 3.78 (s, 3H), 1.96 (brs, 2H).

^{13}C NMR (101 MHz, DMSO-d_6) δ 159.41, 145.98, 129.01, 119.13, 112.44, 111.59, 54.71, 45.80.

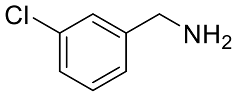

^{1}H NMR (400 MHz, DMSO-*d*6) δ 7.42 (d, *J* = 1.9 Hz, 1H), 7.38 – 7.17 (m, 3H), 3.73 (s, 2H), 2.16 (brs, 2H).

^{13}C NMR (101 MHz, DMSO-*d*6) δ 146.94, 132.92, 129.74, 126.78, 125.95, 125.53, 45.09.

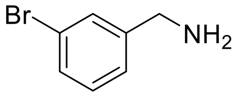

^{1}H NMR (400 MHz, Chloroform-*d*) δ 7.47 (s, 1H), 7.36 (dt, *J* = 7.5, 1.7 Hz, 1H), 7.25 – 7.14 (m, 2H), 3.83 (s, 2H), 1.50 (brs, 2H).

^{13}C NMR (101 MHz, Chloroform-*d*) δ 145.60, 130.17, 130.08, 129.83, 125.67, 122.63, 45.92.

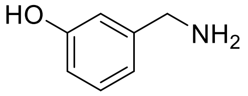

^{1}H NMR (400 MHz, DMSO-*d*6) δ 7.09 (t, *J* = 7.8 Hz, 1H), 6.87 – 6.69 (m, 2H), 6.64 (d, *J* = 8.1 Hz, 1H), 4.48 (brs, 2H), 3.65 (s, 2H).

^{13}C NMR (101 MHz, DMSO-*d*6) δ 157.54, 145.08, 128.98, 117.46, 114.07, 113.33, 45.43.

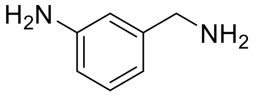

^{1}H NMR (400 MHz, Chloroform-*d*) δ 7.08 (t, *J* = 7.7, 1.6 Hz, 1H), 6.79 – 6.34 (m, 3H), 3.71 (s, 2H).

^{13}C NMR (101 MHz, Chloroform-*d*) δ 146.87, 144.53, 129.45, 117.11, 113.75, 113.58, 46.39.

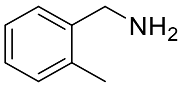

^{1}H NMR (400 MHz, Chloroform-*d*) δ 7.24 (d, *J* = 7.1 Hz, 1H), 7.18 – 7.03 (m, 3H), 3.76 (m, 2H), 2.27 (s, 3H), 1.34 (brs, 2H).

^{13}C NMR (101 MHz, Chloroform-*d*) δ 141.22, 135.46, 130.26, 127.03, 126.78, 126.19, 44.12, 18.80.

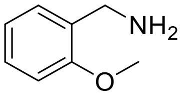

^{1}H NMR (400 MHz, DMSO-*d*6) δ 7.43 – 7.14 (m, 2H), 7.03 – 6.84 (m, 2H), 3.78 (s, 3H), 3.71 (s, 2H), 2.39 (brs, 2H).

^{13}C NMR (101 MHz, DMSO-*d*6) δ 156.70, 131.88, 127.69, 127.35, 120.08, 110.10, 55.00, 40.85.

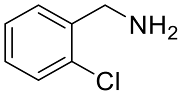

^{1}H NMR (400 MHz, DMSO-*d*6) δ 7.57 (d, *J* = 7.3 Hz, 1H), 7.38 (d, *J* = 7.8 Hz, 1H), 7.32 (t, *J* = 7.4 Hz, 1H), 7.24 (t, *J* = 7.6 Hz, 1H), 3.80 (d, *J* = 2.2 Hz, 2H), 1.90 (brs, 2H).

^{13}C NMR (101 MHz, DMSO-d_6) δ 141.12, 132.01, 128.78, 128.74, 127.78 , 126.99 , 46.23.

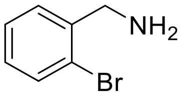

^{1}H NMR (400 MHz, Chloroform-*d*) δ 7.53 (d, *J* = 7.8 Hz, 1H), 7.36 (d, *J* = 5.8 Hz, 1H), 7.28 (t, *J* = 6.9, 6.5 Hz, 1H), 7.10 (t, *J* = 6.7, 3.3 Hz, 1H), 3.90 (s, 2H), 1.61 (brs, 2H).

^{13}C NMR (101 MHz, Chloroform-*d*) δ 142.25, 132.83, 129.07, 128.45, 127.73, 123.53, 46.98.

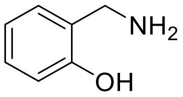

^{1}H NMR (400 MHz, Chloroform-*d*) δ 7.16 (t, *J* = 7.5 Hz, 1H), 6.96 (d, *J* = 7.4, 1.7 Hz, 1H), 6.85 (d, *J* = 8.2 Hz, 1H), 6.77 (t, *J* = 7.4 Hz, 1H), 4.14 (s, 2H).

^{13}C NMR (101 MHz, Chloroform-*d*) δ 158.40, 128.71, 127.94, 119.04, 116.80, 45.35.

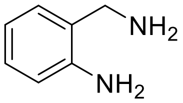

^{1}H NMR (400 MHz, Chloroform-*d*) δ 7.13 – 6.99 (m, 2H), 6.78 – 6.62 (m, 2H), 4.51 (brs, 2H), 3.88 (s, 2H), 1.28 (brs, 2H).

^{13}C NMR (101 MHz, Chloroform-*d*) δ 146.37, 129.05, 128.25, 126.22, 117.96, 115.86, 45.06.

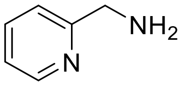

^{1}H NMR (400 MHz, DMSO-d_6) δ 8.50 (d, *J* = 3.9 Hz, 1H), 7.75 – 7.64 (m, 1H), 7.43 (d, *J* = 7.7 Hz, 1H), 7.22 – 7.07 (m, 1H), 3.87 (s, 2H), 2.28 (brs, 2H).

^{13}C NMR (101 MHz, DMSO-d_6) δ 162.88, 148.56, 136.28, 121.41, 120.84, 47.44.

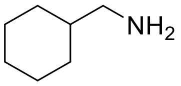

^{1}H NMR (400 MHz, DMSO-d_6) δ 2.39 (d, *J* = 6.3 Hz, 2H), 1.85 – 1.56 (m, 5H), 1.18 (m, 6H), 0.87 (m, 2H).

^{13}C NMR (101 MHz, DMSO-d_6) δ 48.49, 40.99, 30.46, 26.36, 25.69.

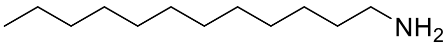

^{1}H NMR (400 MHz, DMSO-d_6) δ 2.25 (t, *J* = 6.6 Hz, 2H), 0.99 (m, 20H), 0.79 (m, 2H), 0.61 (t, *J* = 6.5 Hz, 3H).

^{13}C NMR (101 MHz, DMSO-d_6) δ 41.73, 33.53, 31.32, 29.10 (dd, *J* = 6.0, 3.5 Hz), 28.75, 26.49, 22.09, 13.81.

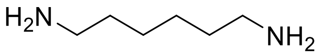

^{1}H NMR (400 MHz, Chloroform-*d*) δ 2.68 (t, *J* = 7.0 Hz, 4H), 1.48 – 1.41 (m, 4H), 1.34 (t, *J* = 7.4, 3.5 Hz, 4H).

^{13}C NMR (101 MHz, Chloroform-*d*) δ 42.14, 33.77, 26.73.

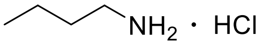

^{1}H NMR (400 MHz, DMSO-*d*$_6$) δ 8.09 (brs, 3H), 2.80 – 2.62 (m, 2H), 1.54 (p, *J* = 7.5 Hz, 2H), 1.33 (h, *J* = 7.4 Hz, 2H), 0.88 (t, *J* = 7.3 Hz, 3H).

^{13}C NMR (101 MHz, DMSO-*d*$_6$) δ 38.88, 29.45, 19.62, 13.93.

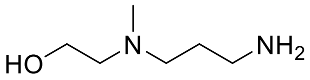

^{1}H NMR (400 MHz, DMSO-*d*$_6$) δ 3.46 (t, *J* = 6.4 Hz, 2H), 2.74 (brs, 2H), 2.53 (d, *J* = 6.9 Hz, 2H), 2.34 (dt, *J* = 14.3, 6.8 Hz, 4H), 2.28 (s, 1H), 2.14 (s, 3H), 1.47 (t, *J* = 6.9 Hz, 2H).

^{13}C NMR (101 MHz, DMSO-*d*$_6$) δ 59.66, 58.85, 55.38, 42.33, 39.84, 30.55.

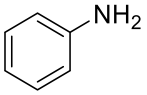

^{1}H NMR (400 MHz, Chloroform-*d*) δ 7.20 – 7.04 (m, 2H), 6.78 – 6.67 (m, 1H), 6.66 – 6.56 (m, 2H), 3.55 (brs, 2H).

^{13}C NMR (101 MHz, Chloroform-*d*) δ 146.58, 129.38, 118.58, 115.20.

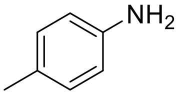

^{1}H NMR (400 MHz, Chloroform-*d*) δ 6.93 (d, *J* = 7.9 Hz, 2H), 6.55 (d, *J* = 8.5 Hz, 2H), 3.46 (brs, 2H), 2.21 (s, 3H).

^{13}C NMR (101 MHz, Chloroform-*d*) δ 144.02, 129.85, 127.75, 115.36, 20.53.

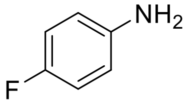

^{1}H NMR (400 MHz, Chloroform-*d*) δ 6.84 (t, *J* = 8.4 Hz, 2H), 6.67 – 6.41 (m, 2H), 3.52 (brs, 2H).

^{13}C NMR (101 MHz, Chloroform-*d*) δ 156.44 (d, *J* = 235.4 Hz), 142.49, 116.08 (d, *J* = 7.6 Hz), 115.67 (d, *J* = 22.5 Hz).

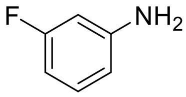

^{1}H NMR (400 MHz, Chloroform-*d*) δ 7.14 – 6.87 (m, 1H), 6.50 – 6.24 (m, 3H), 3.72 (brs, 2H).

^{13}C NMR (101 MHz, Chloroform-*d*) δ 163.94 (d, *J* = 243.0 Hz), 148.40 (d, *J* = 10.8 Hz), 130.50 (d, *J* = 10.0 Hz), 110.76 (d, *J* = 2.3 Hz), 105.01 (d, *J* = 21.4 Hz), 102.03 (d, *J* = 24.6 Hz).

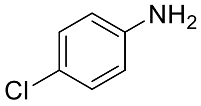

^{1}H NMR (400 MHz, Chloroform-*d*) δ 7.15 – 7.01 (m, 2H), 6.69 – 6.50 (m, 2H), 3.64 (brs, 2H).
^{13}C NMR (101 MHz, Chloroform-*d*) δ 144.97, 129.12, 123.18, 116.23.

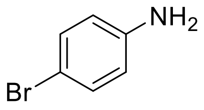

^{1}H NMR (400 MHz, Chloroform-*d*) δ 7.21 (d, *J* = 8.6 Hz, 2H), 6.53 (d, *J* = 8.6 Hz, 2H), 3.64 (brs, 2H).
^{13}C NMR (101 MHz, Chloroform-*d*) δ 145.50, 132.02, 116.75, 110.16.

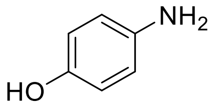

^{1}H NMR (400 MHz, DMSO-d_6) δ 8.34 (brs, 1H), 6.57 – 6.33 (m, 4H), 4.37 (brs, 2H).
^{13}C NMR (101 MHz, DMSO-d_6) δ 148.75, 141.08, 116.05, 115.76.

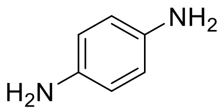

^{1}H NMR (400 MHz, Chloroform-*d*) δ 6.56 (s, 4H), 3.32 (brs, 4H).
^{13}C NMR (101 MHz, Chloroform-*d*) δ 138.61, 116.74.

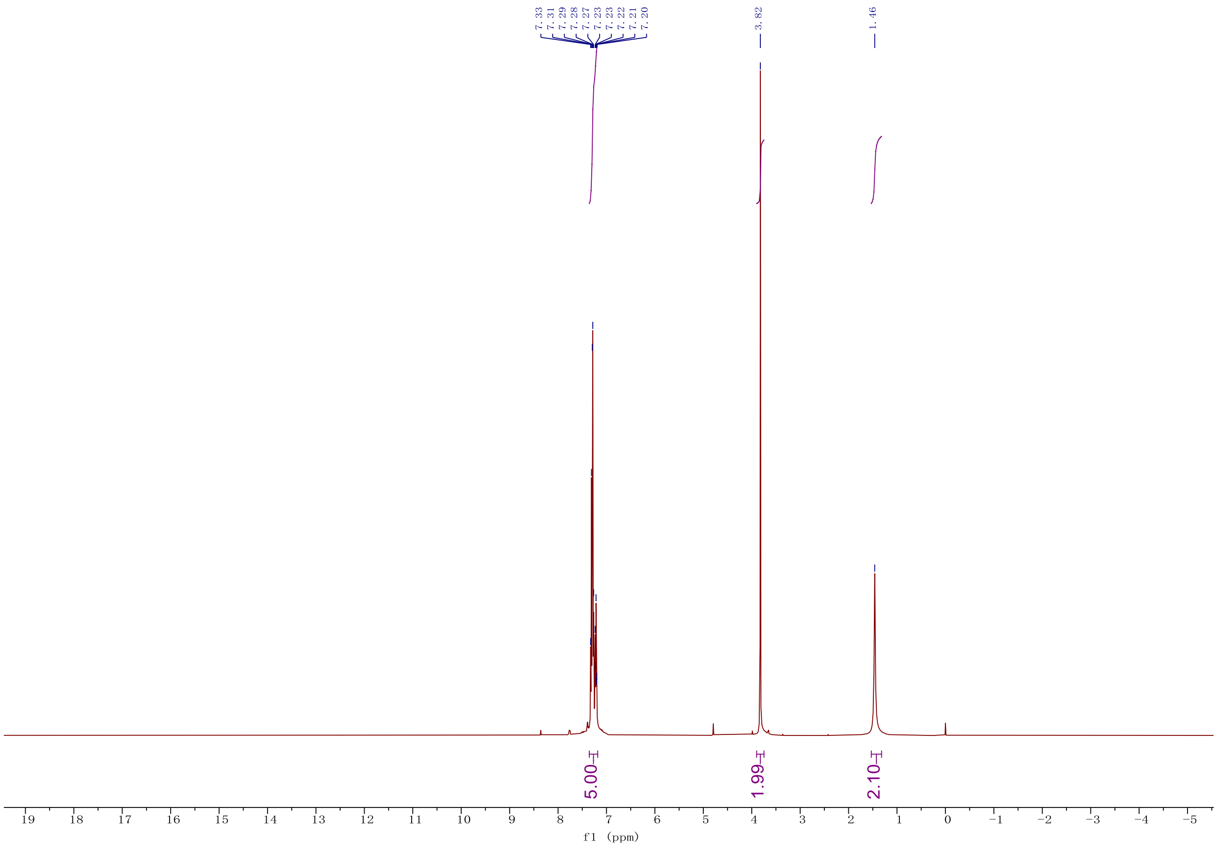

Figure S5 ^{1}H NMR for benzylamine

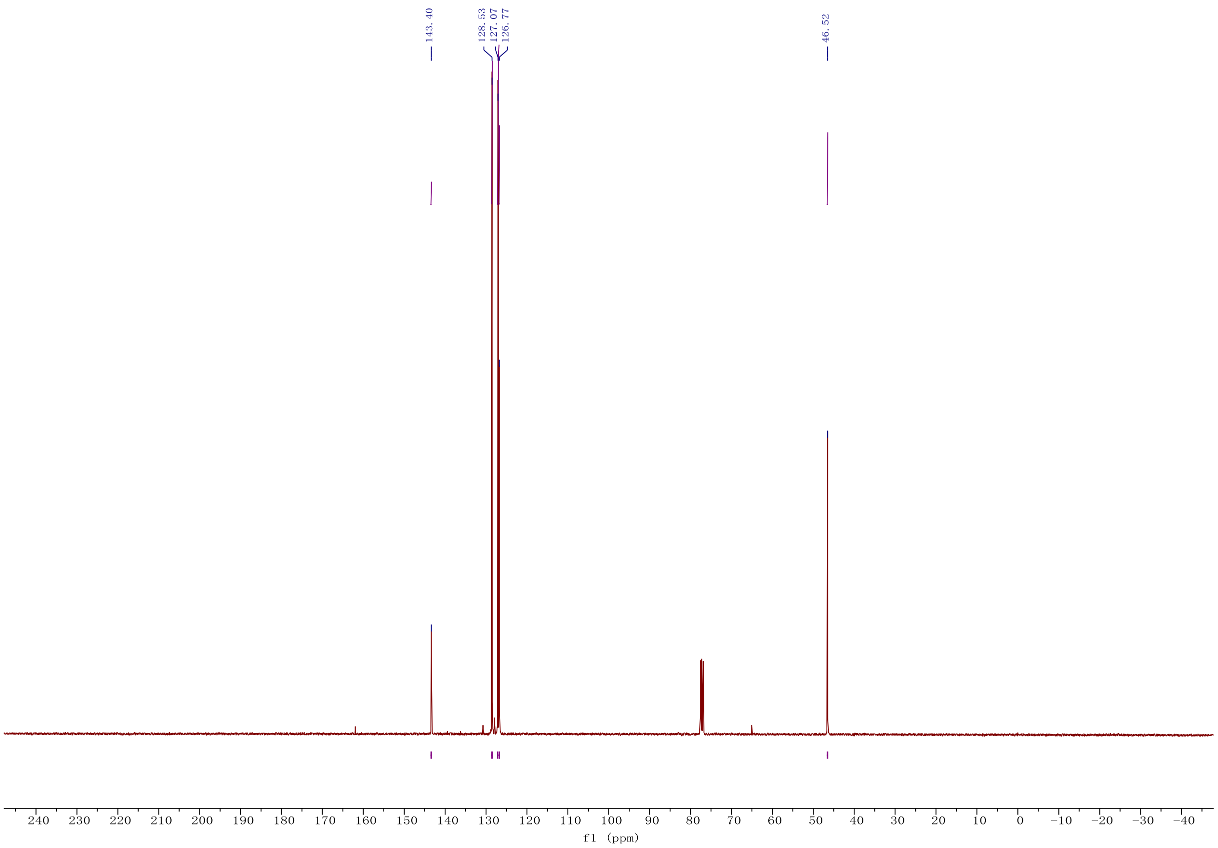

Figure S6 ^{13}C NMR for benzylamine

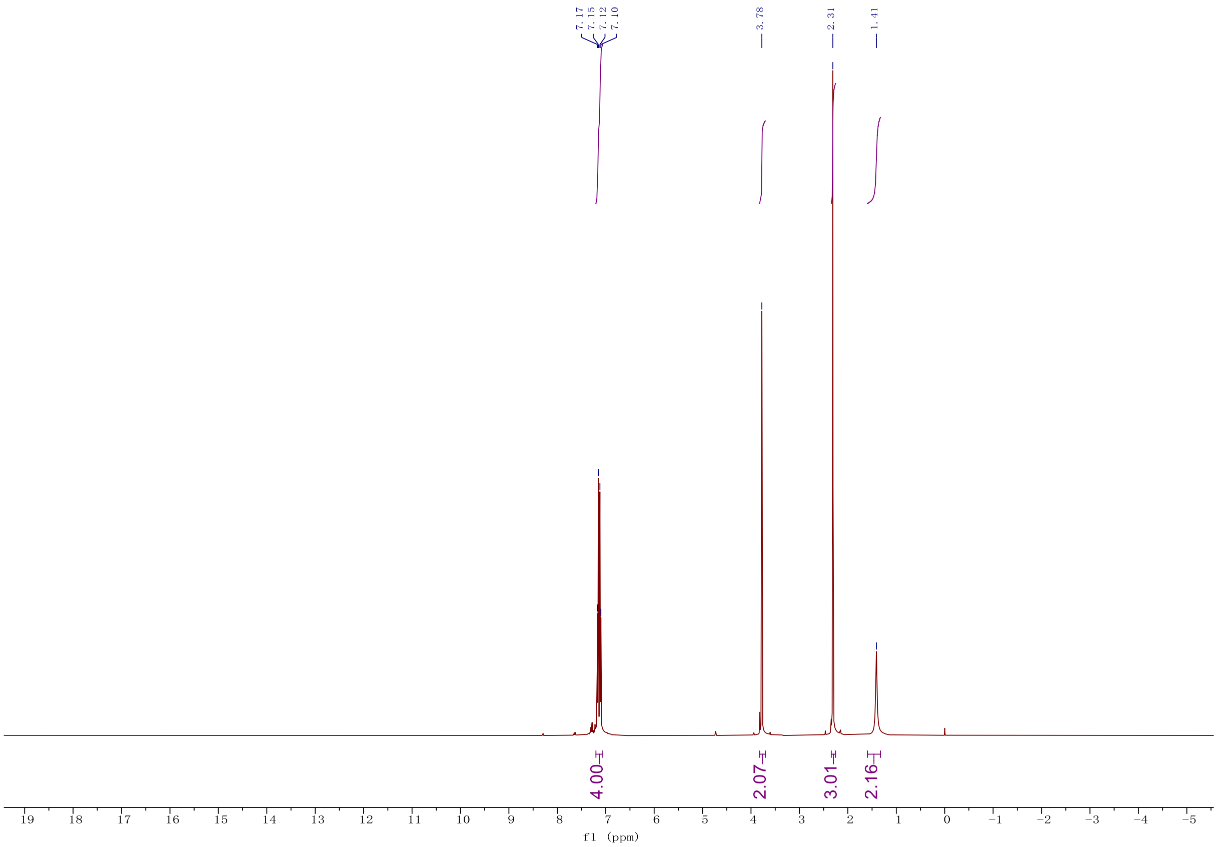

Figure S7 ¹H NMR for 4-methylbenzylamine

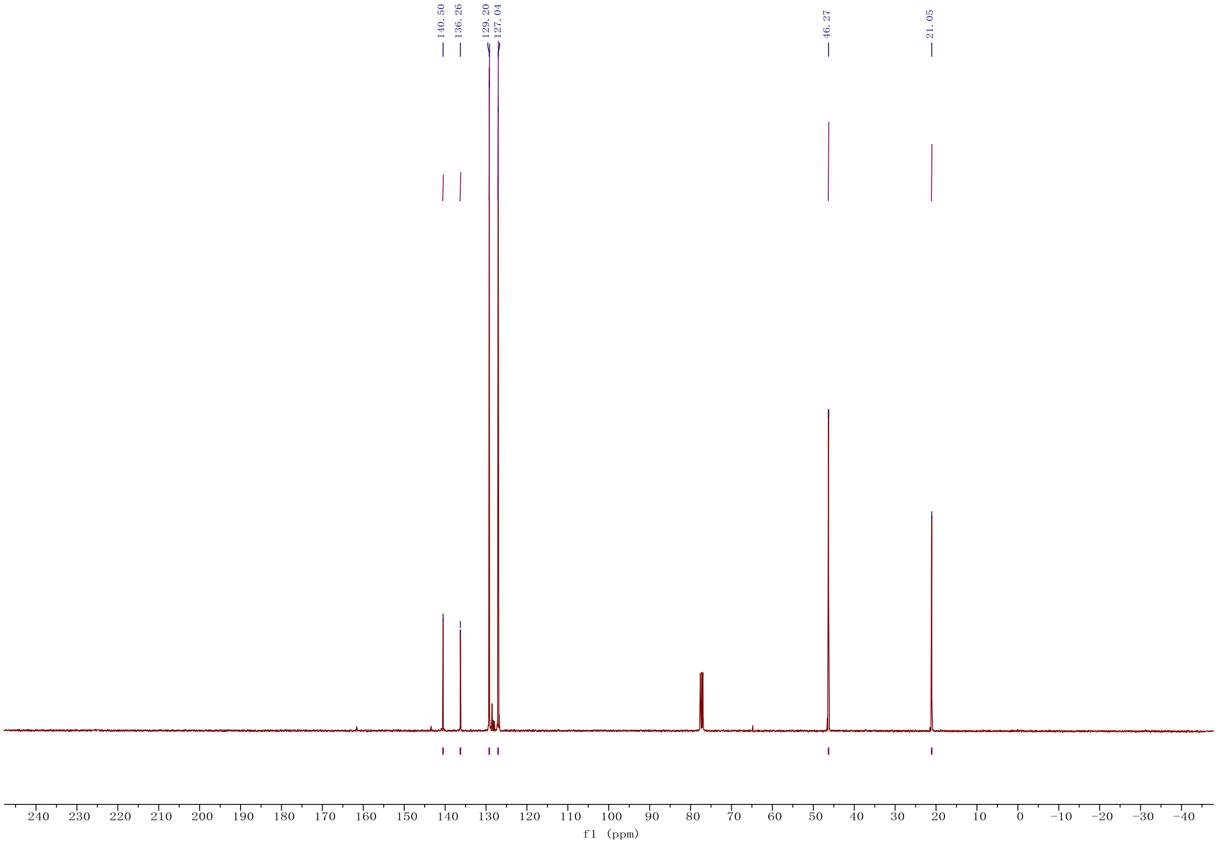

Figure S8 ¹³C NMR for 4-methylbenzylamine

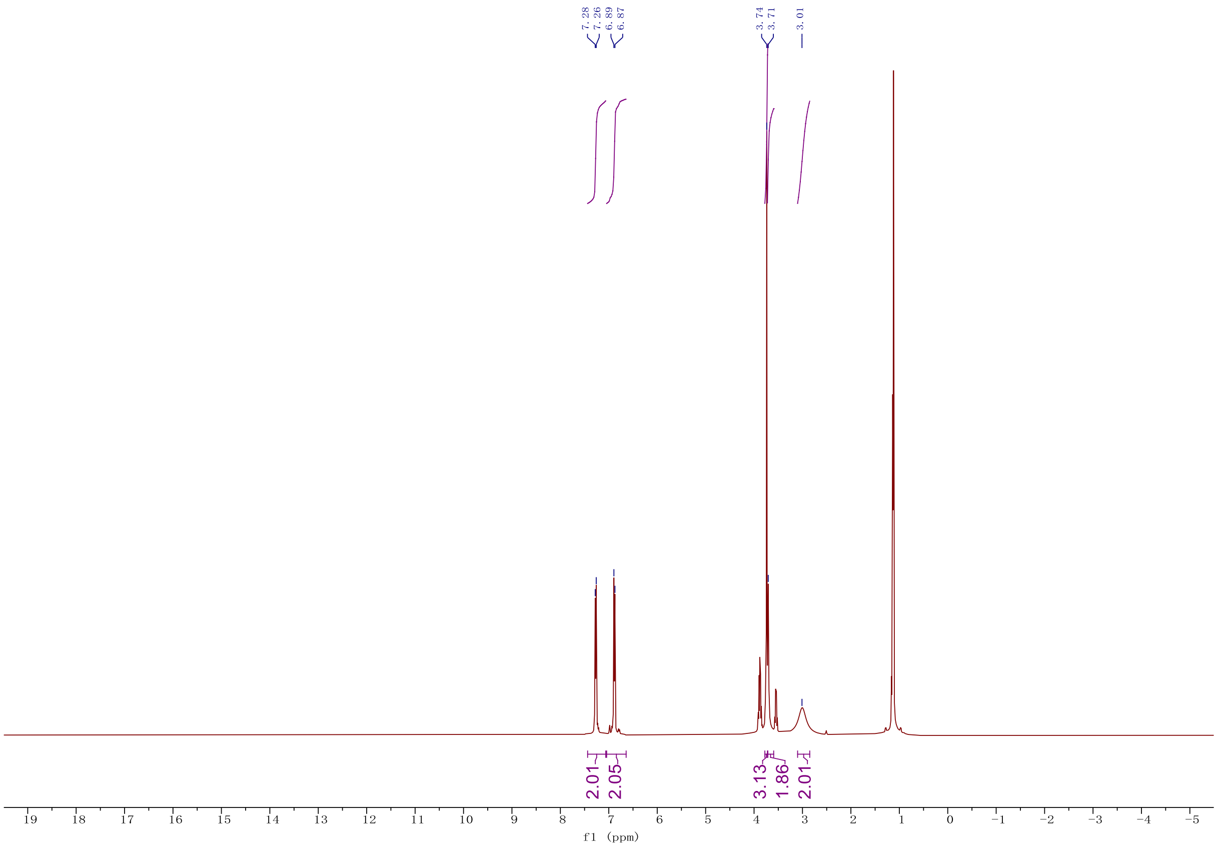

Figure S9 ^{1}H NMR for (4-methoxyphenyl)methanamine

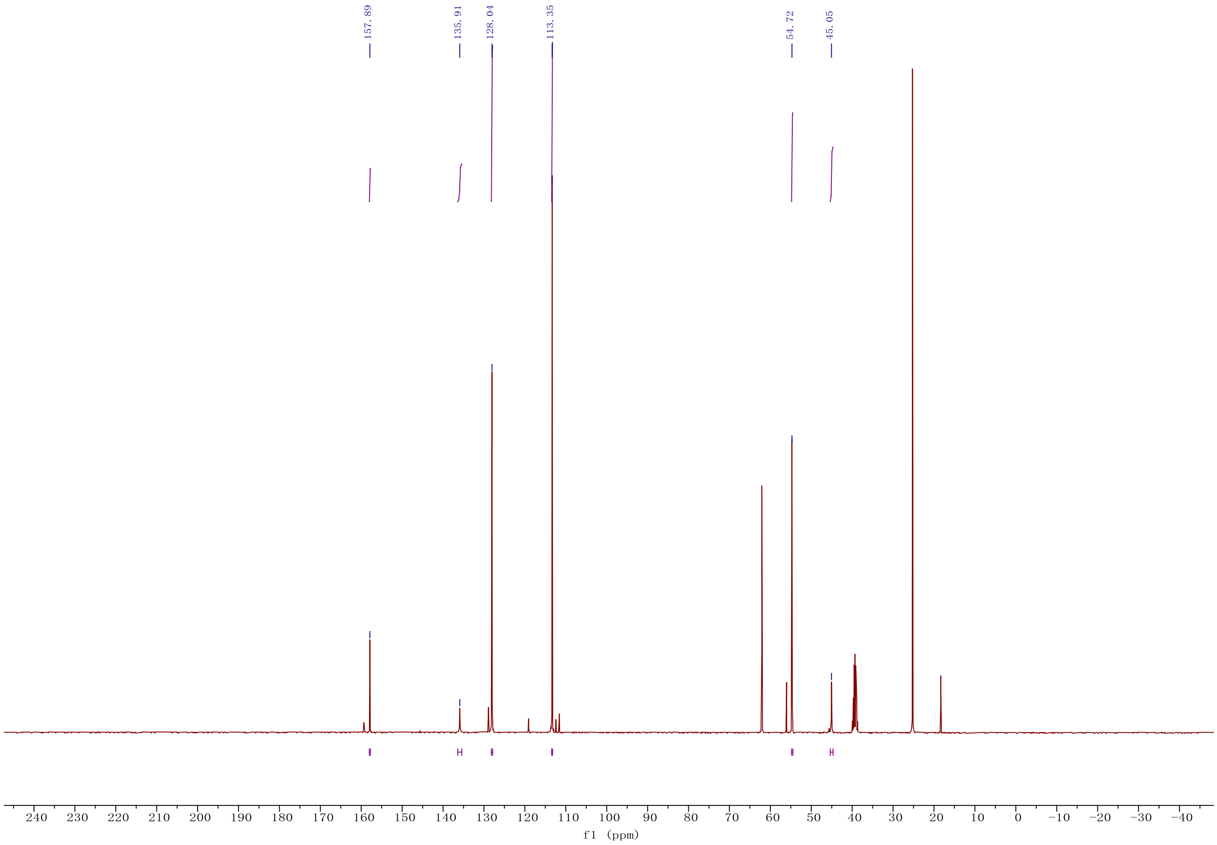

Figure S10 ^{13}C NMR for (4-methoxyphenyl)methanamine

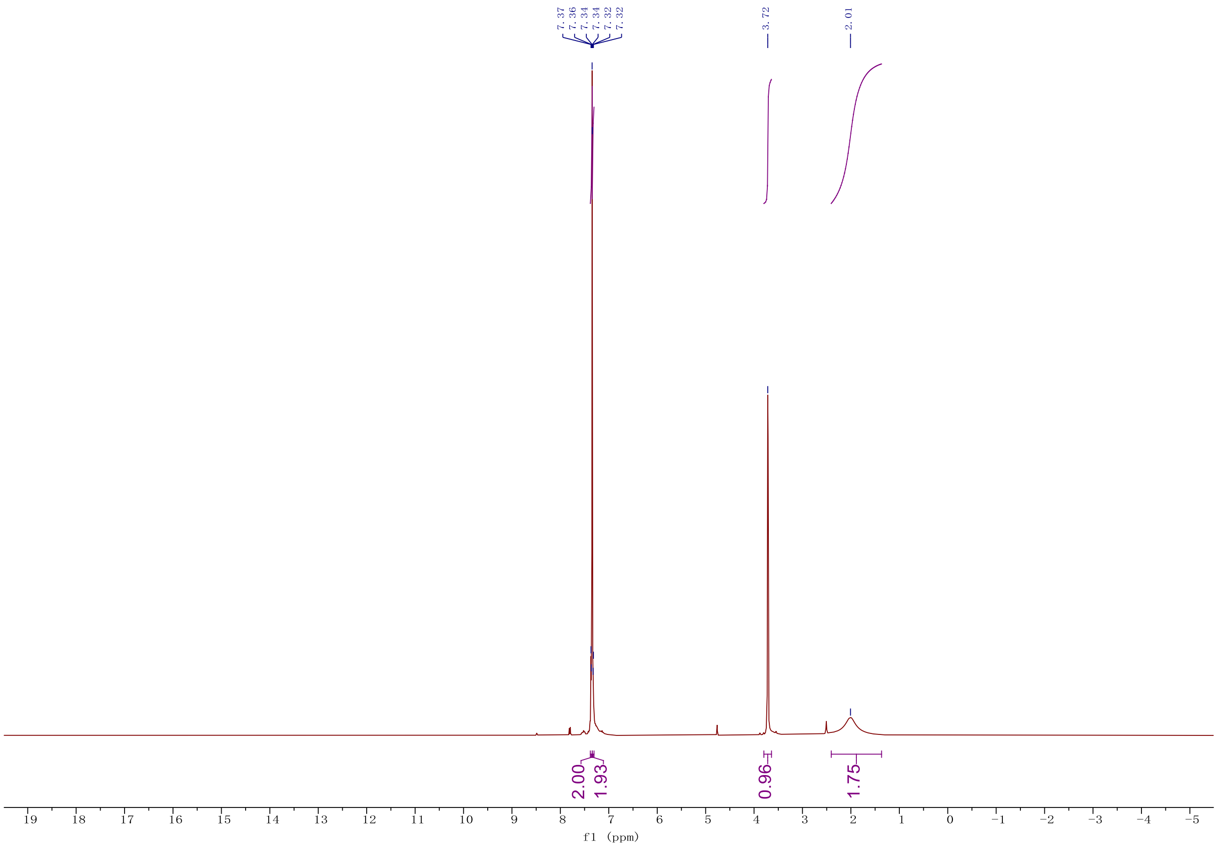

FigureS 11 ^{1}H NMR for 4-chlorobenzylamine

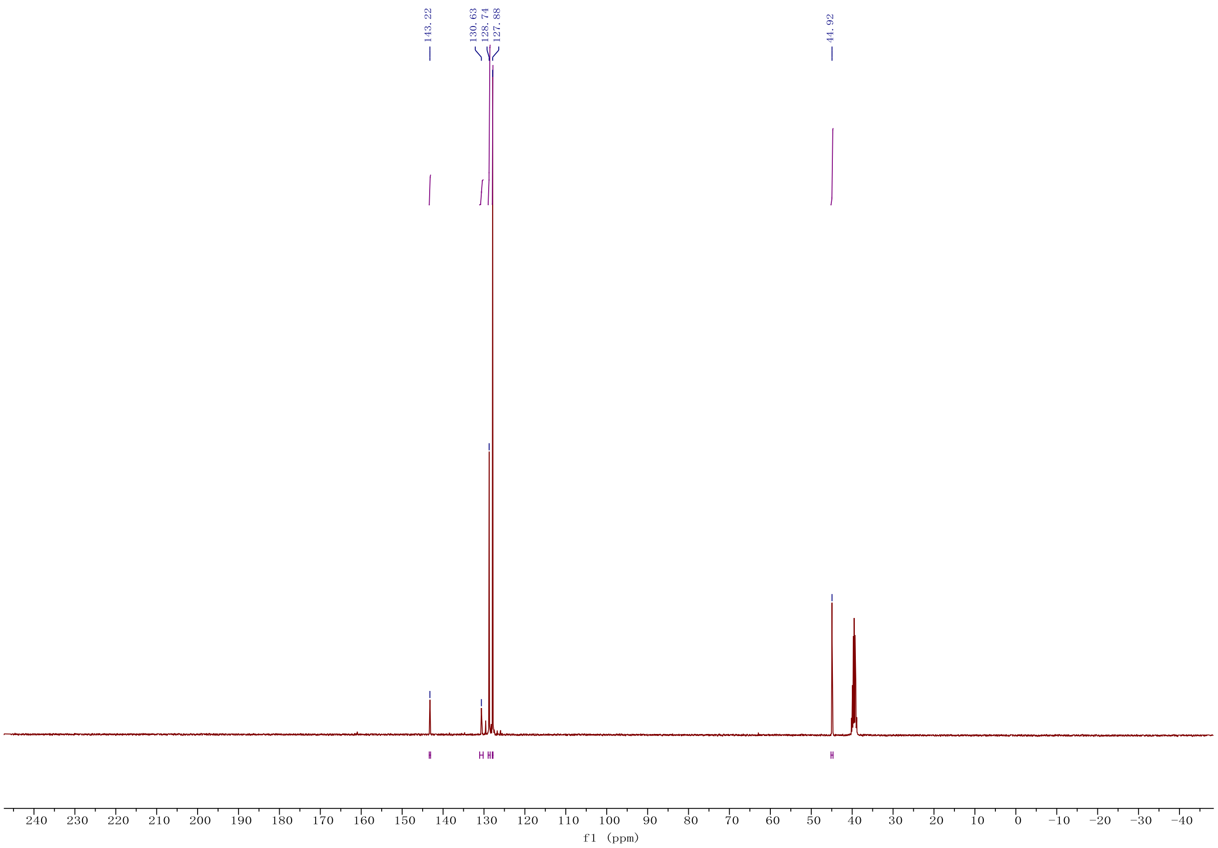

Figure S12 ^{13}C NMR for 4-chlorobenzylamine

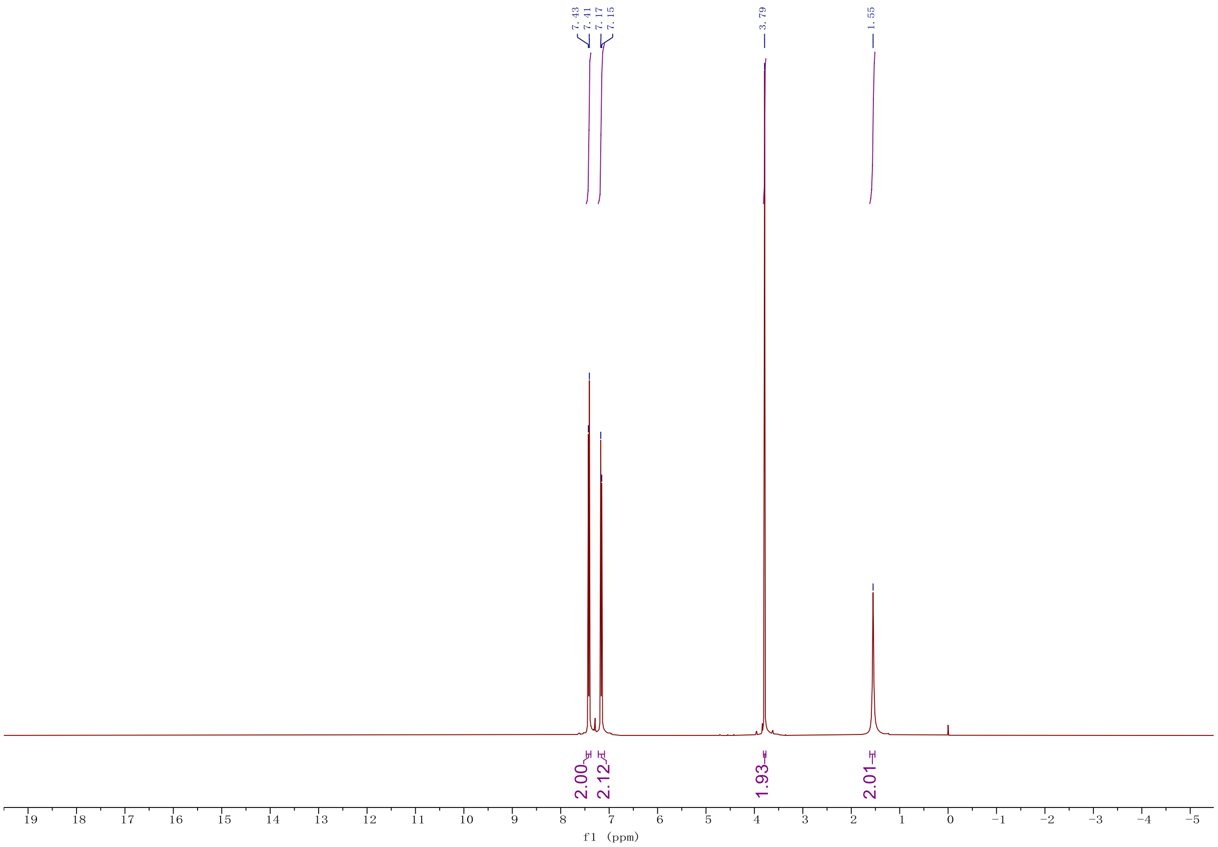

Figure S13 ^{1}H NMR for 4-bromobenzylamine

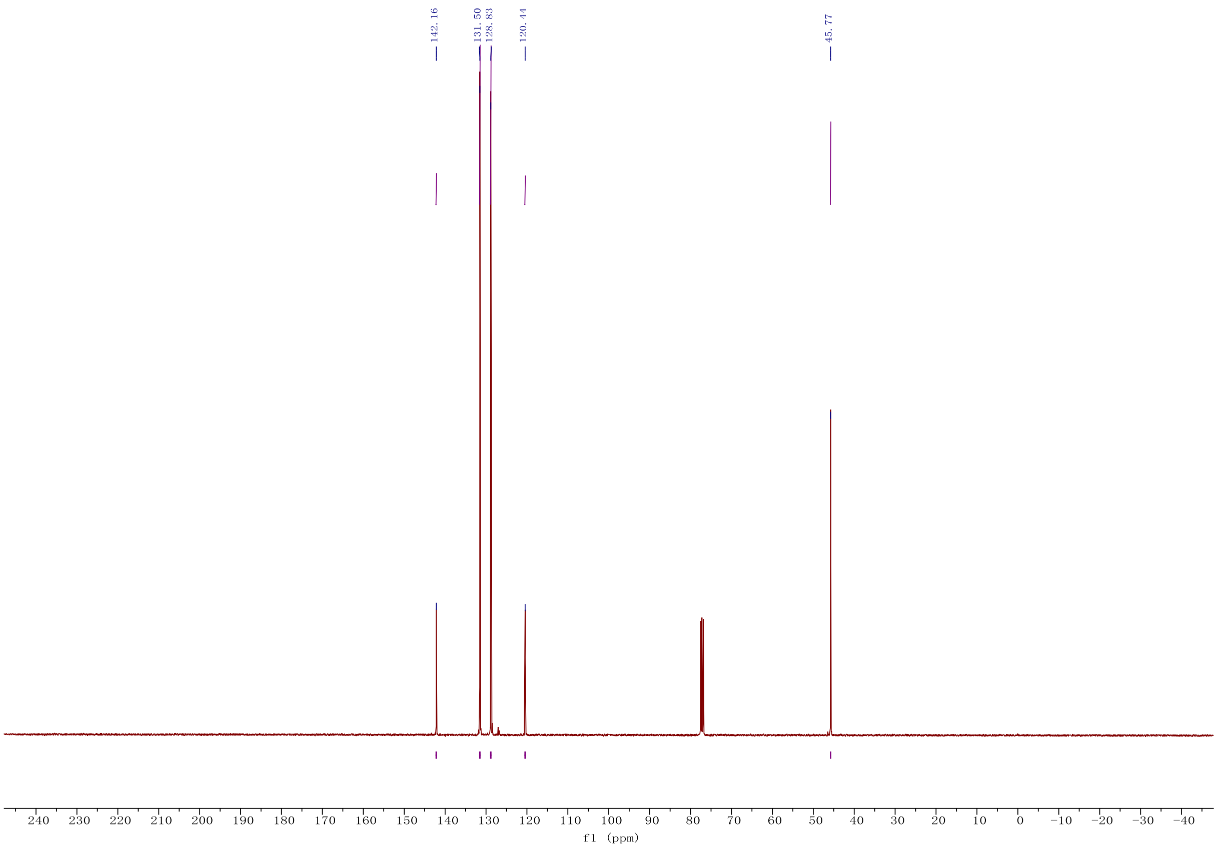

Figure S14 ^{13}C NMR for 4-bromobenzylamine

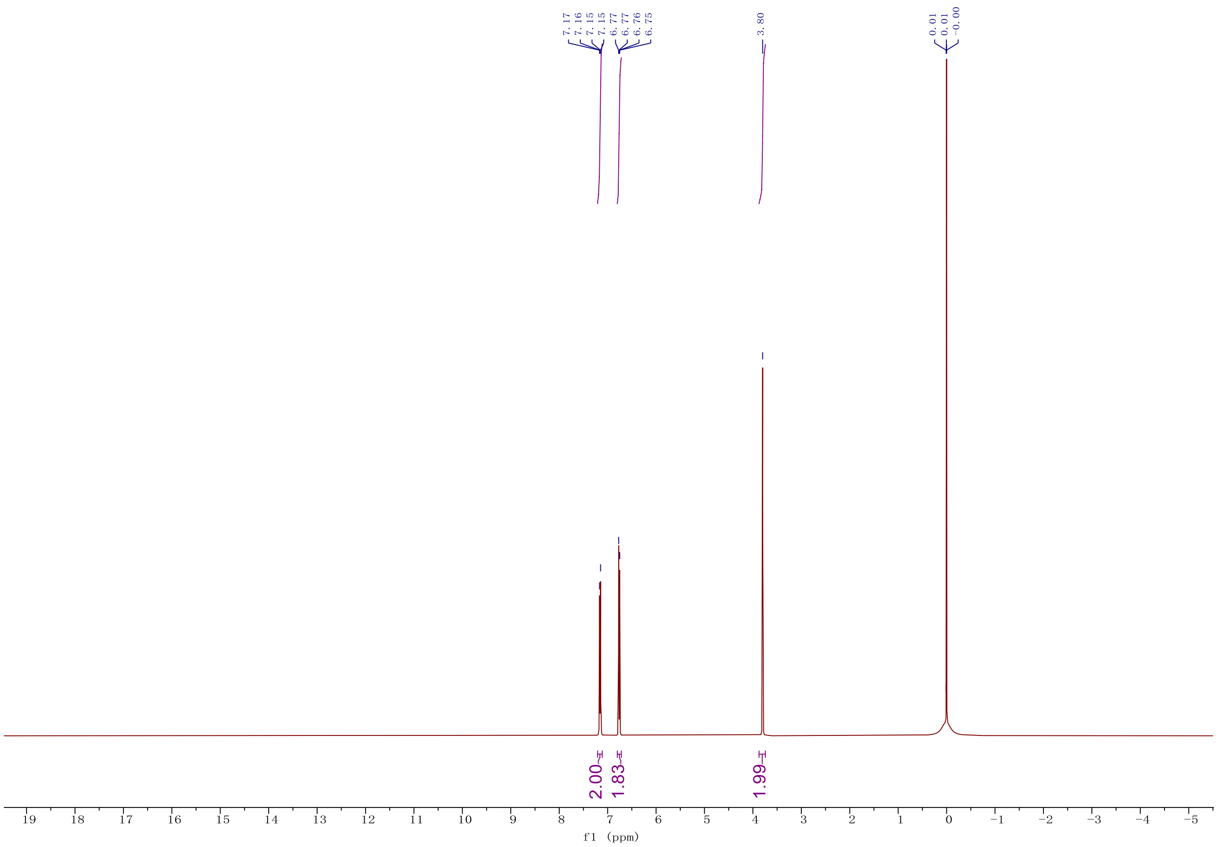

Figure S15 ^{1}H NMR for 4-hydroxybenzylamine

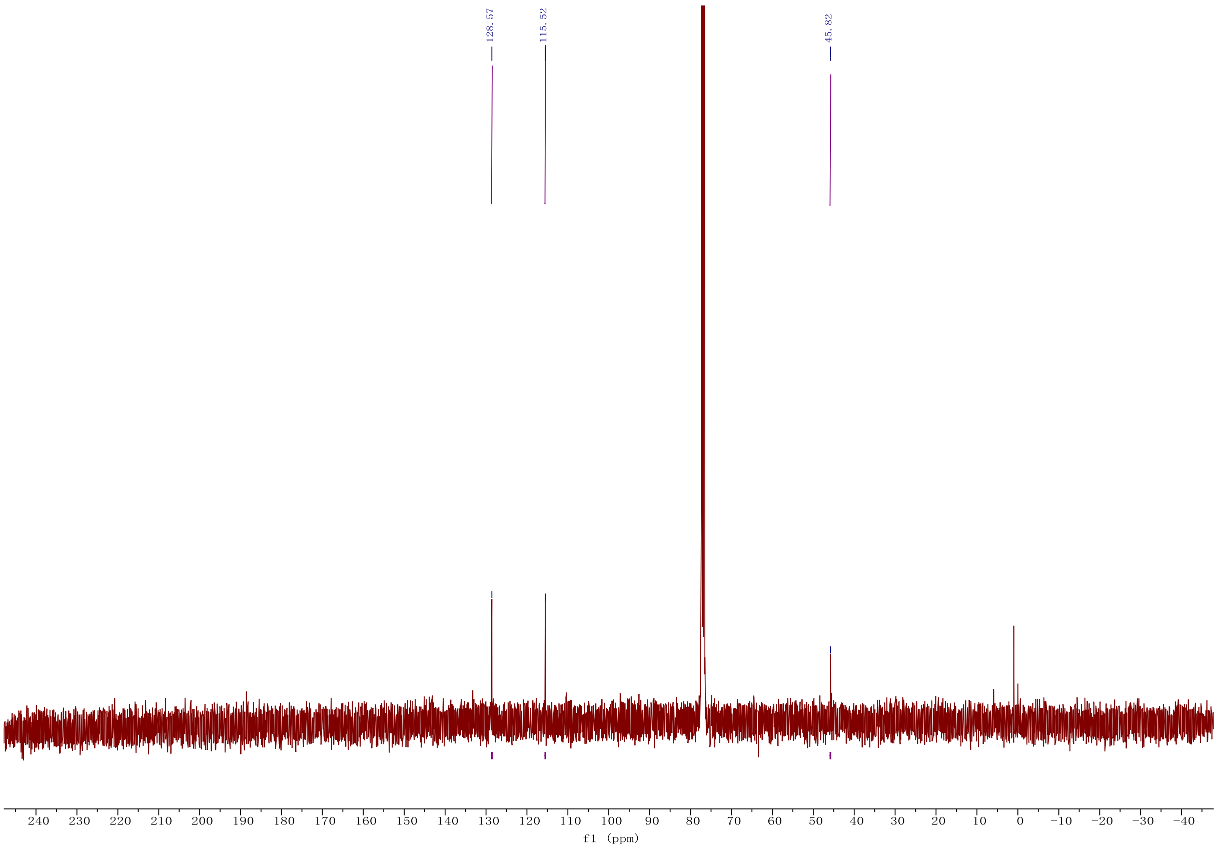

Figure S16 ^{13}C NMR for 4-hydroxybenzylamine

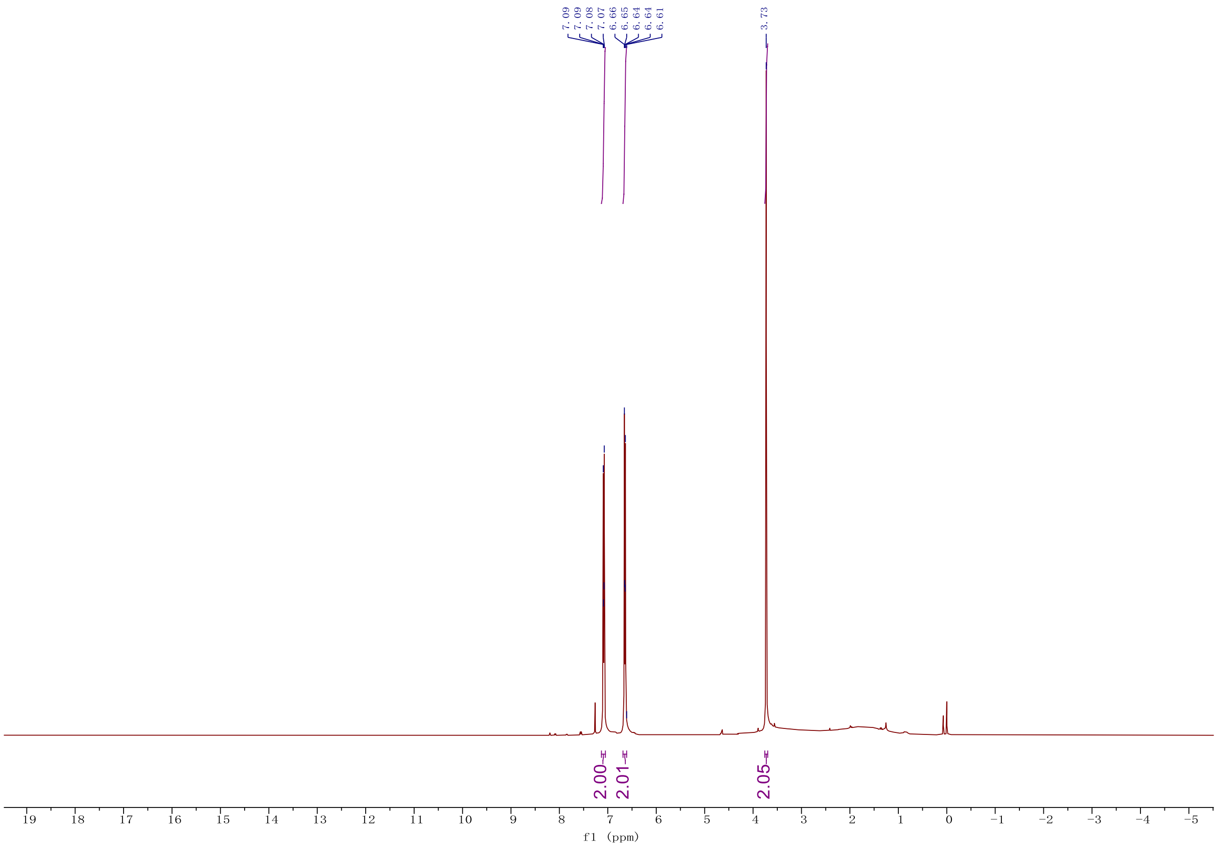

Figure S17 ^{1}H NMR for 4-(aminomethyl)aniline

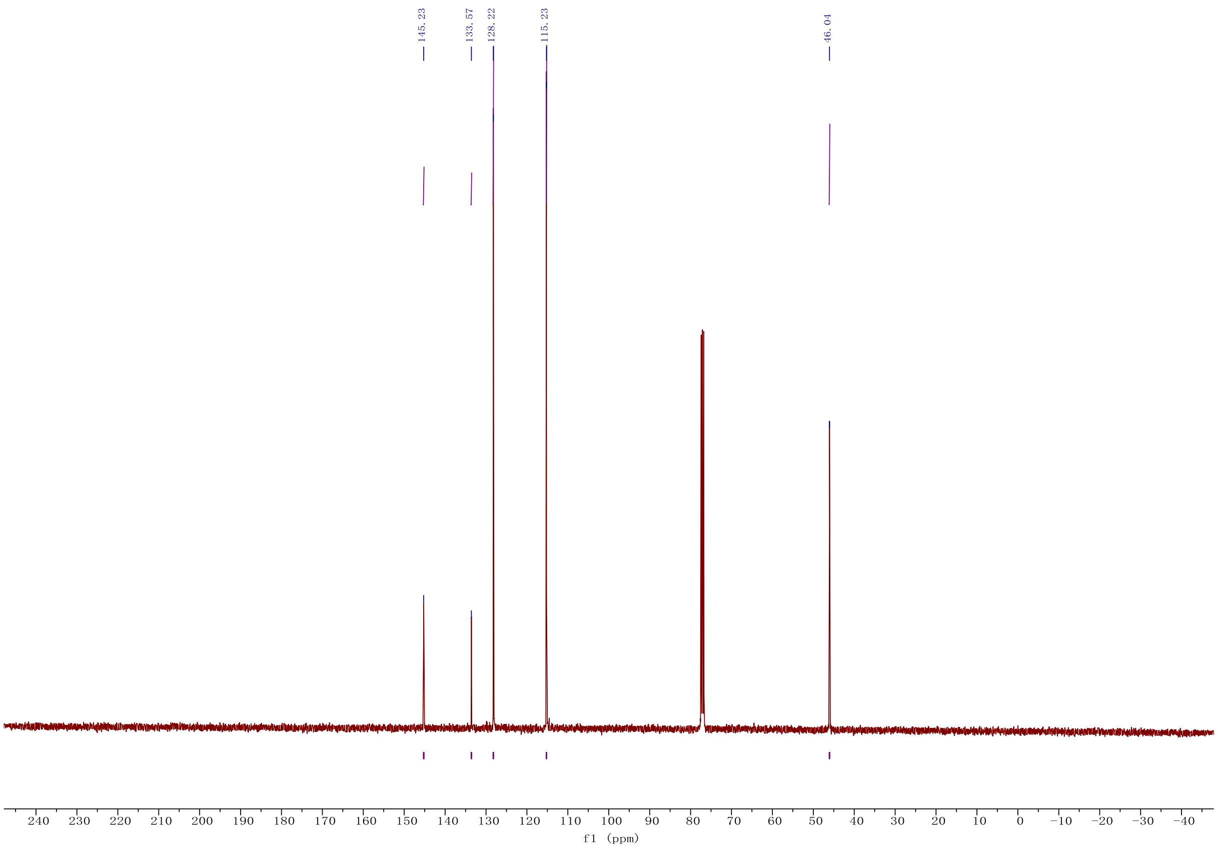

Figure S18 ^{13}C NMR for 4-(aminomethyl)aniline

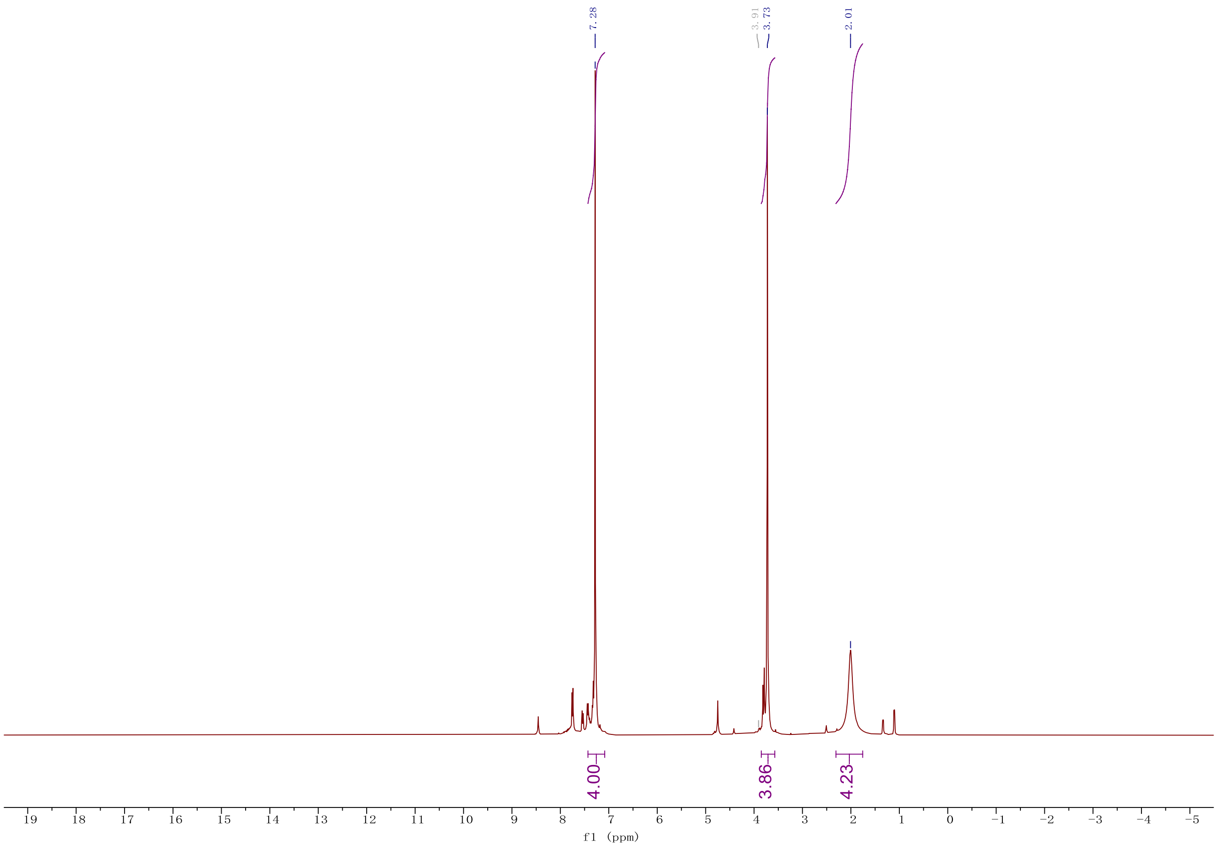

Figure S19 ^{1}H NMR for 1,4-phenylenedimethanamine

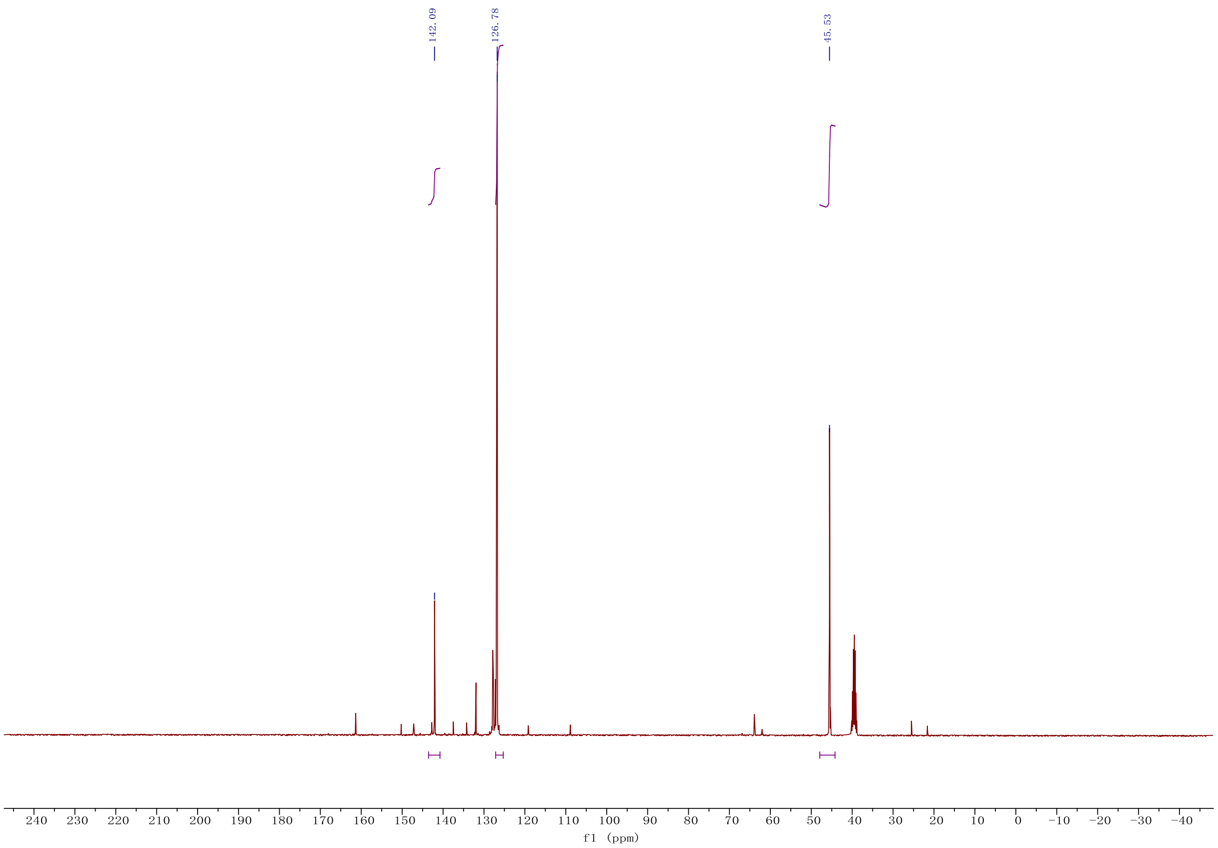

Figure S20 ^{13}C NMR for 1,4-phenylenedimethanamine

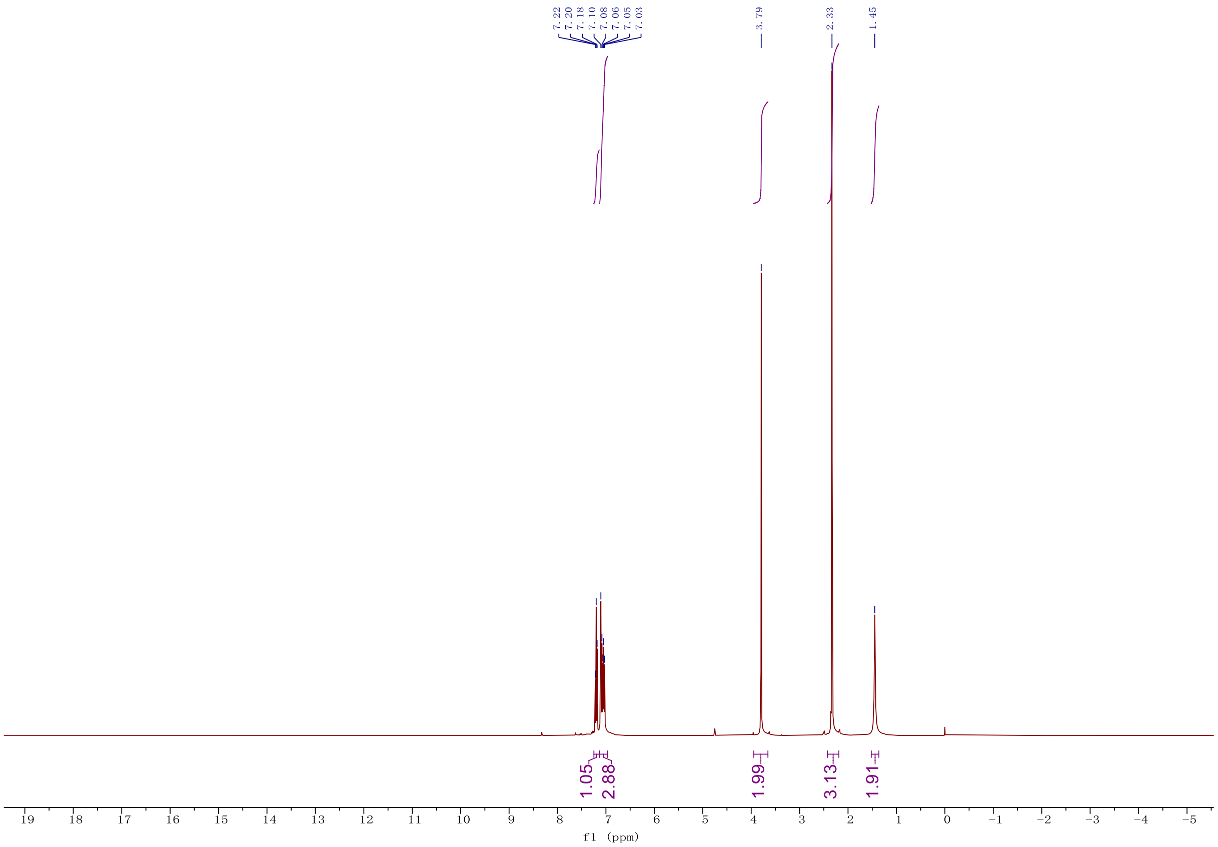

Figure S21 ^{1}H NMR for 3-methylbenzylamine

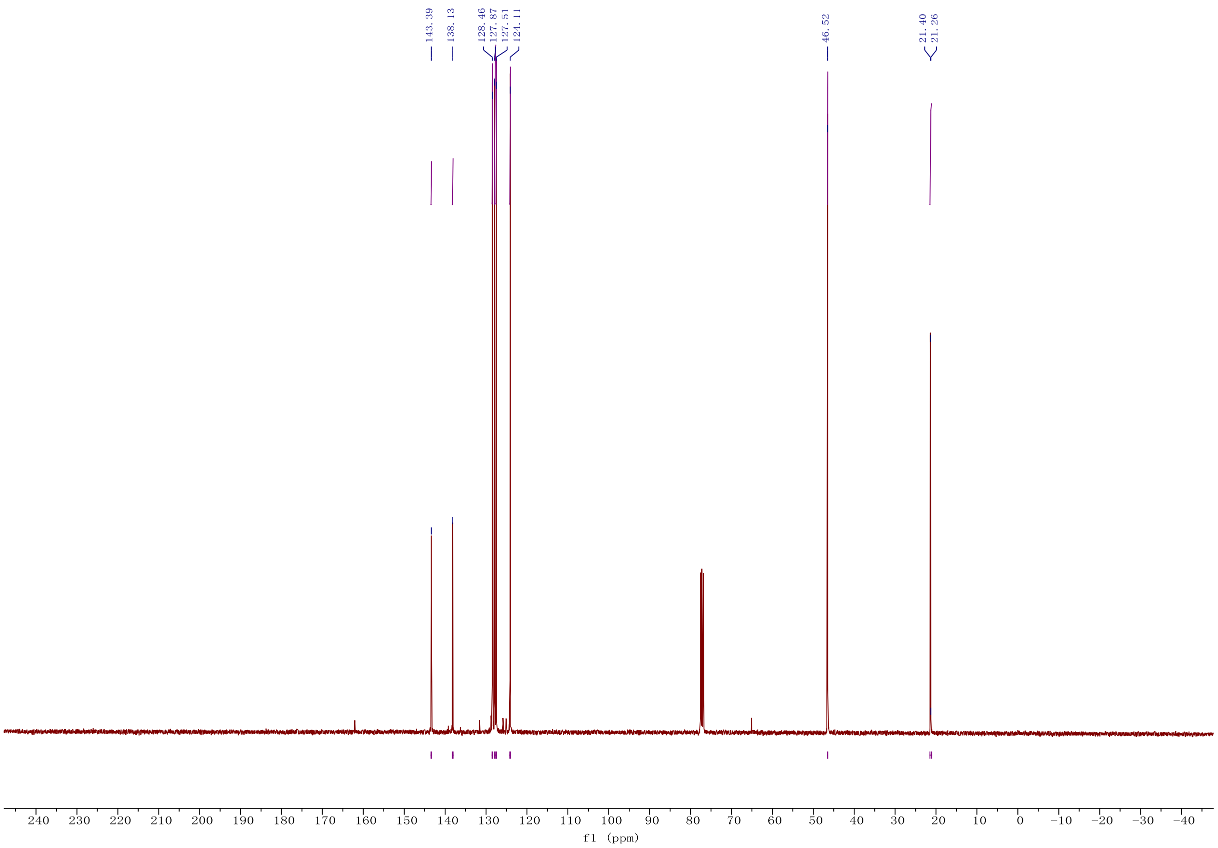

Figure S22 ^{13}C NMR for 3-methylbenzylamine

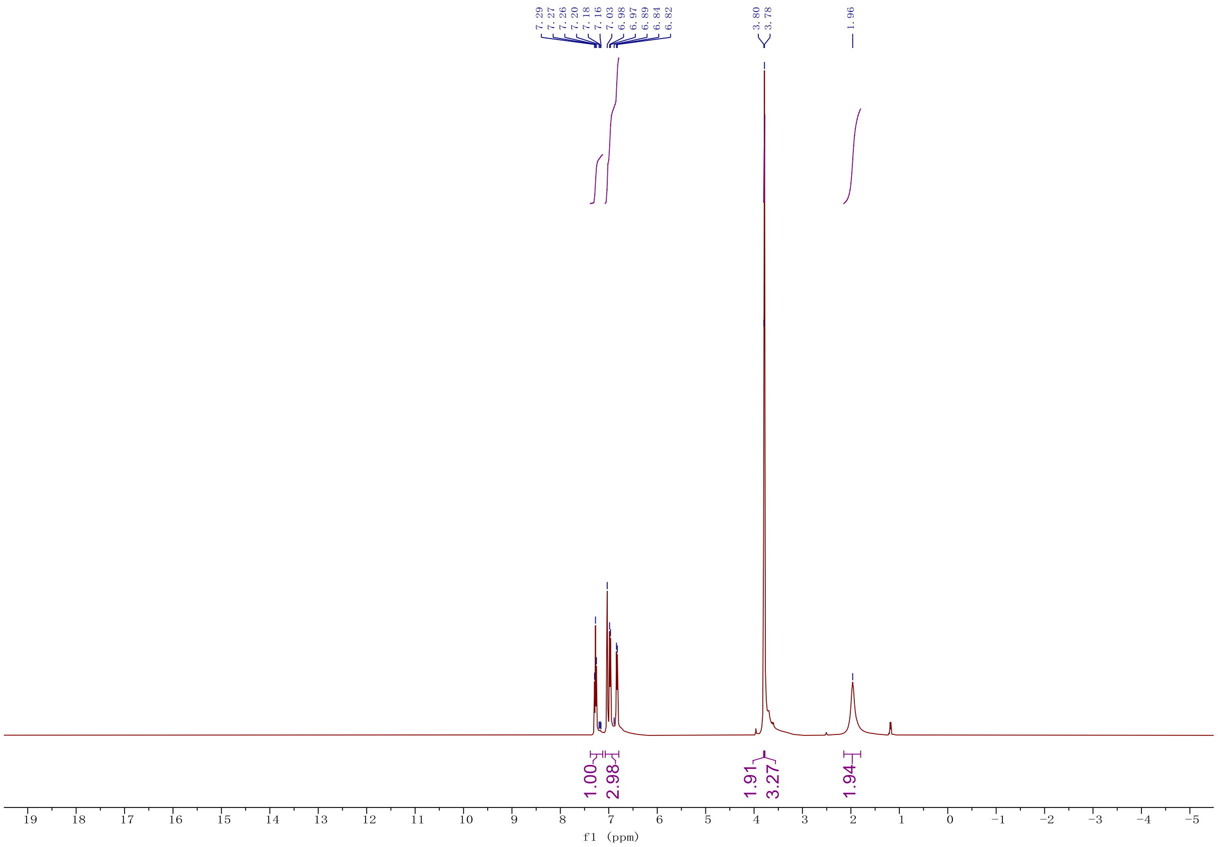

Figure S23 ^{1}H NMR for (3-methoxyphenyl)methanamine

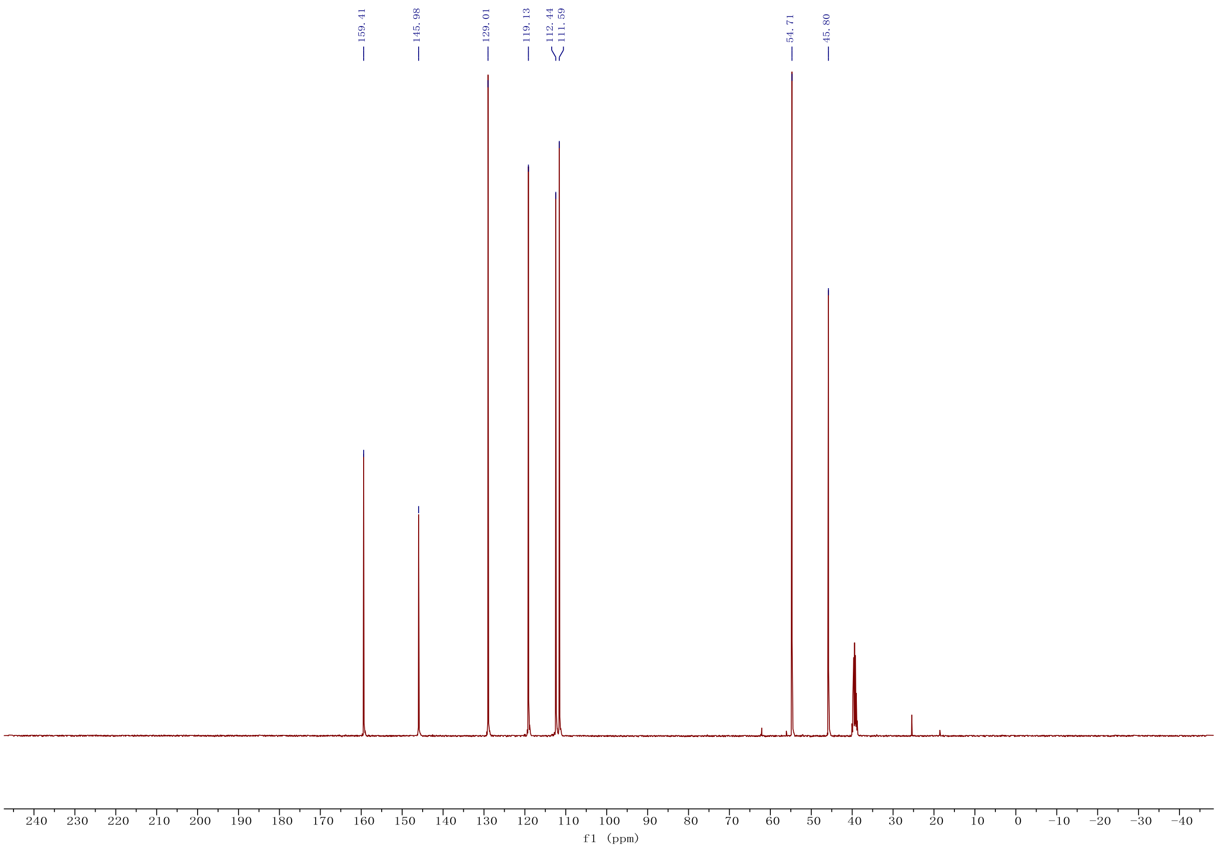

Figure S24 ^{13}C NMR for (3-methoxyphenyl)methanamine

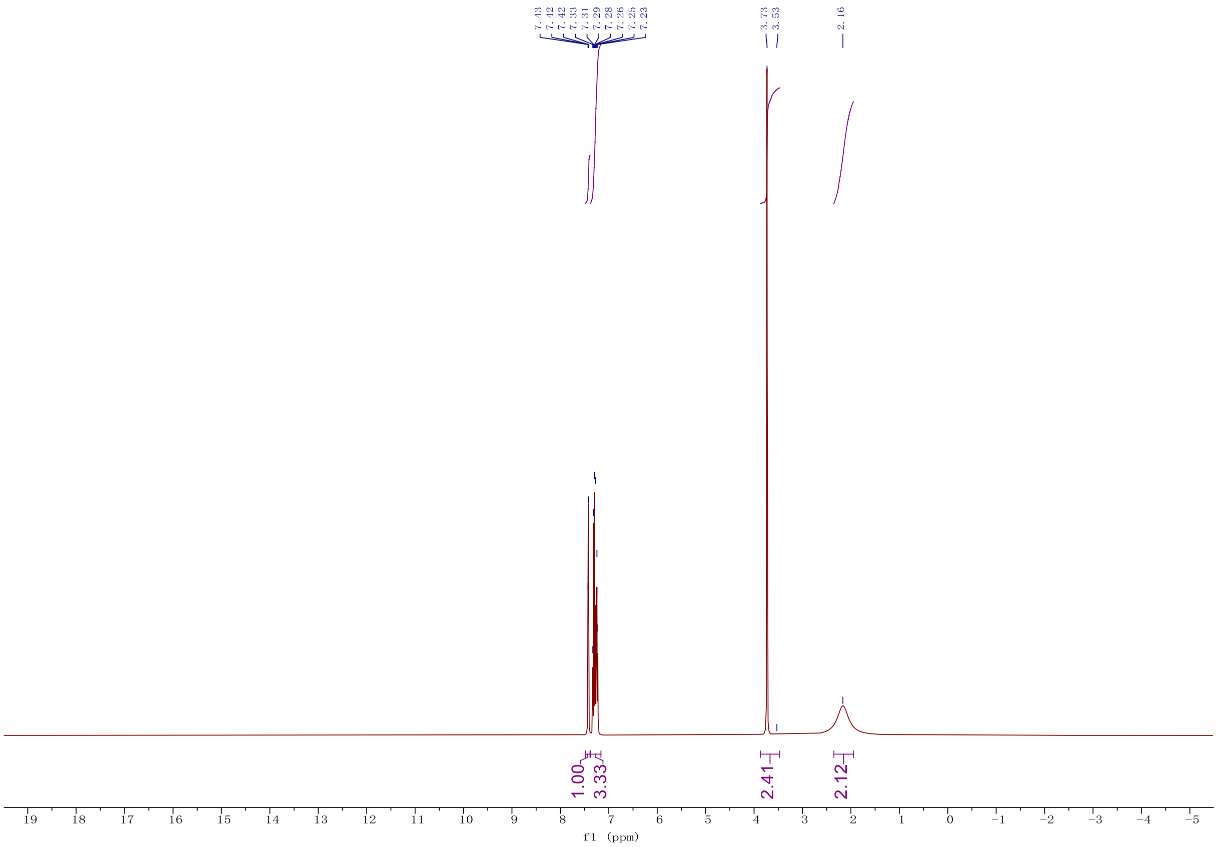

Figure S25 ^{1}H NMR for 3-chlorobenzylamine

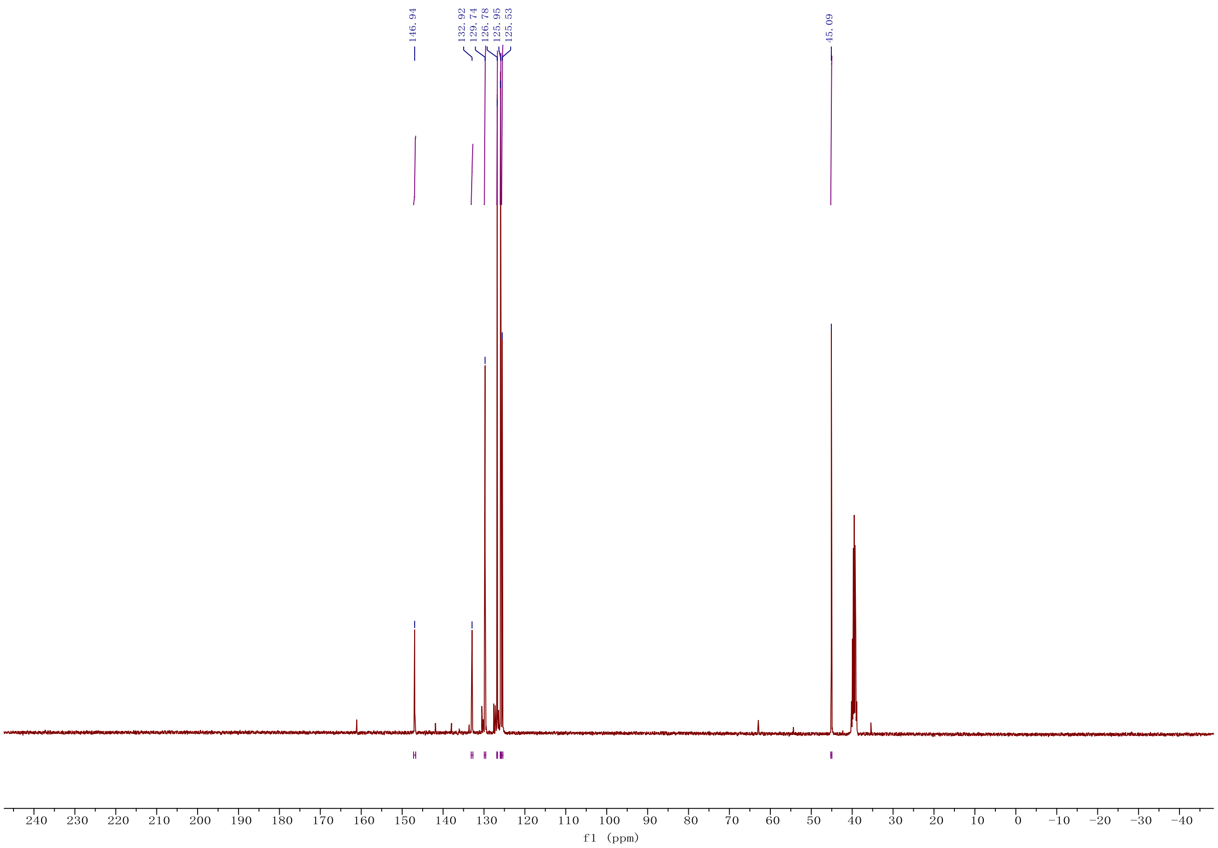

Figure S26 ^{13}C NMR for 3-chlorobenzylamine

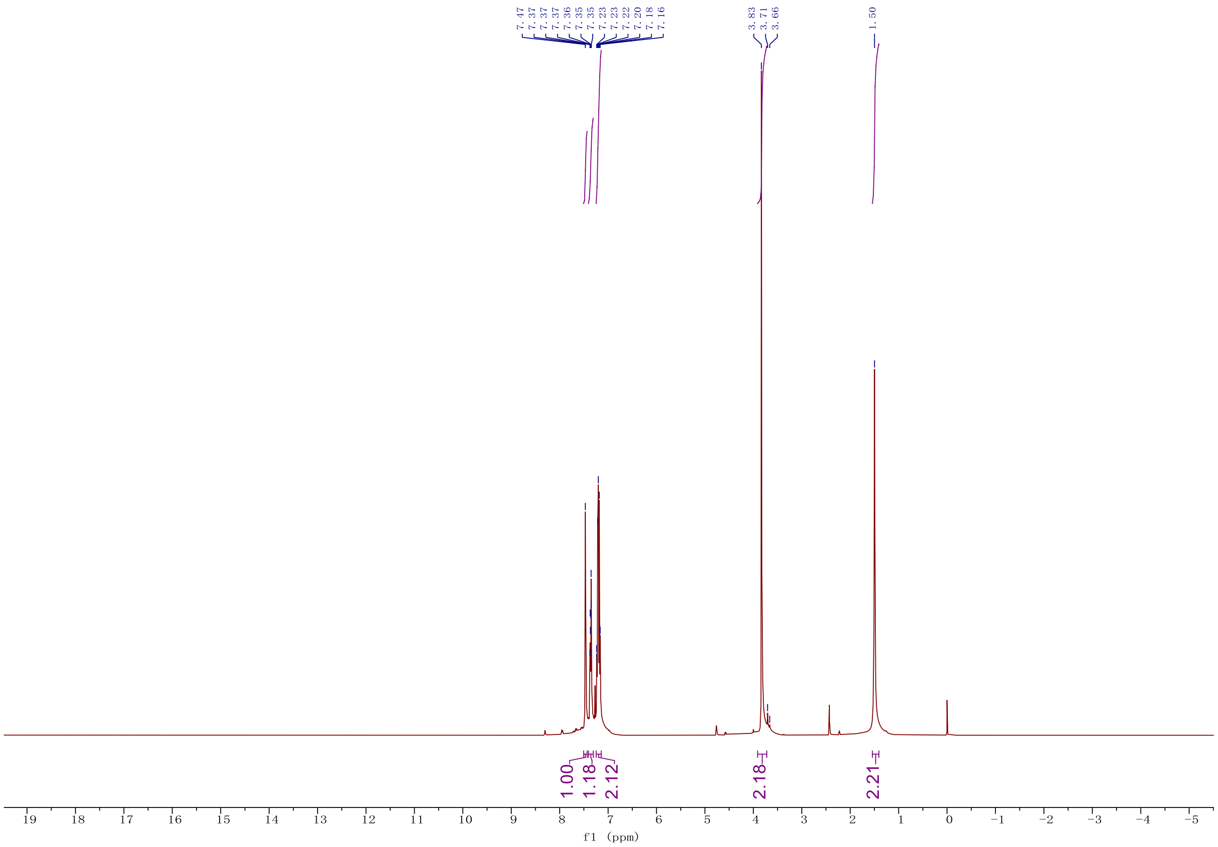

Figure S27 ^{1}H NMR for 3-bromobenzylamine

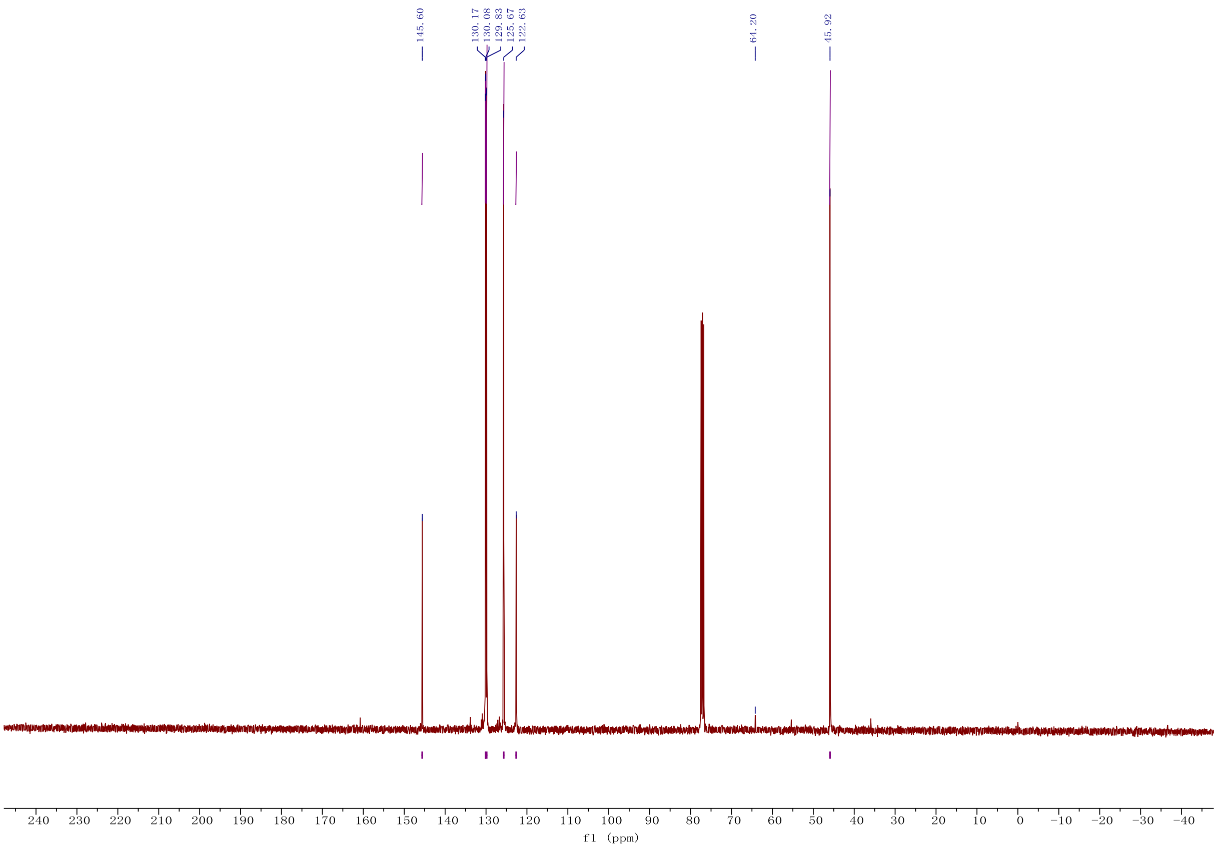

Figure S28 ^{13}C NMR for 3-bromobenzylamine

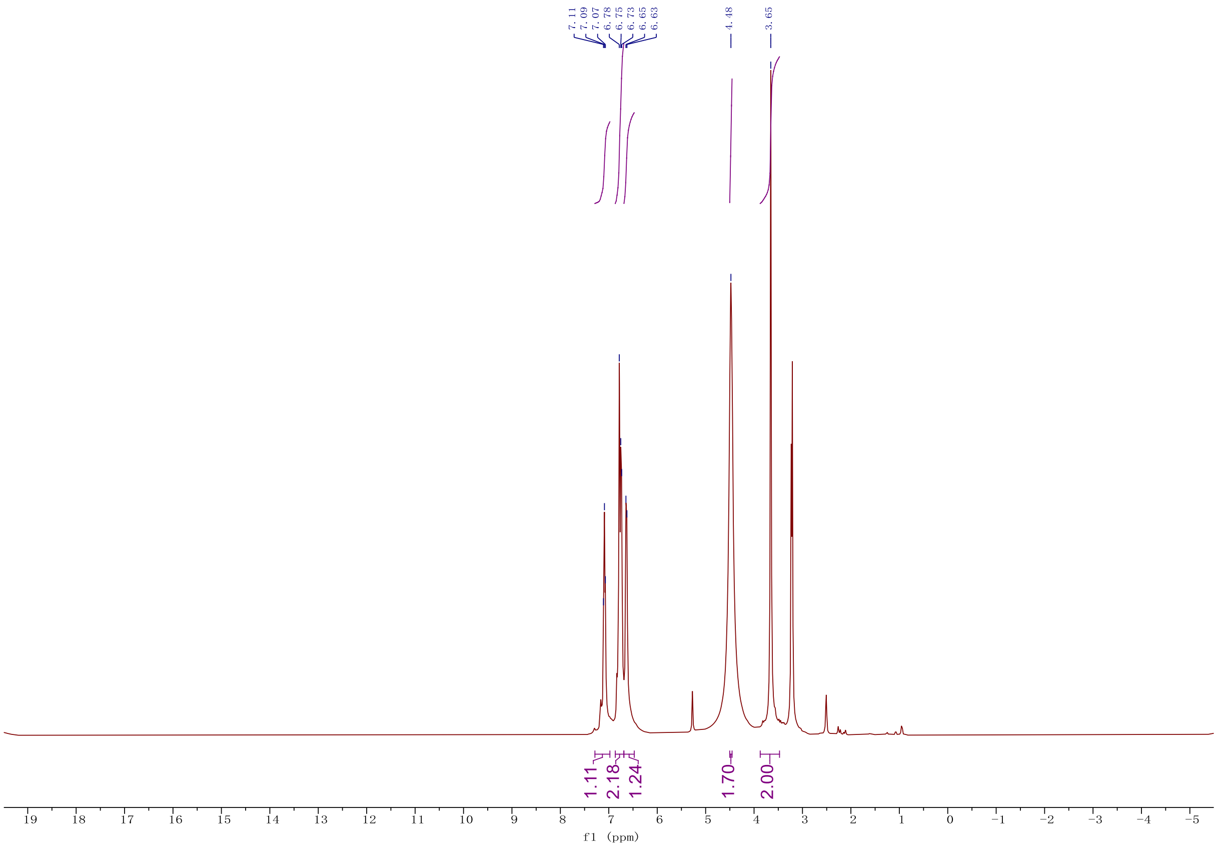

Figure S29 ^{1}H NMR for 3-hydroxybenzylamine

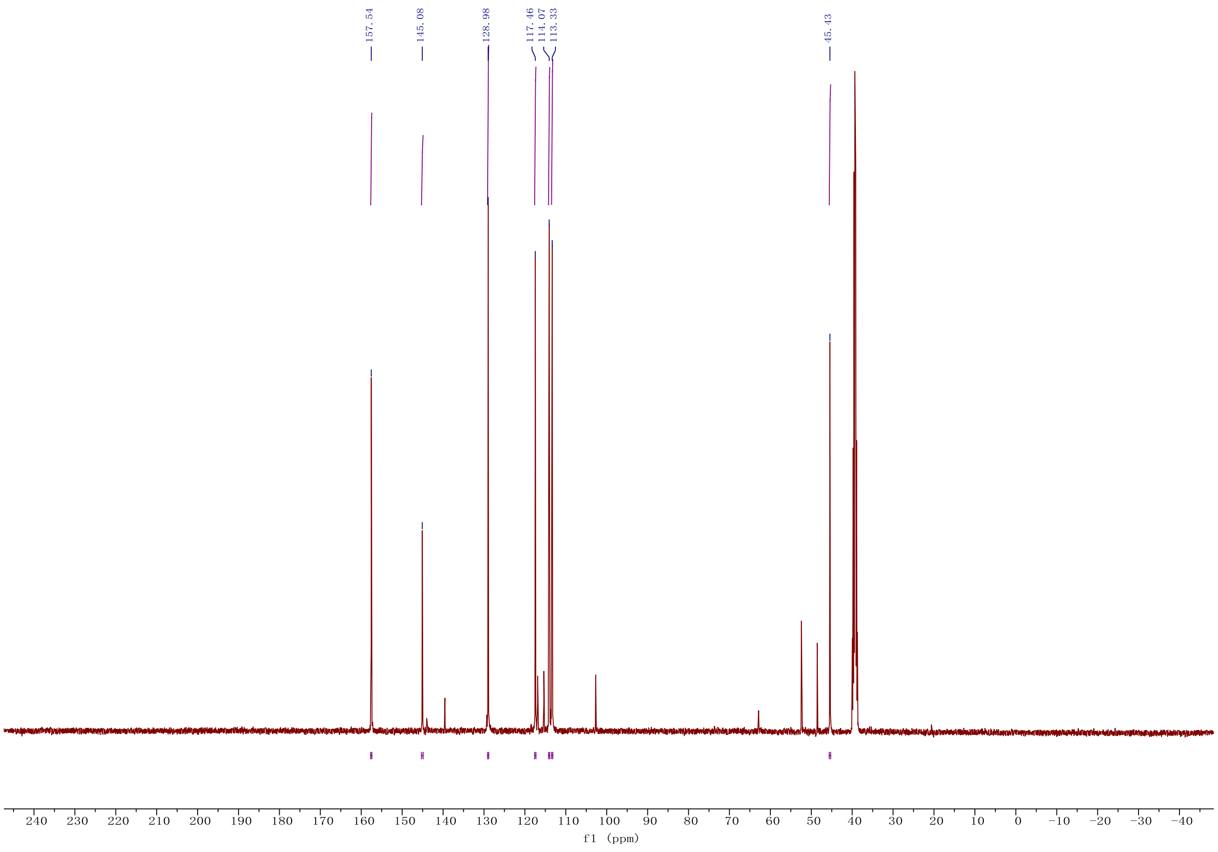

Figure S30 ^{13}C NMR for 3-hydroxybenzylamine

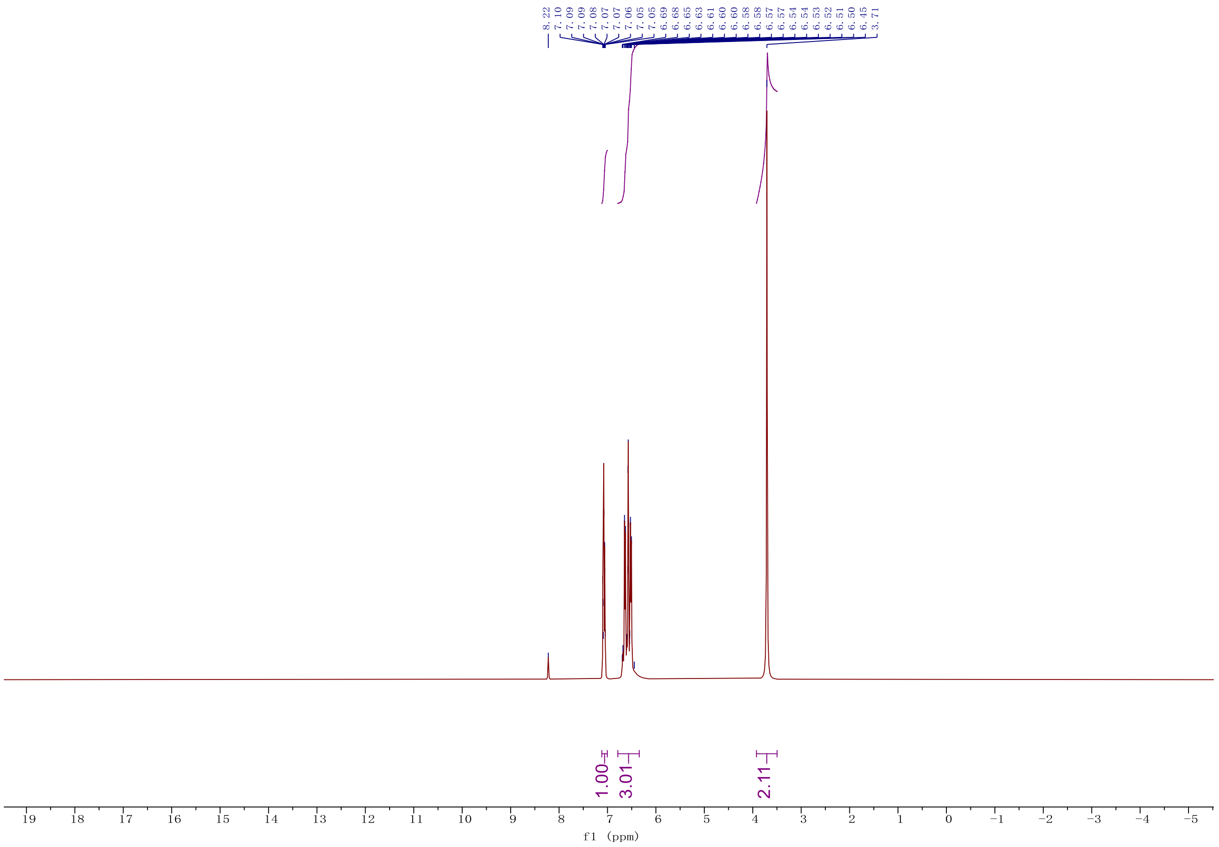

Figure S31 ^{1}H NMR for 3-(aminomethyl)aniline

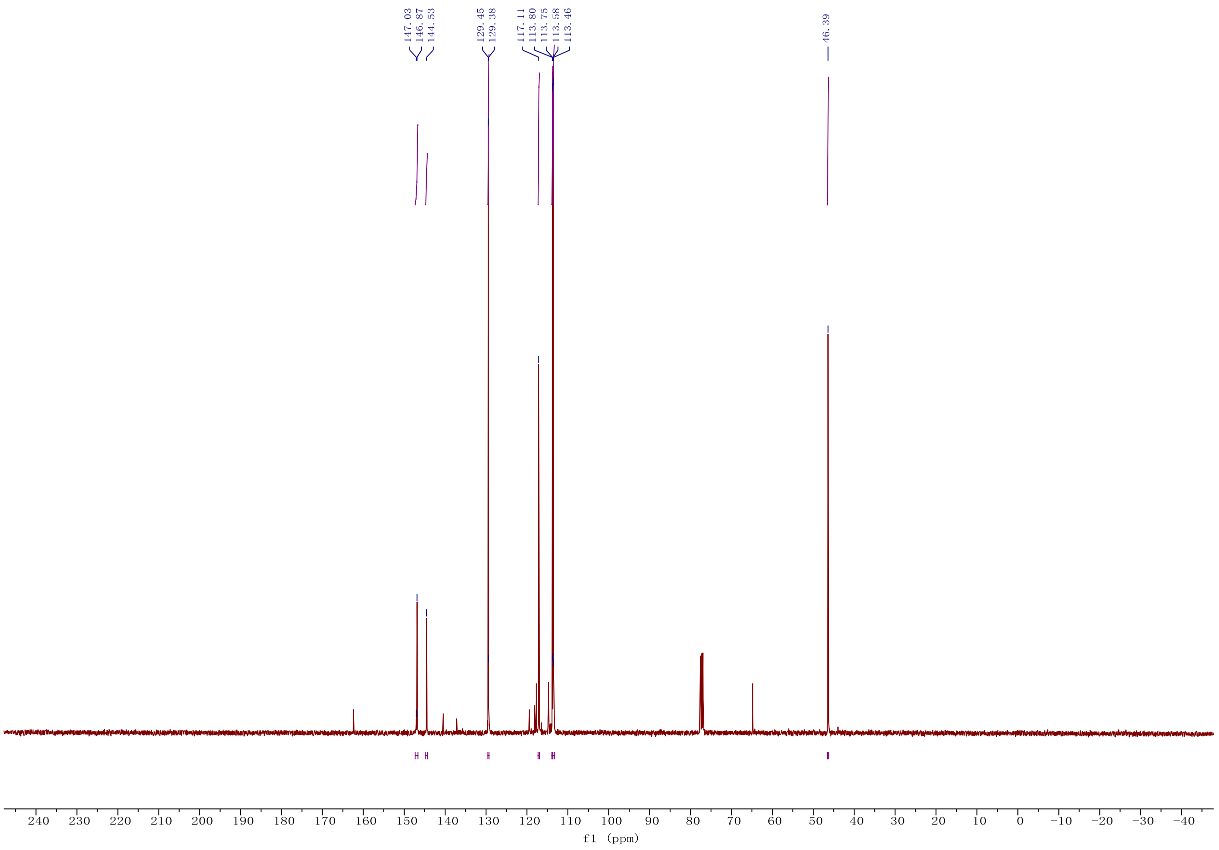

Figure S32 ^{13}C NMR for 3-(aminomethyl)aniline

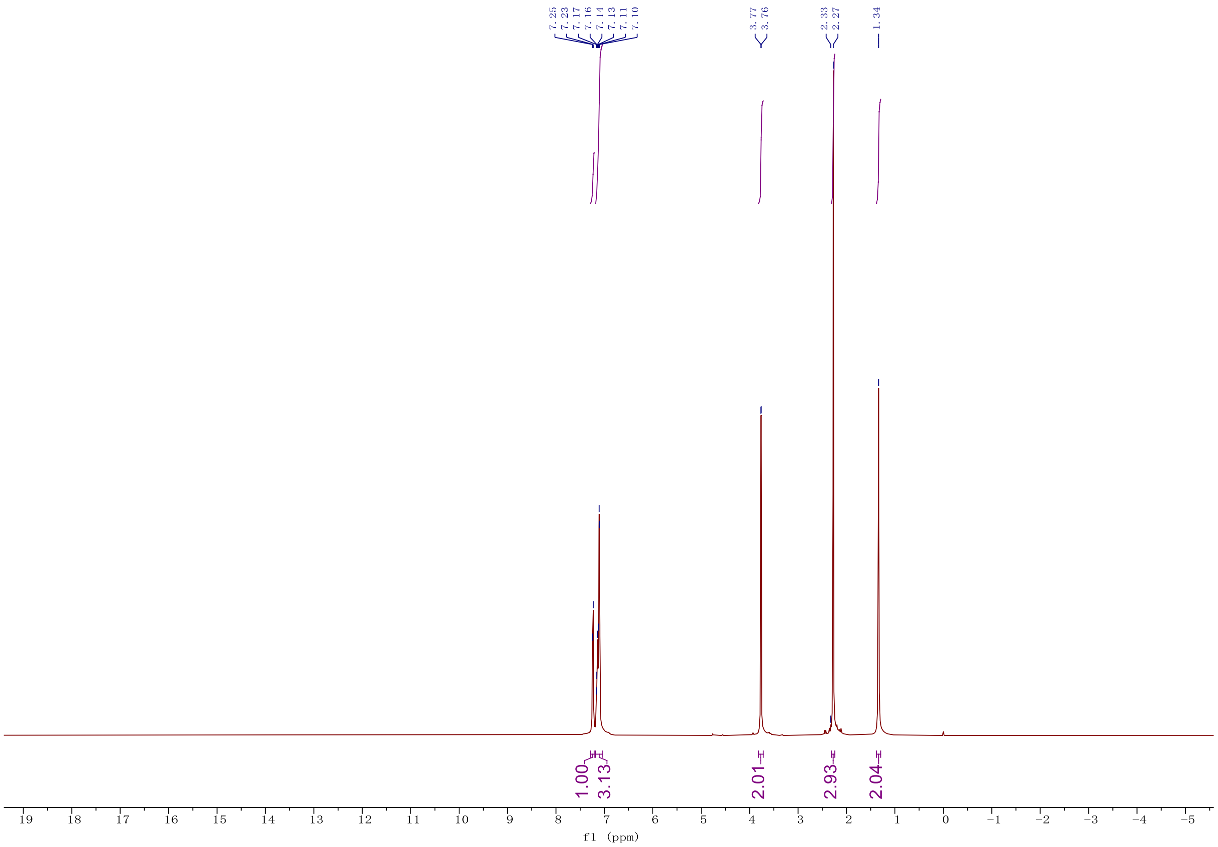

Figure S33 ^{1}H NMR for 2-methylbenzylamine

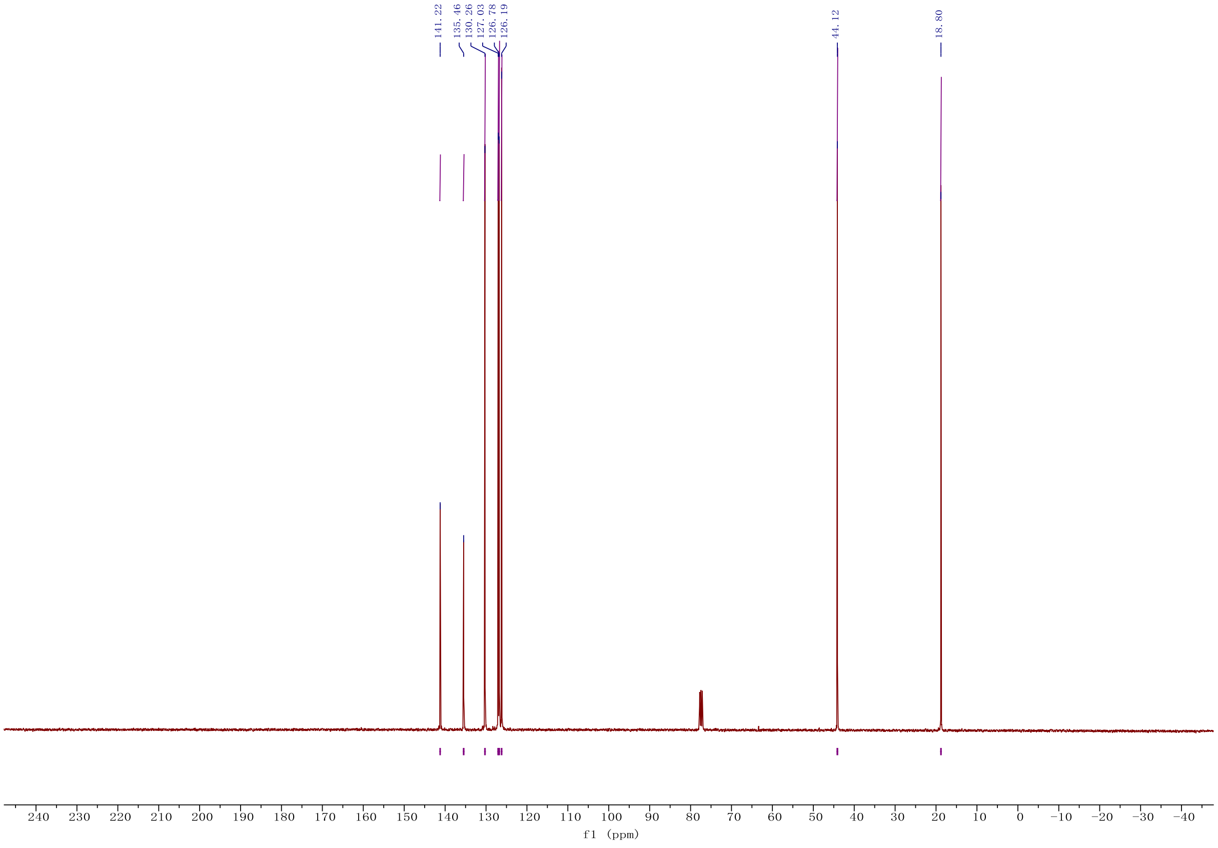

Figure S34 ^{13}C NMR for 2-methylbenzylamine

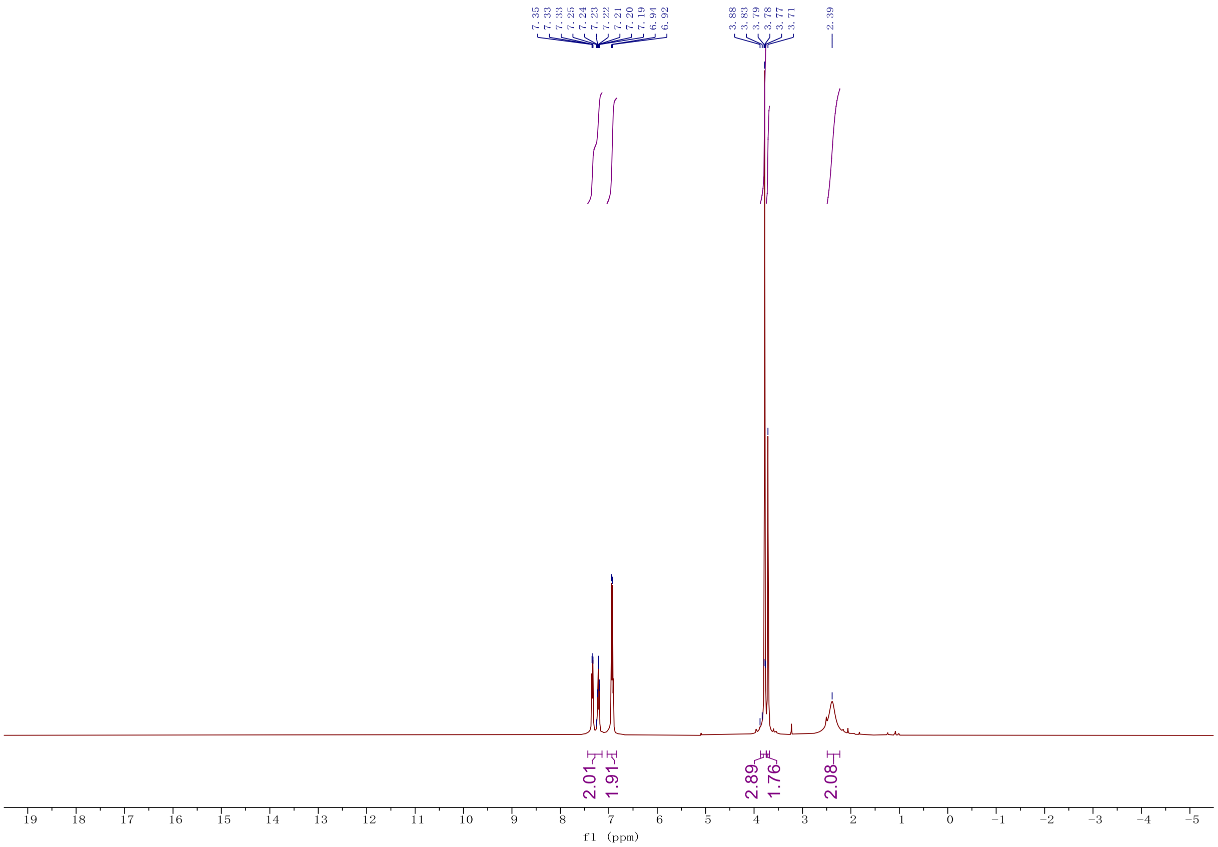

Figure S35 ^{1}H NMR for (2-methoxyphenyl)methanamine

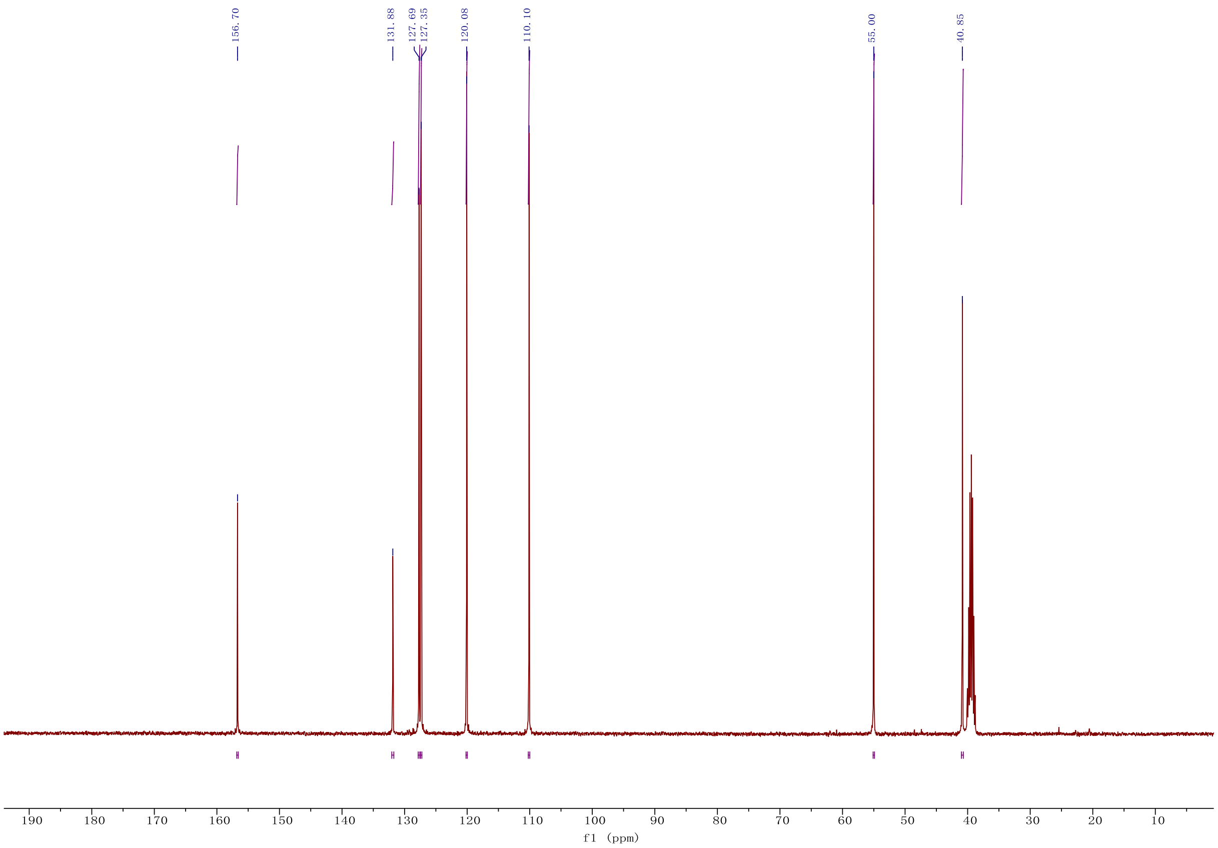

Figure S36 ^{13}C NMR for (2-methoxyphenyl)methanamine

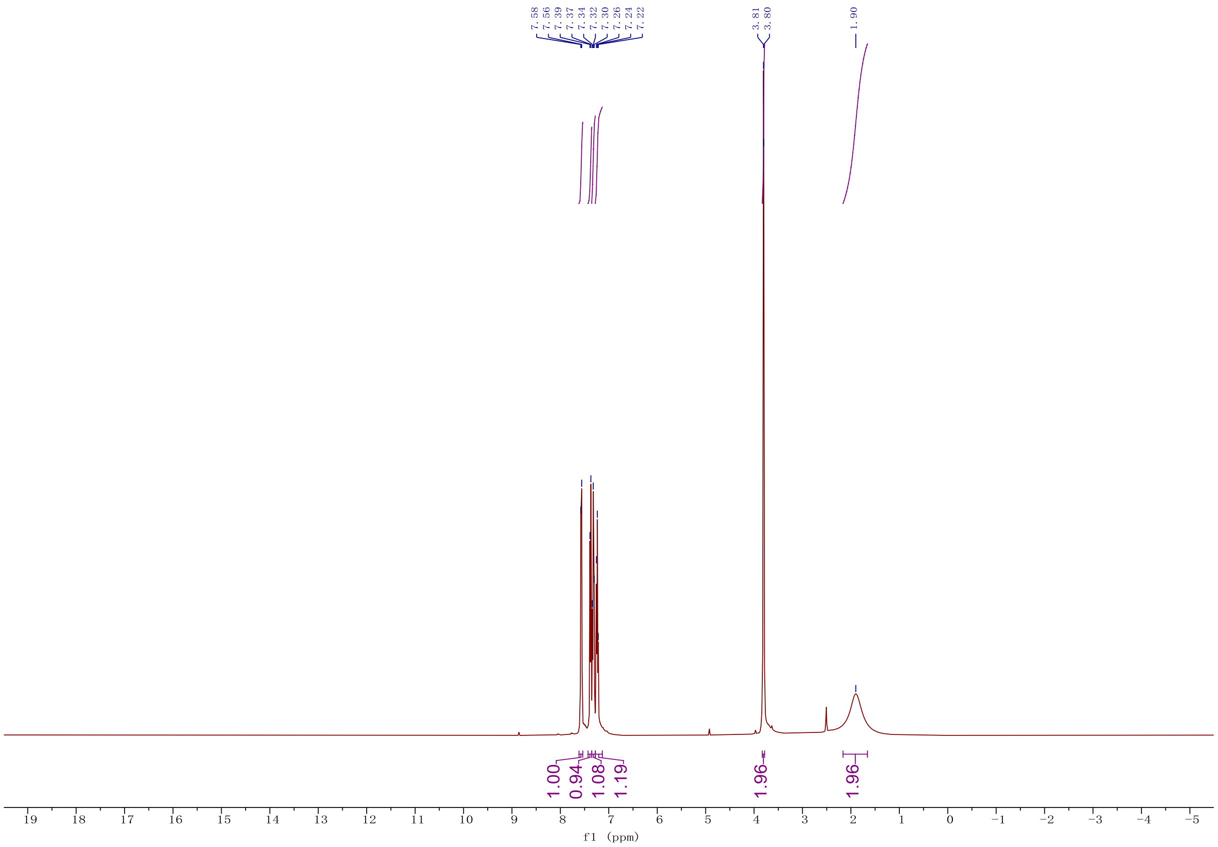

Figure S37 ^{1}H NMR for 2-chlorobenzylamine

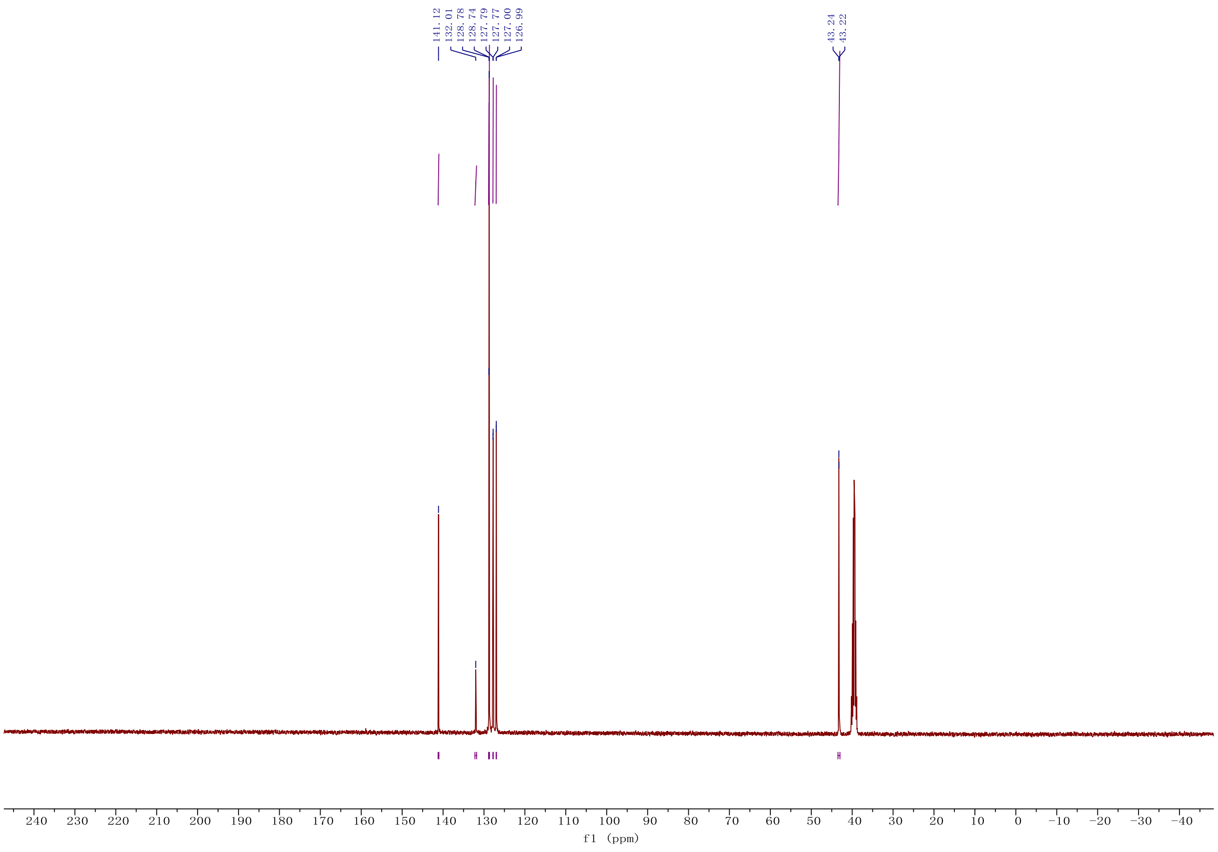

Figure S38 ^{13}C NMR for 2-chlorobenzylamine

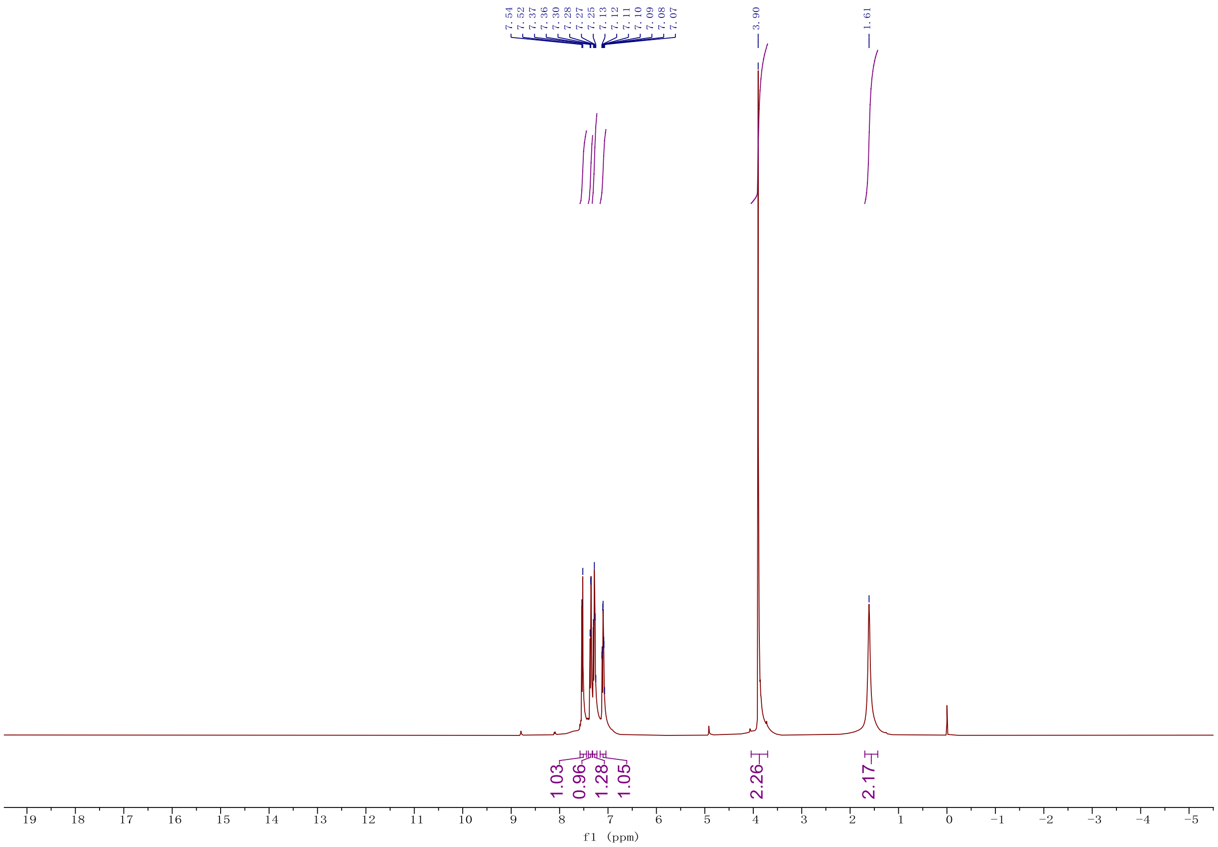

Figure S39 ^{1}H NMR for 2-bromobenzylamine

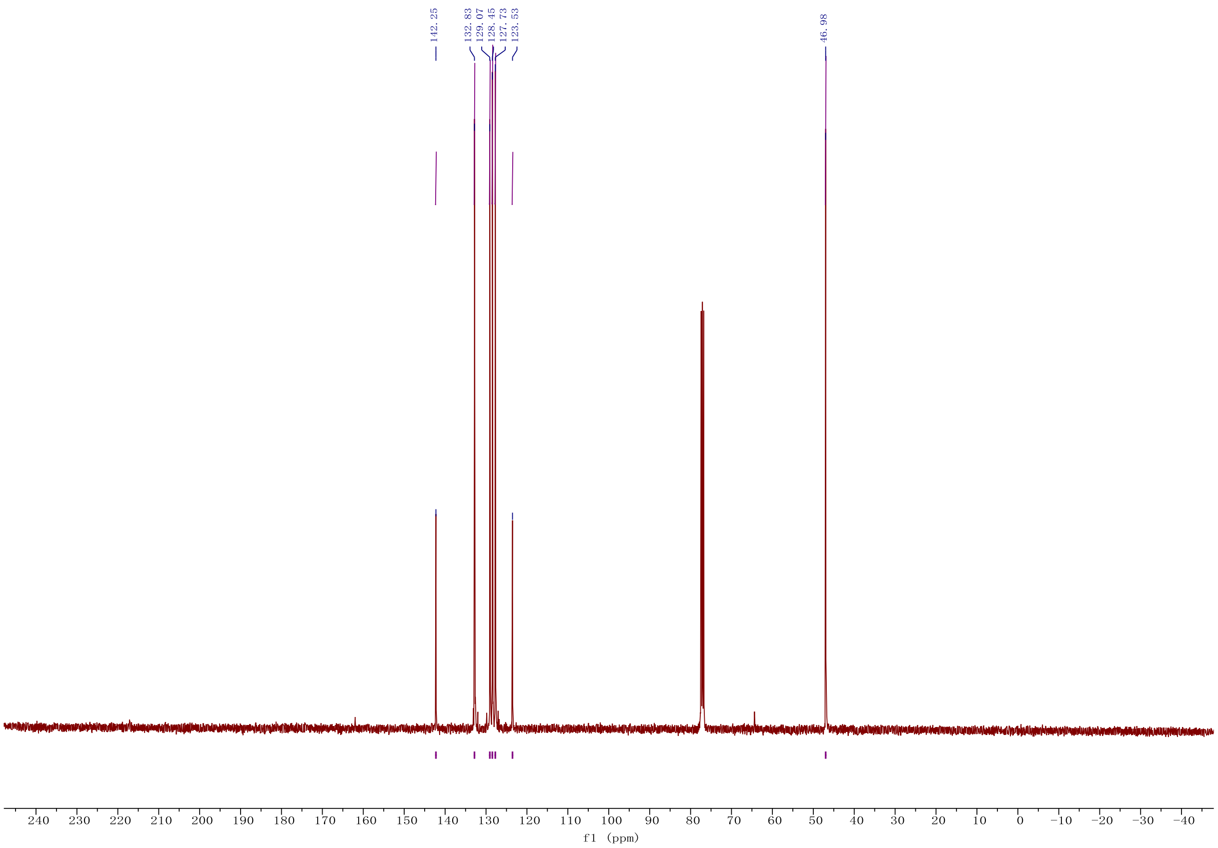

Figure S40 ^{13}C NMR for 2-bromobenzylamine

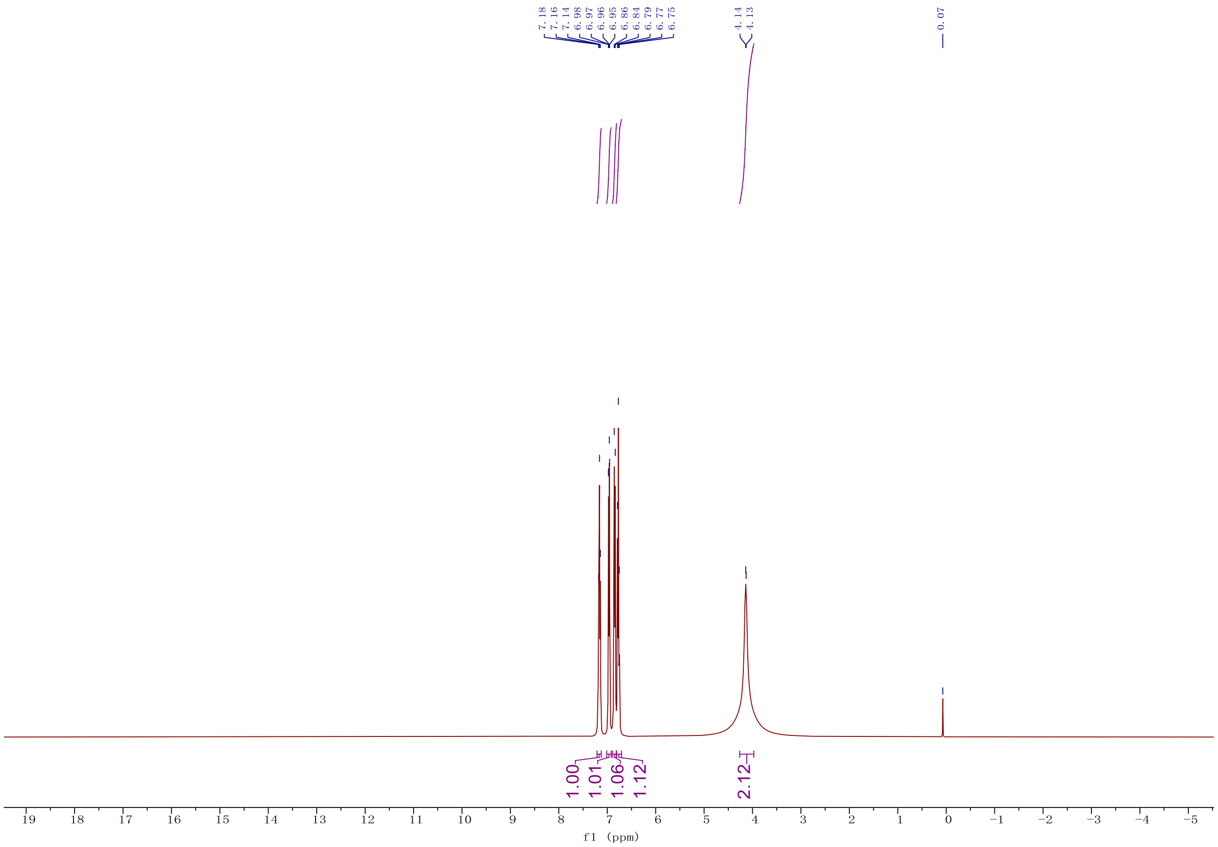

Figure S41 ^{1}H NMR for 2-hydroxybenzylamine

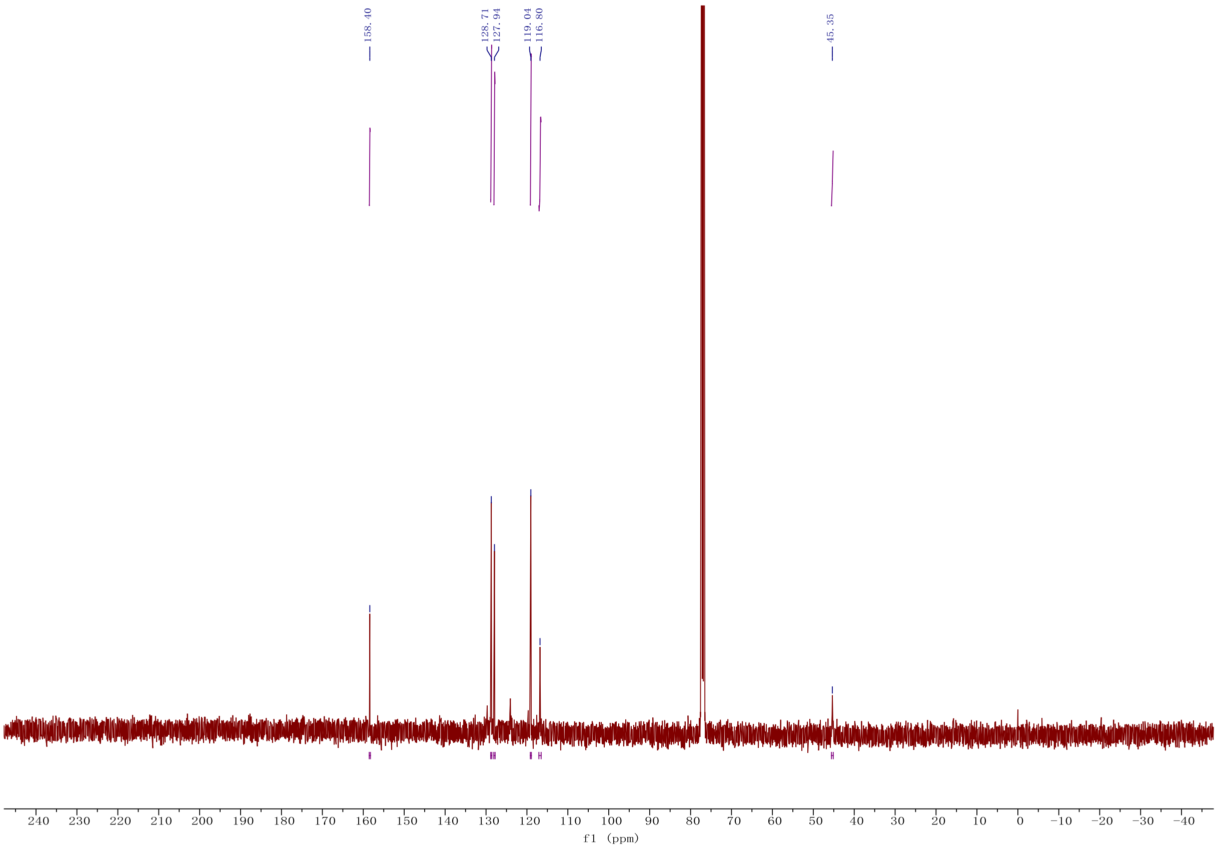

Figure S42 ^{13}C NMR for 2-hydroxybenzylamine

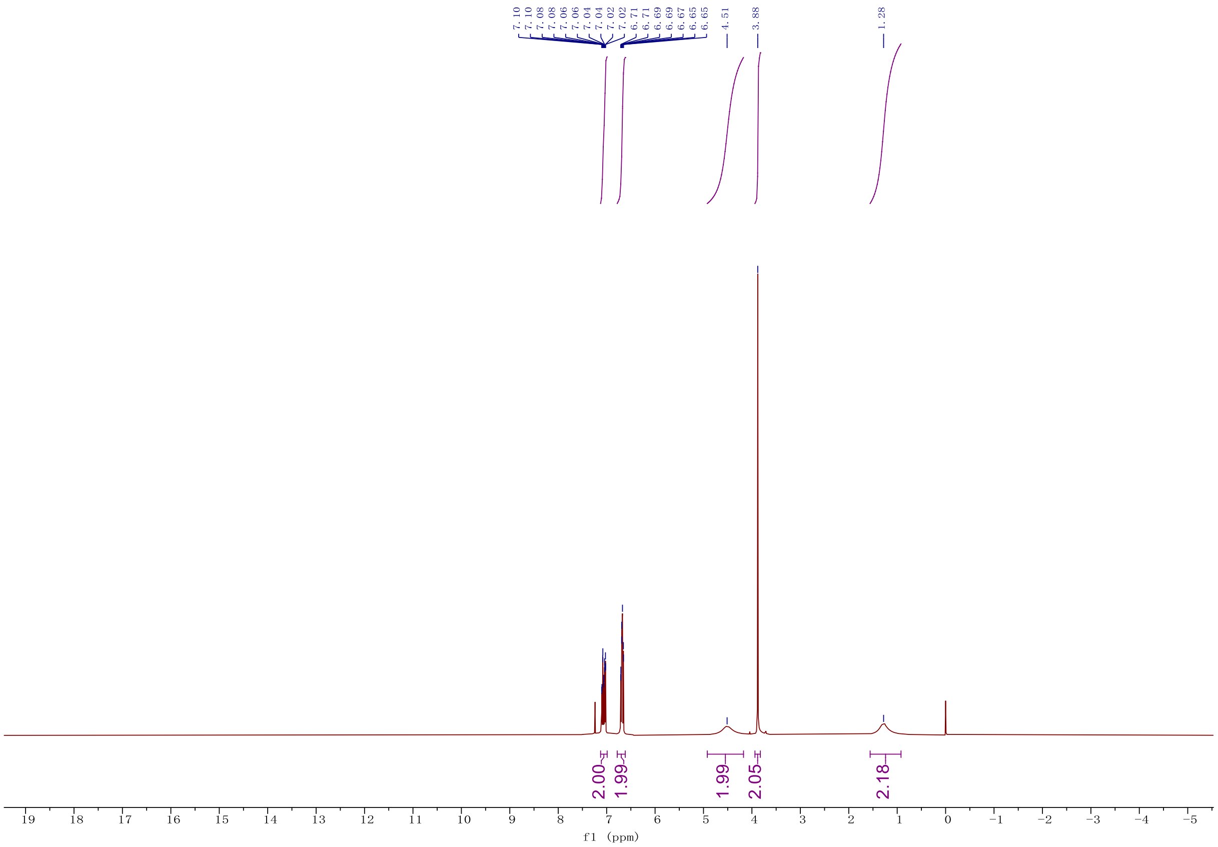

Figure S43 ^{1}H NMR for 2-(aminomethyl)aniline

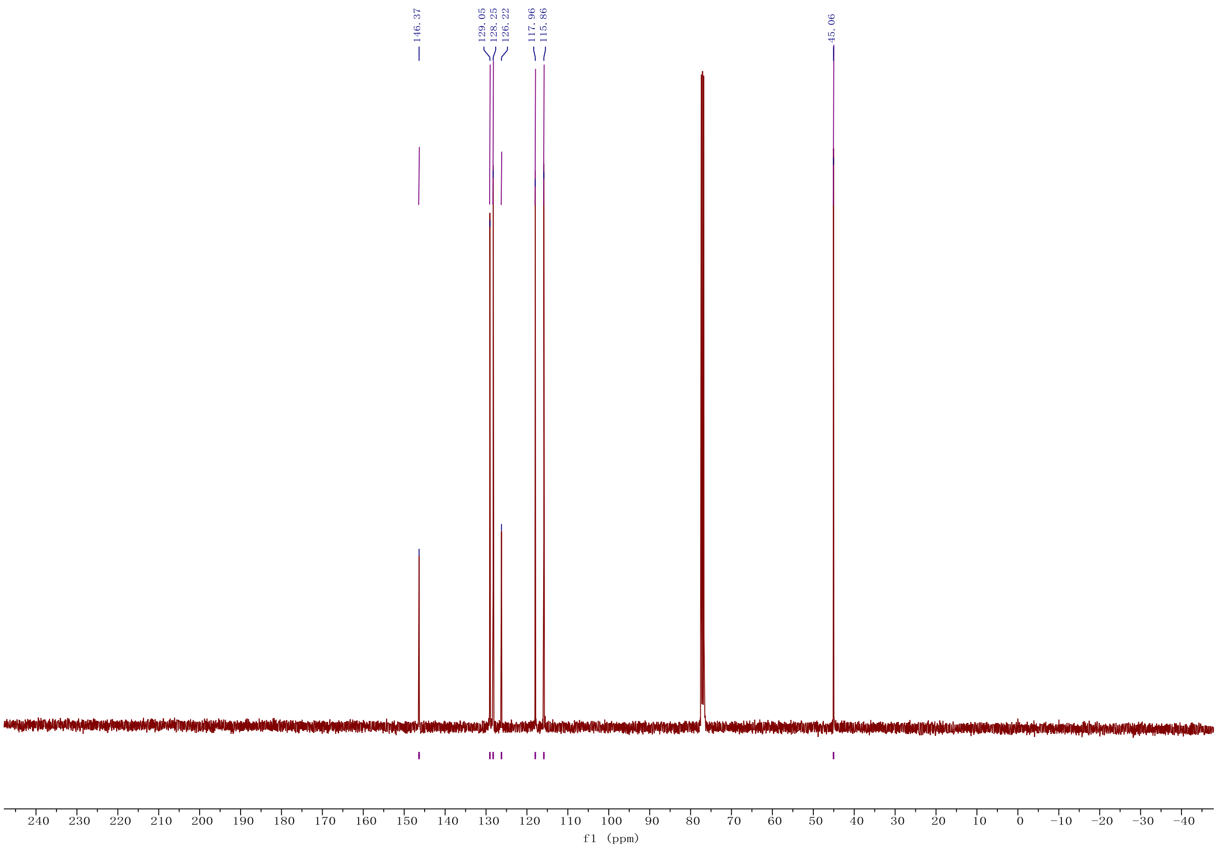

Figure S44 ^{13}C NMR for 2-(aminomethyl)aniline

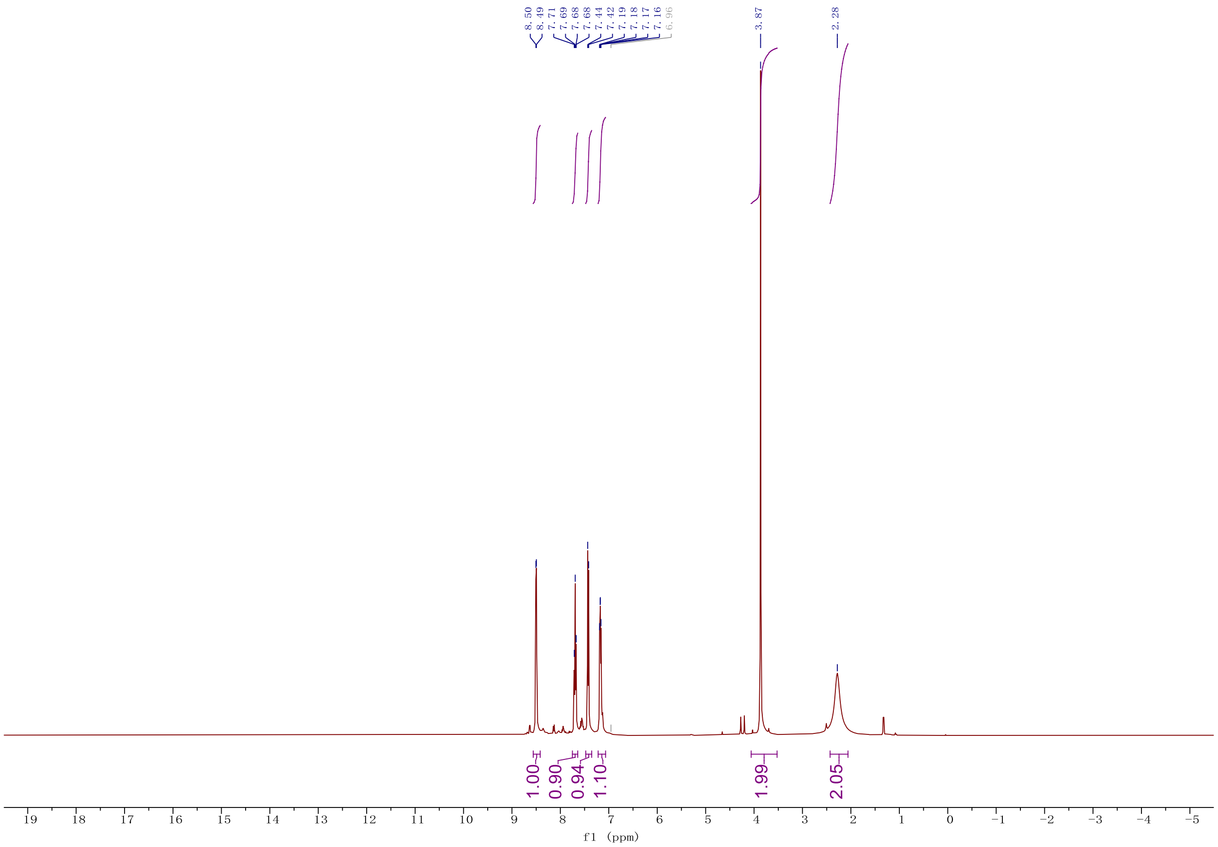

Figure S45 ^{1}H NMR for 2-picolinamine

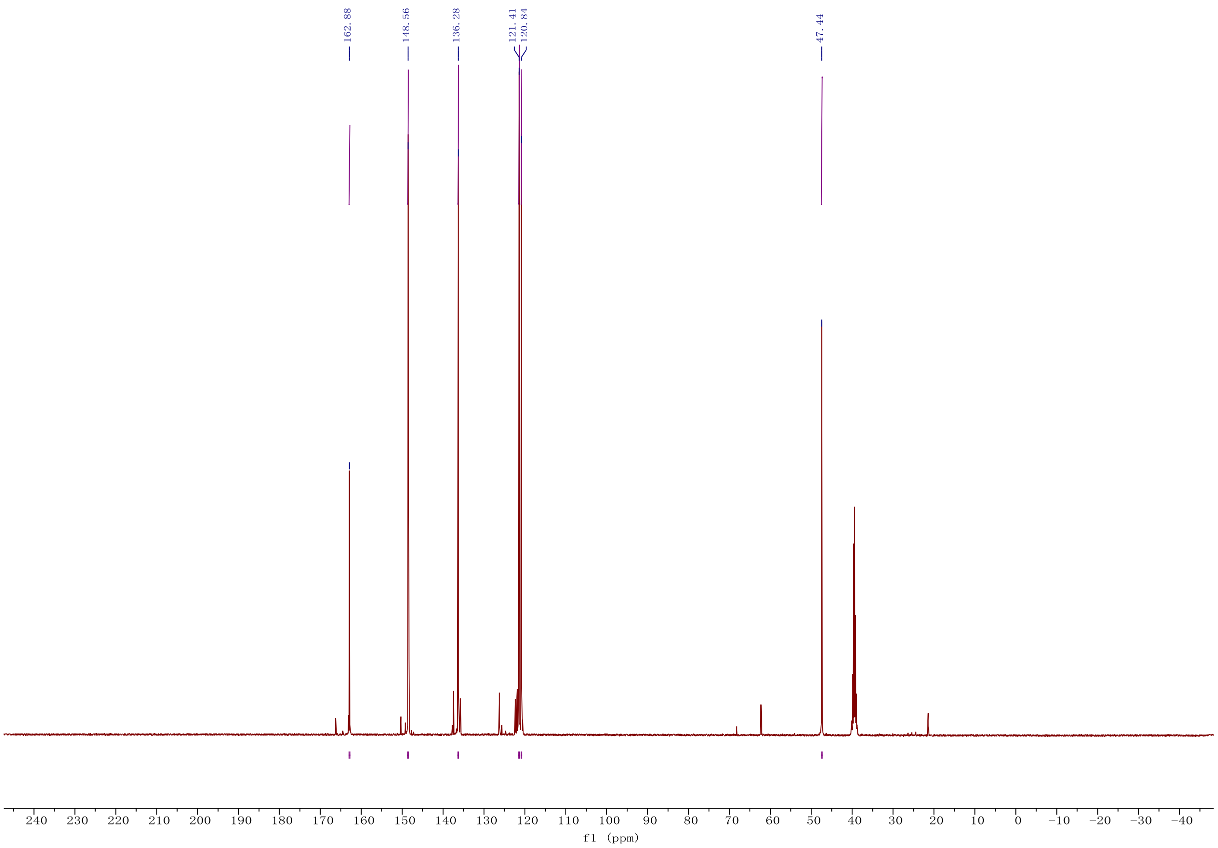

Figure S46 ^{13}C NMR for 2-picolinamine

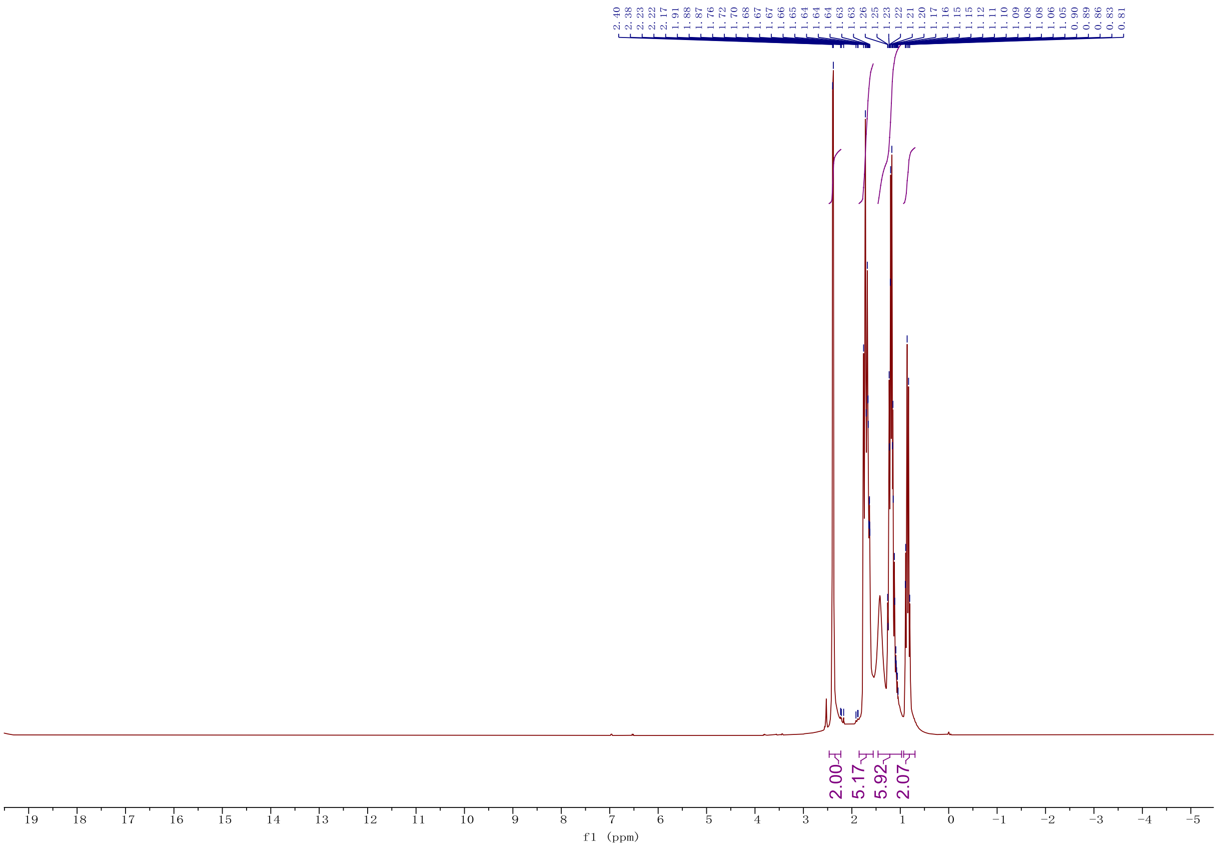

Figure S47 ^{1}H NMR for cyclohexylmethylamine

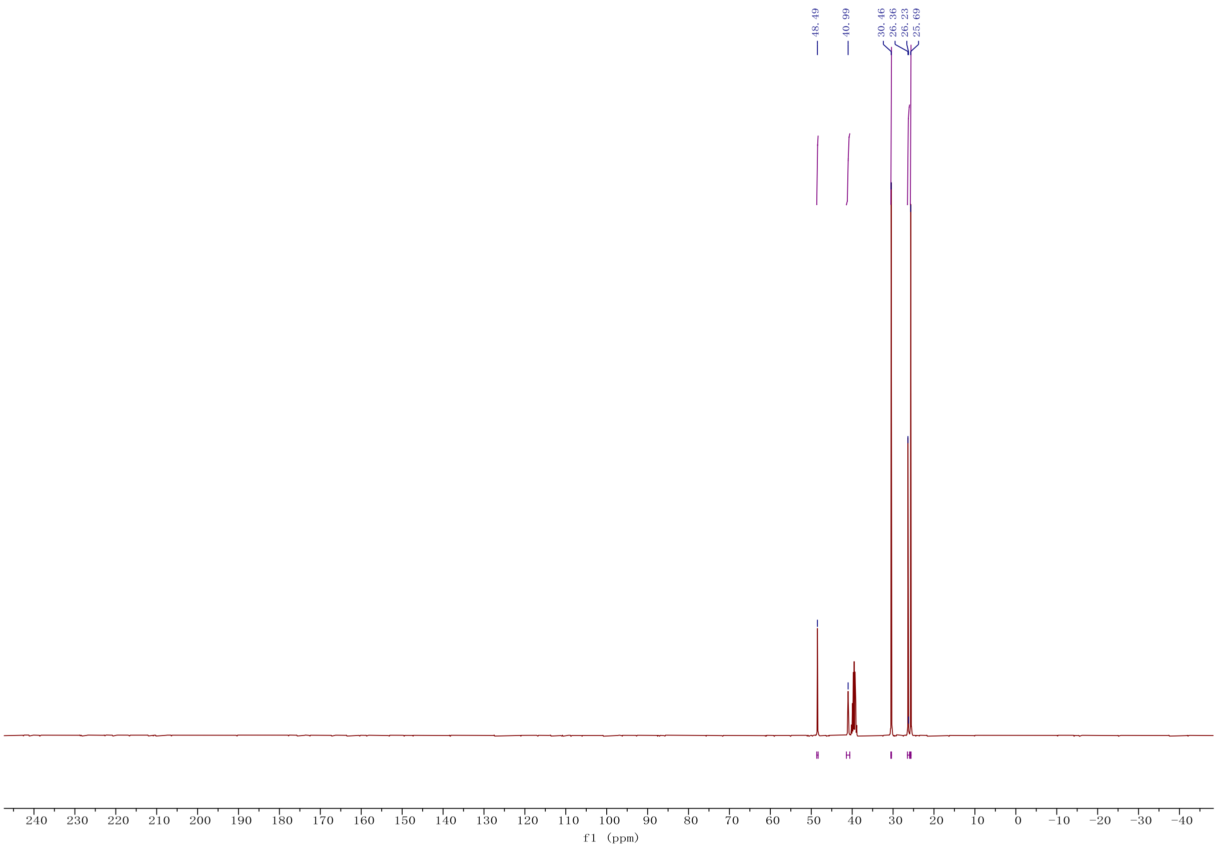

Figure S48 ^{13}C NMR for cyclohexylmethylamine

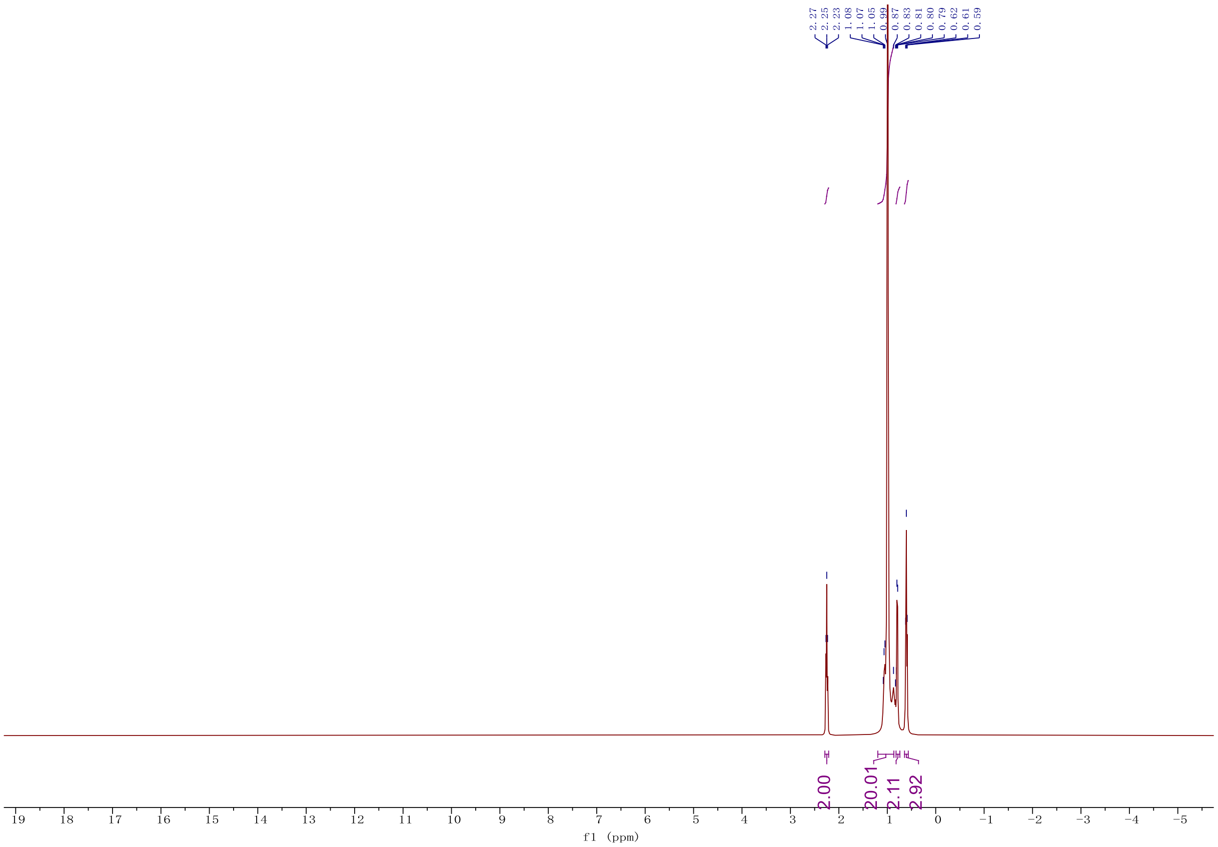

Figure S49 ^{1}H NMR for dodecylamine

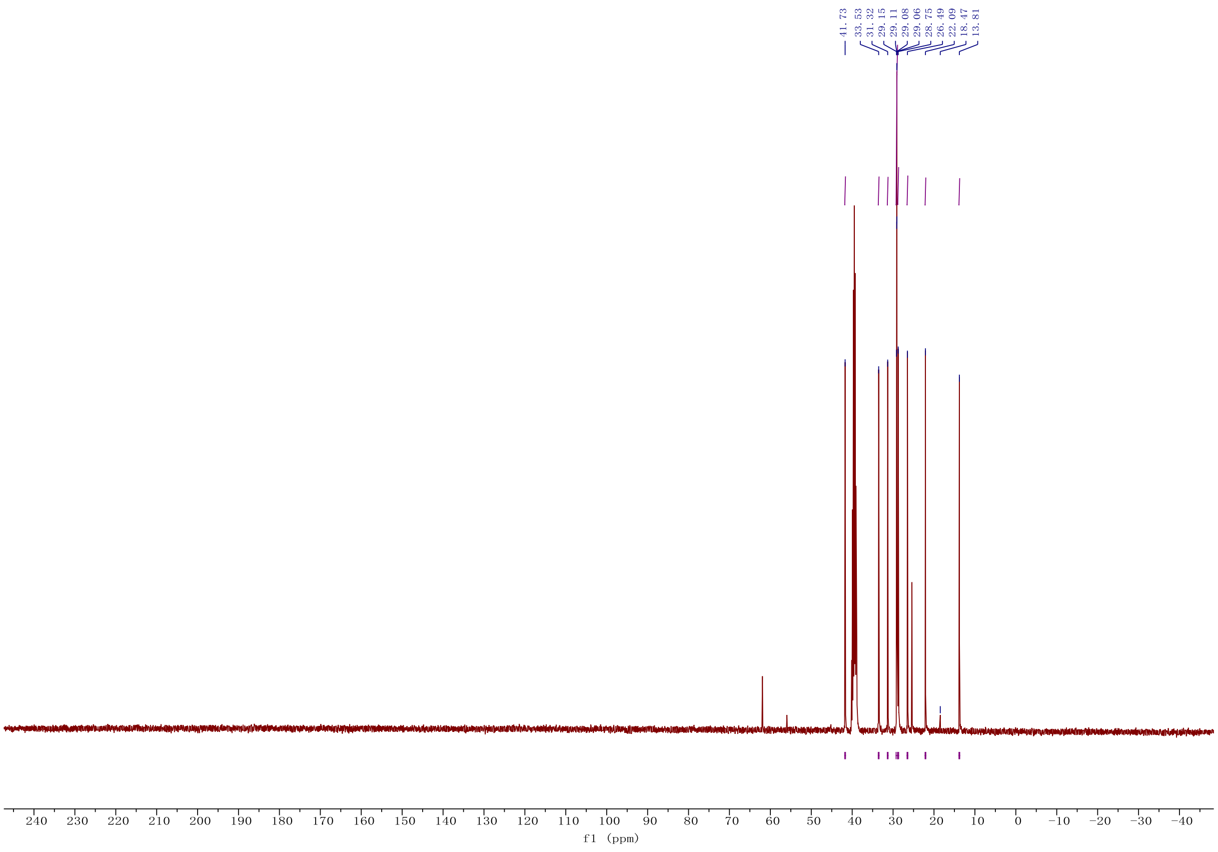

Figure S50 ^{13}C NMR for dodecylamine

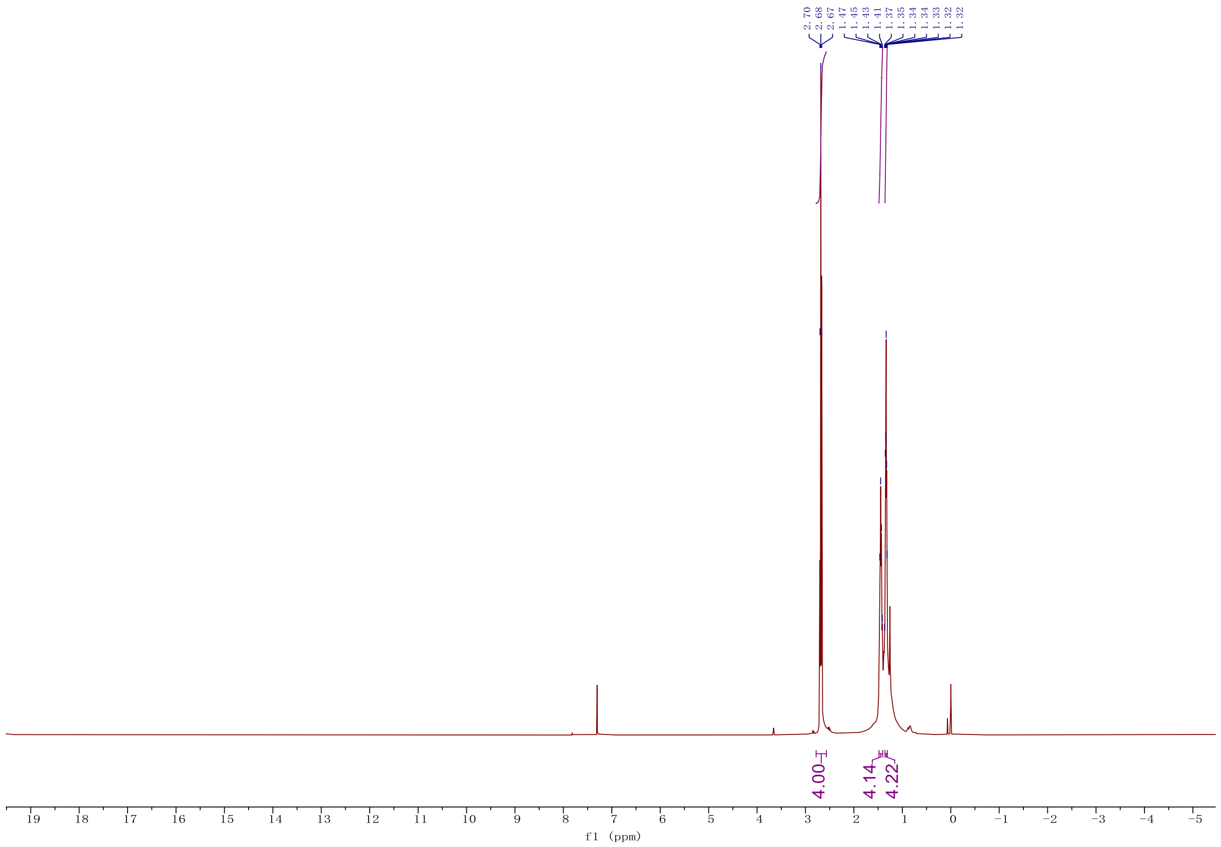

Figure S51 ^{1}H NMR for 1,6-hexanediamine

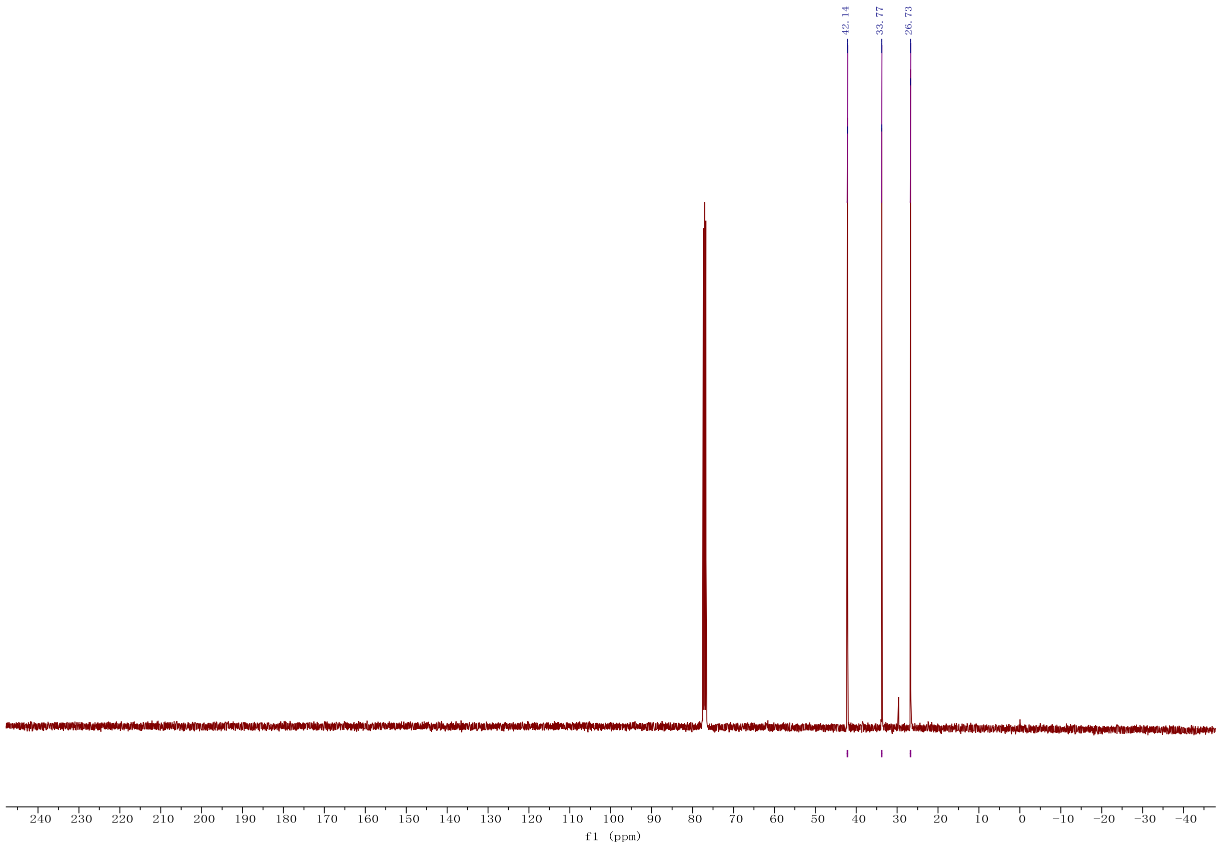

Figure S52 ^{13}C NMR for 1,6-hexanediamine

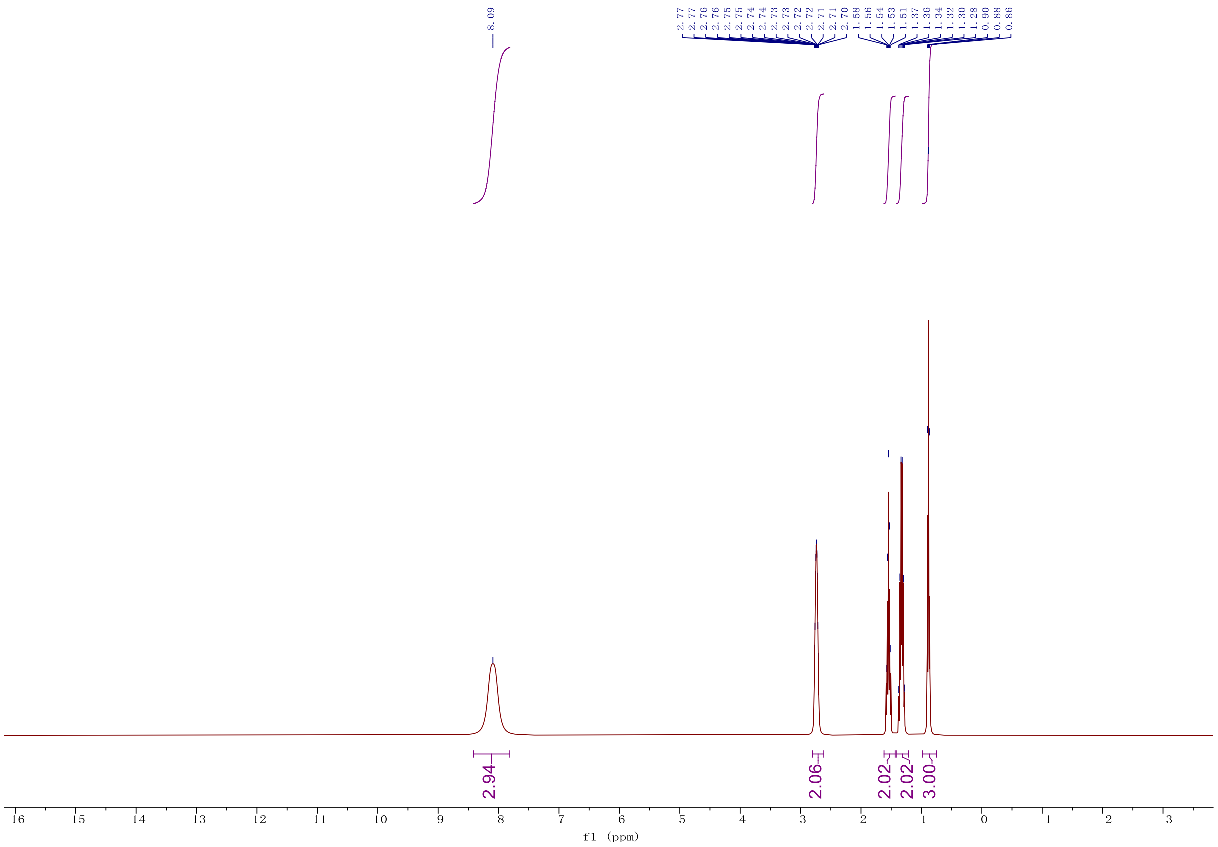

Figure S53 ^{1}H NMR for n-butylamine hydrochloride

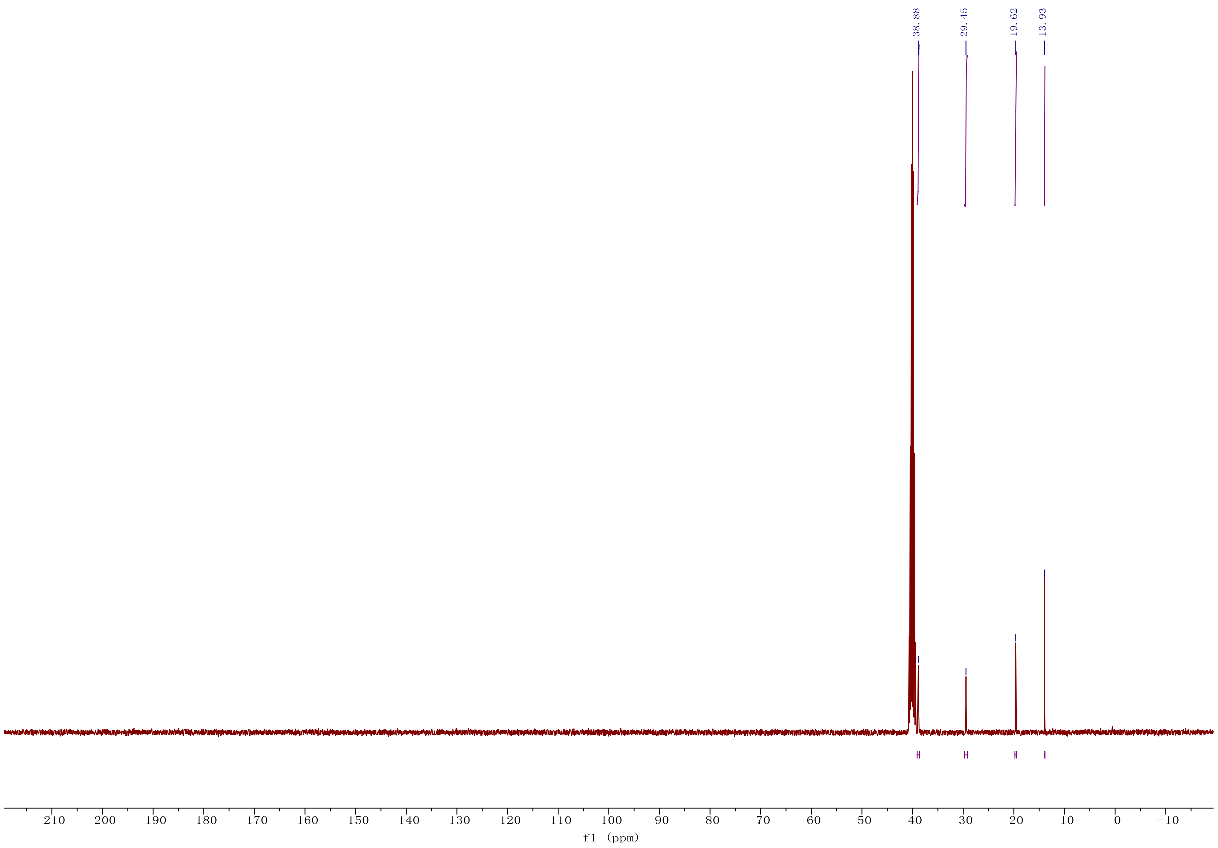

Figure S54 ^{13}C NMR for n-butylamine hydrochloride

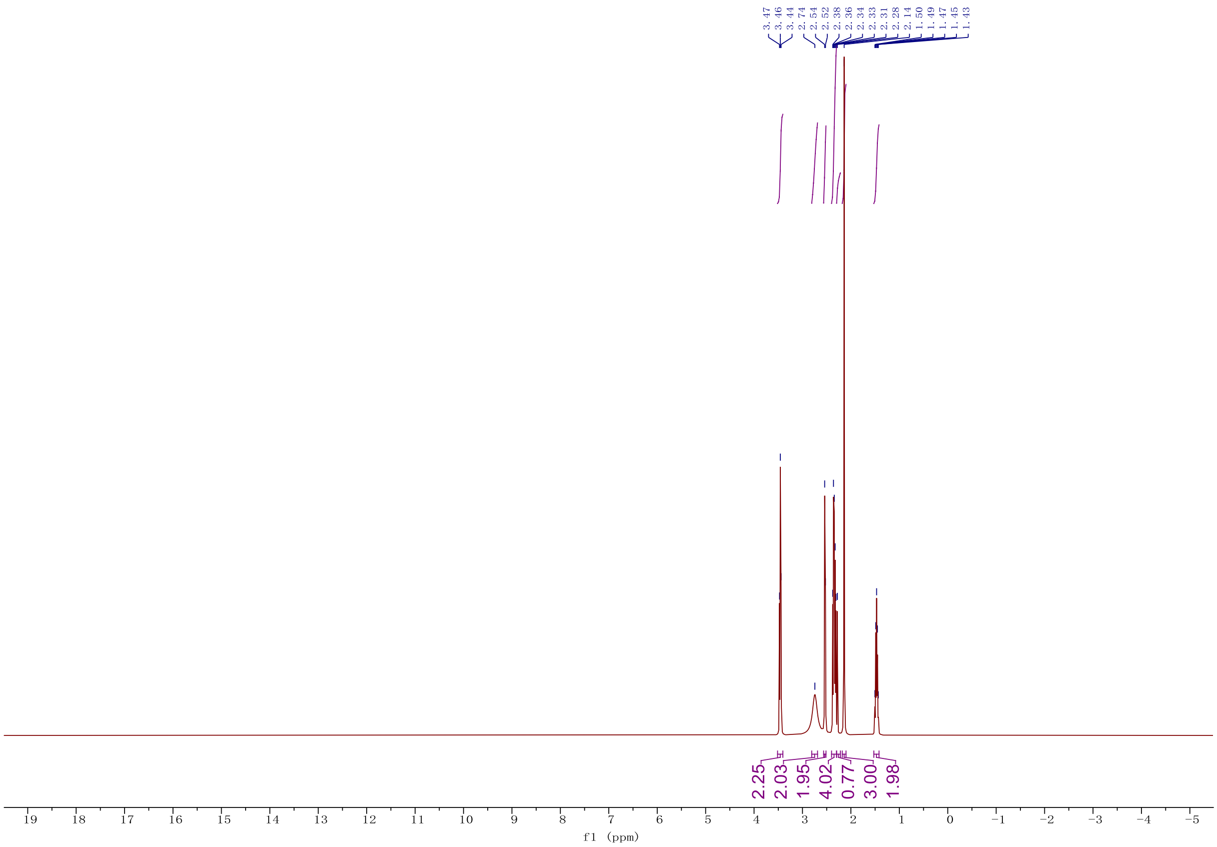

Figure S55 ^{1}H NMR for (3-aminopropyl)(2-hydroxyethyl)methylamine

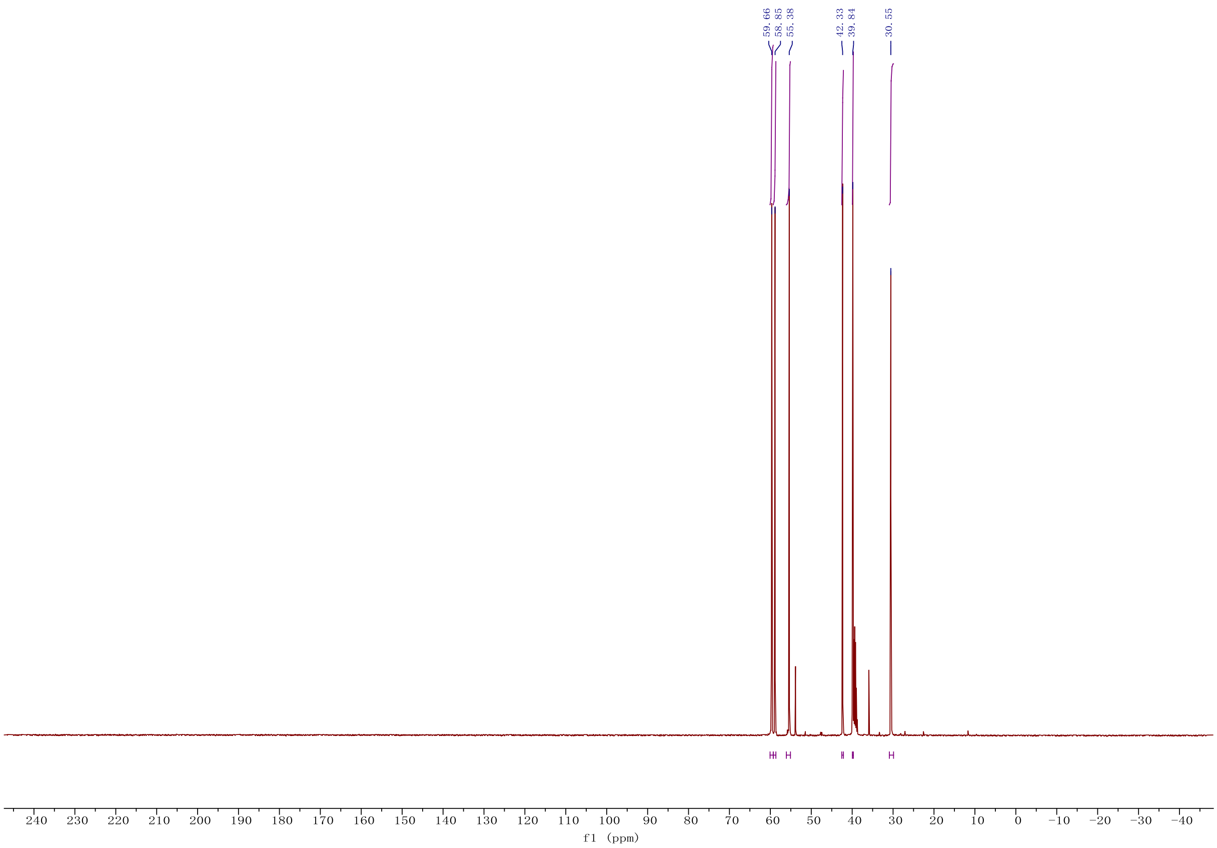

Figure S56 ^{13}C NMR for (3-aminopropyl)(2-hydroxyethyl)methylamine

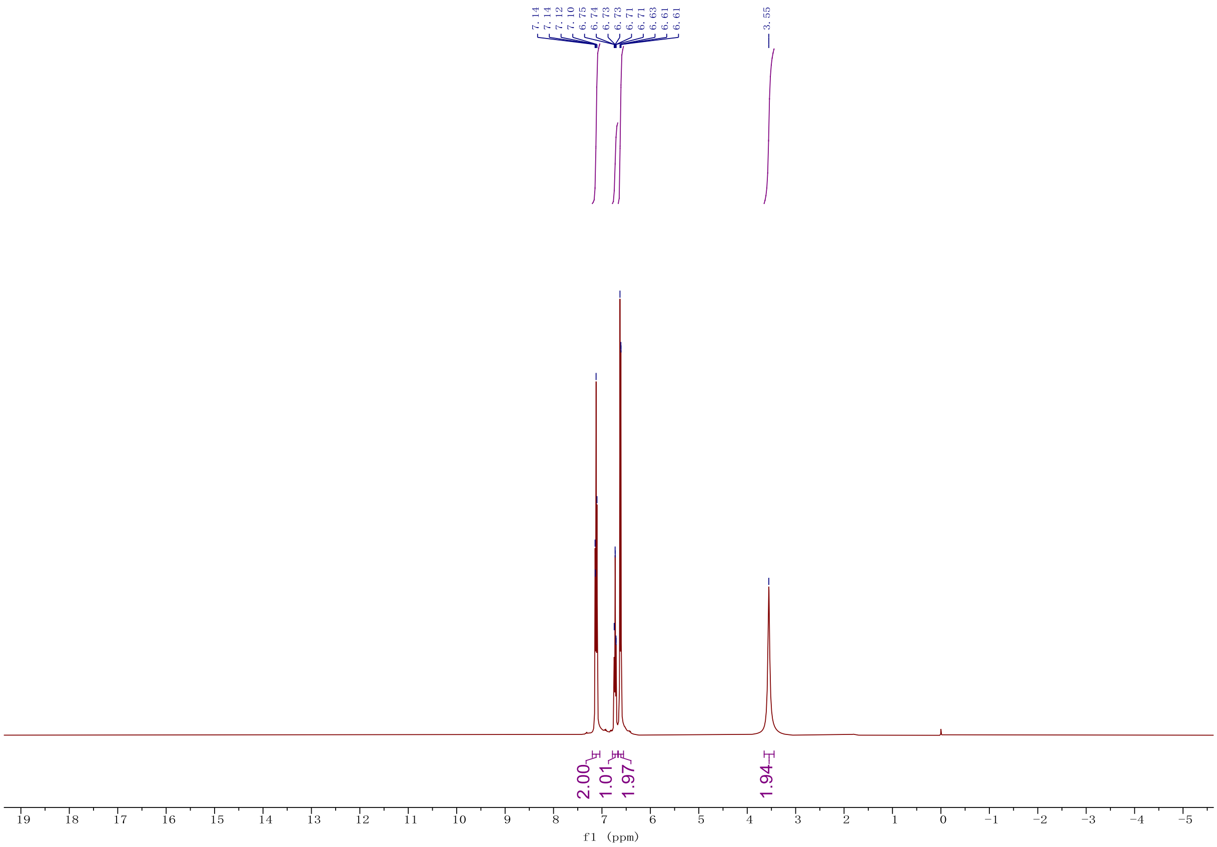

Figure S57 ^{1}H NMR for aniline

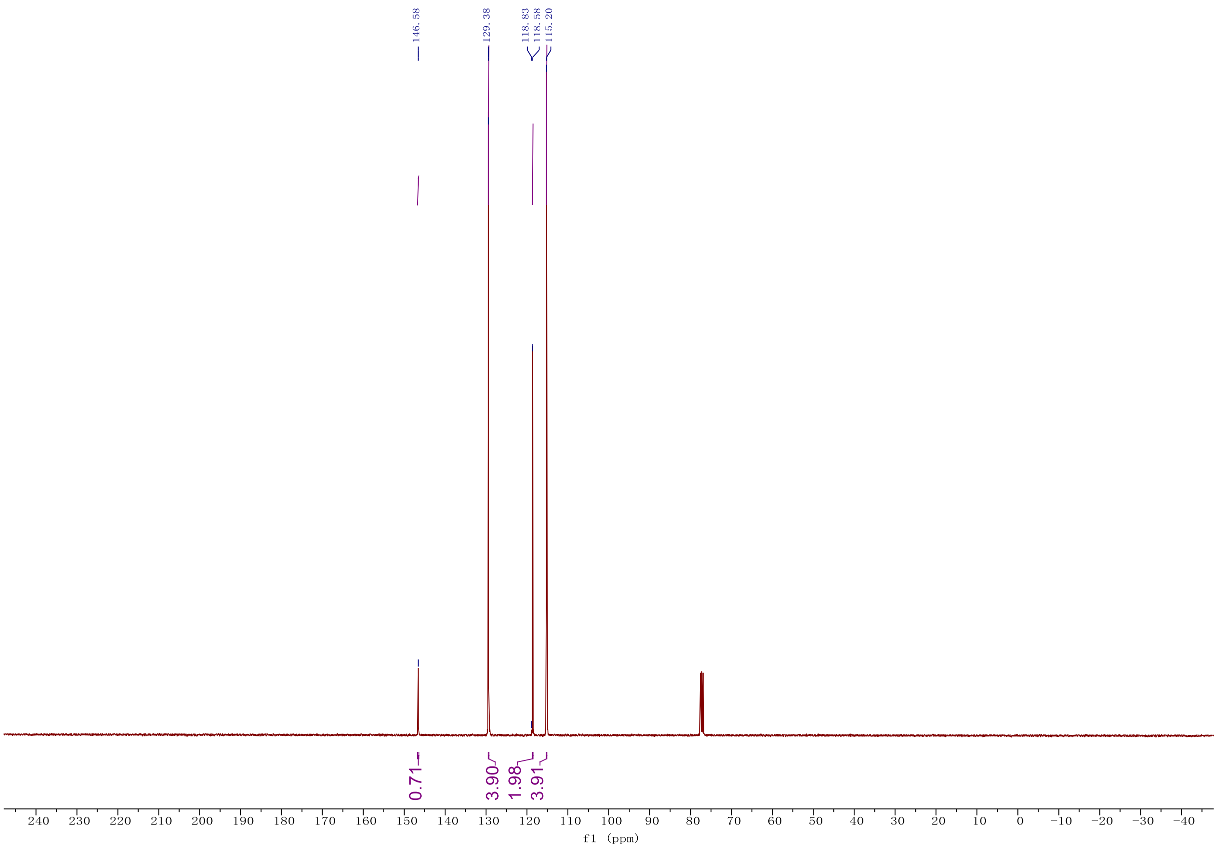

Figure S58 ^{13}C NMR for aniline

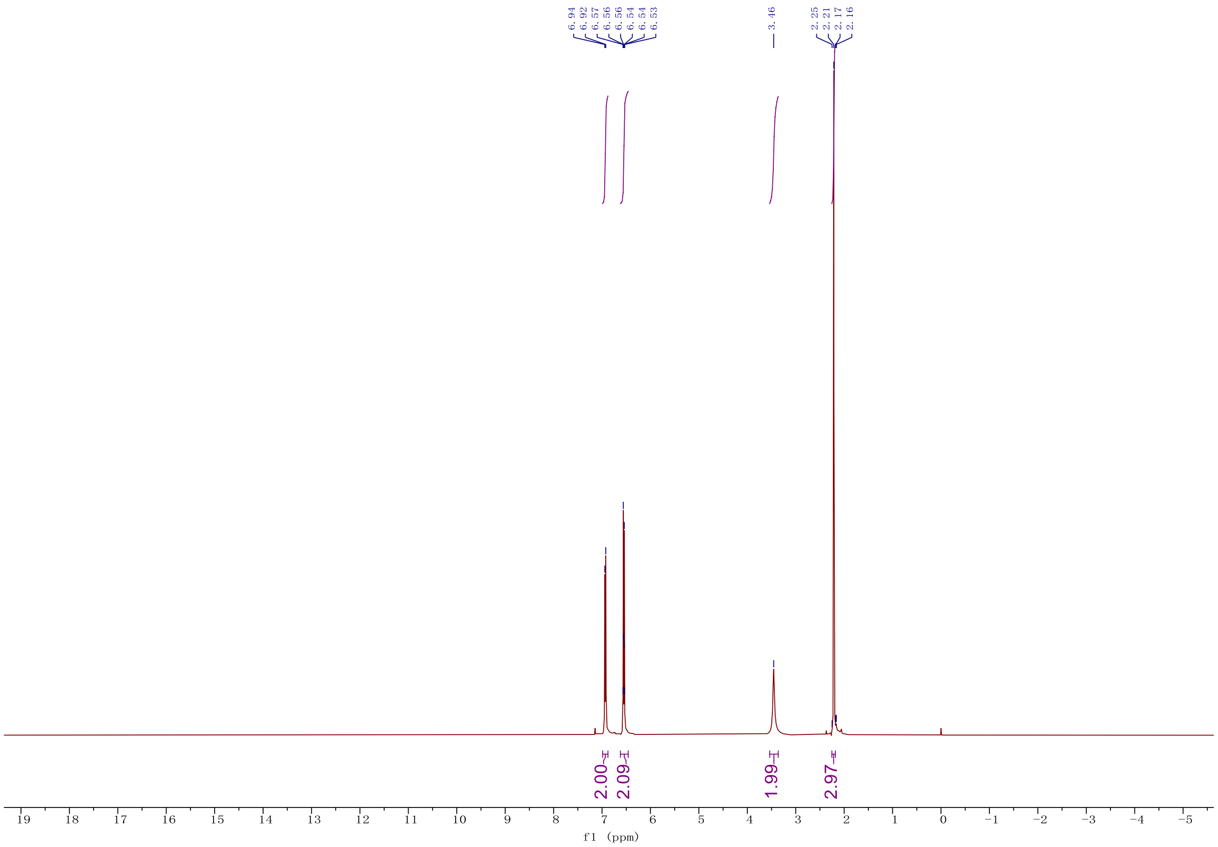

Figure S59 ^{1}H NMR for p-toluidine

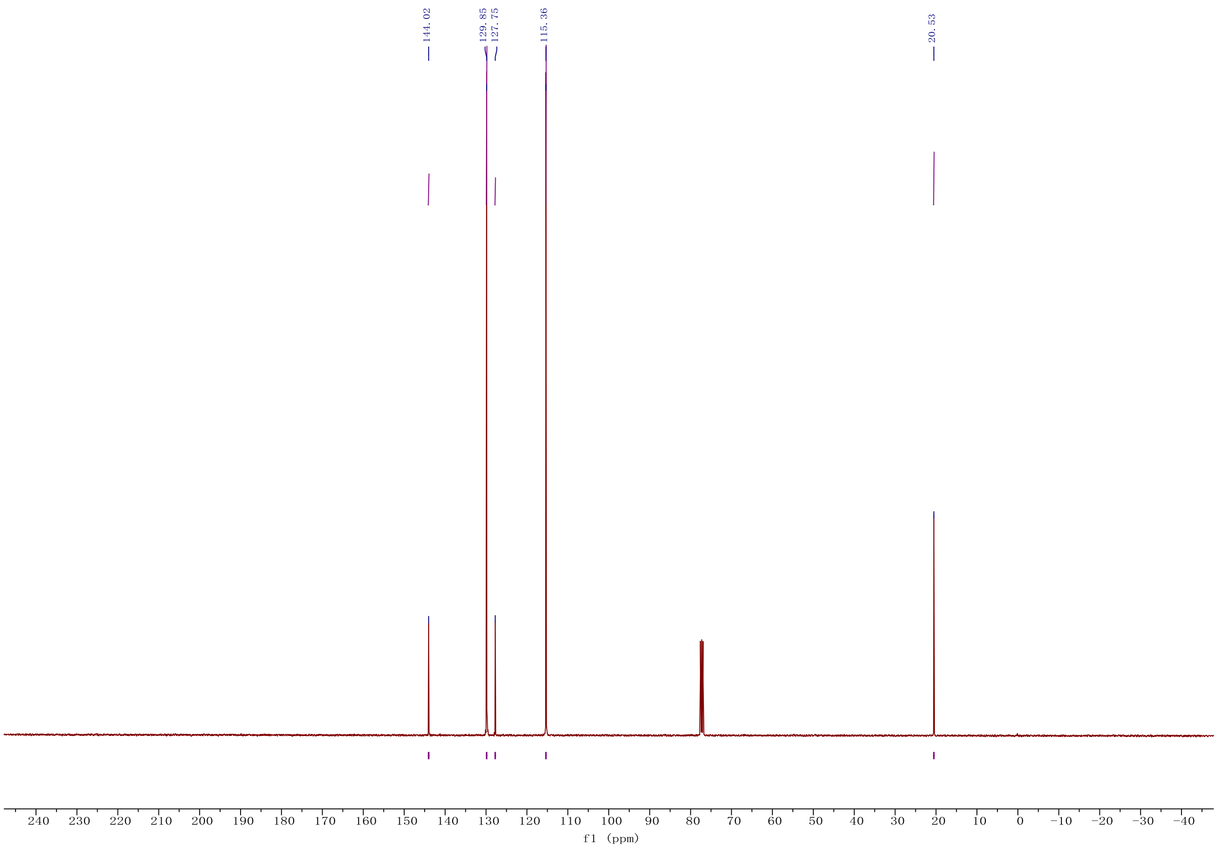

Figure S60 ^{13}C NMR for p-toluidine

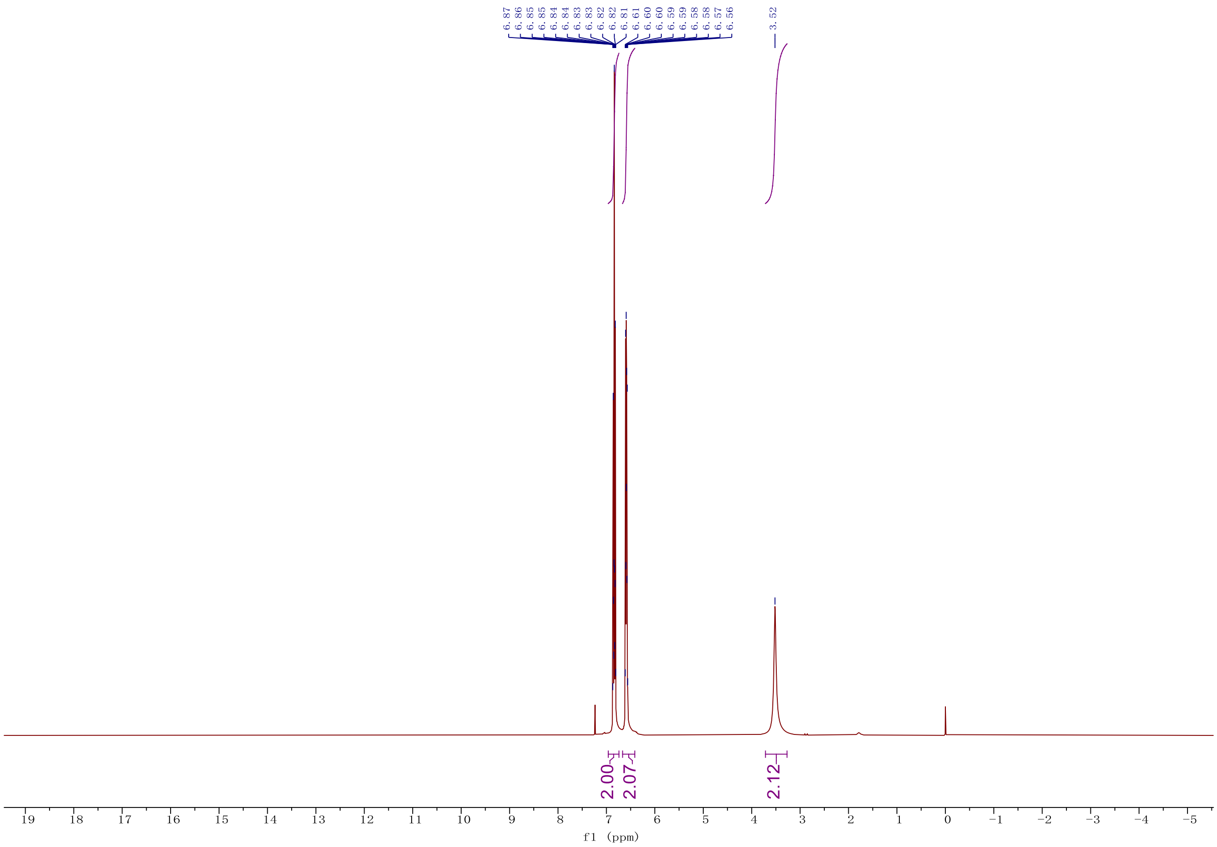

Figure S61 ^{1}H NMR for 4-fluoroaniline

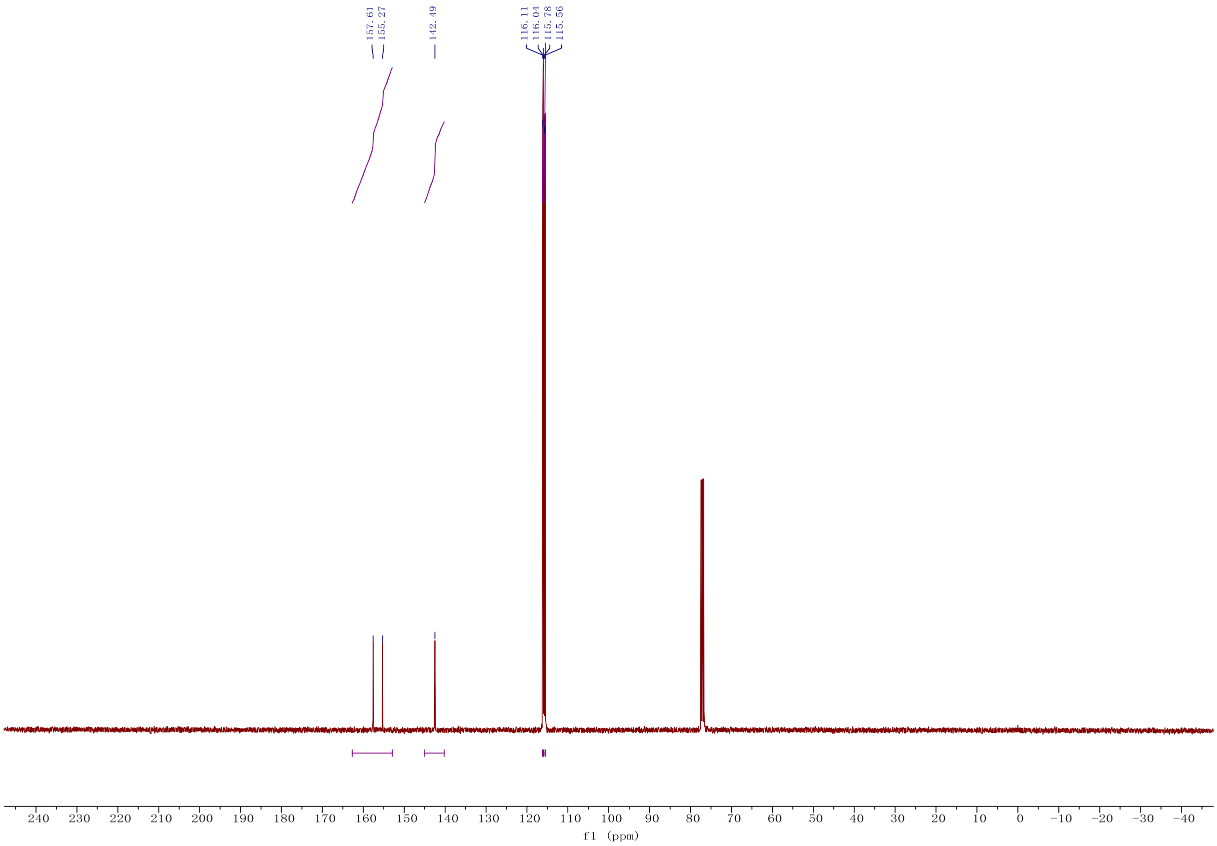

Figure S62 ^{13}C NMR for 4-fluoroaniline

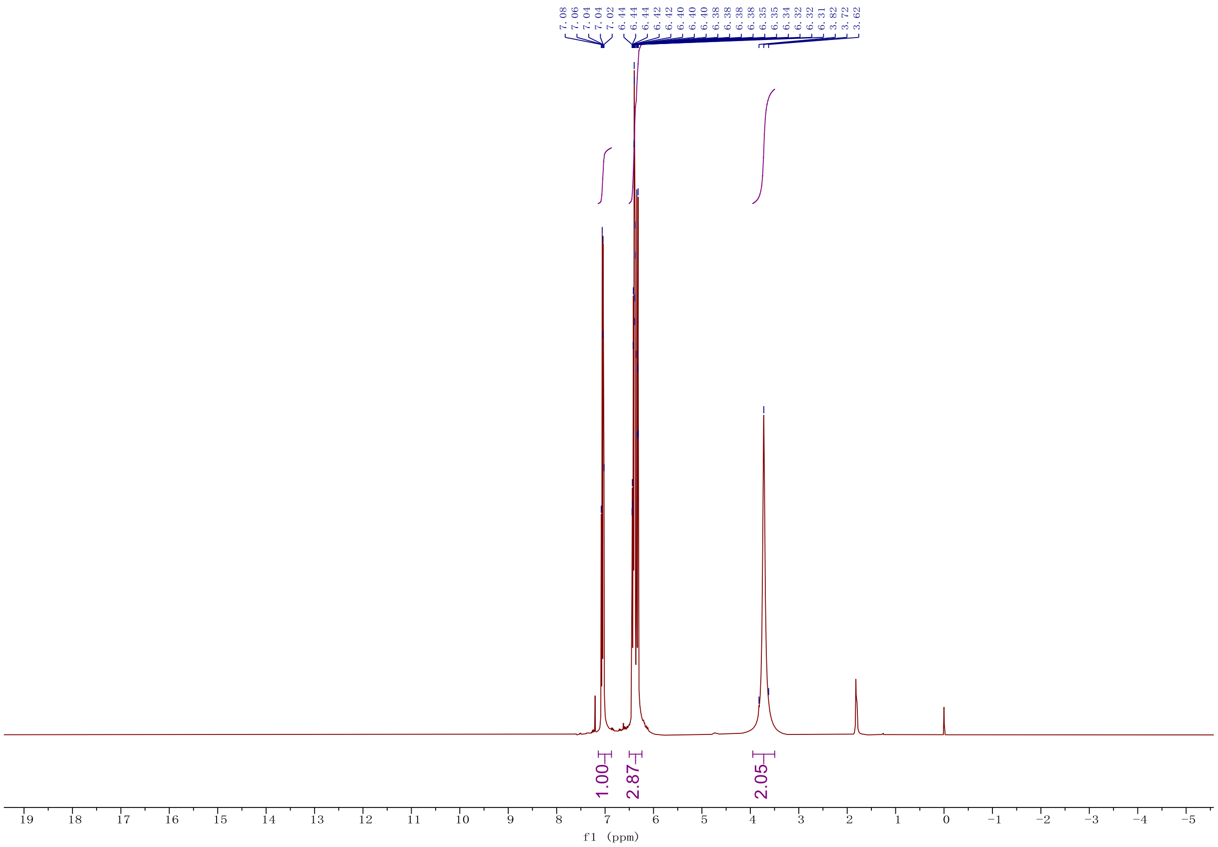

Figure S63 ^{1}H NMR for 3-fluoroaniline

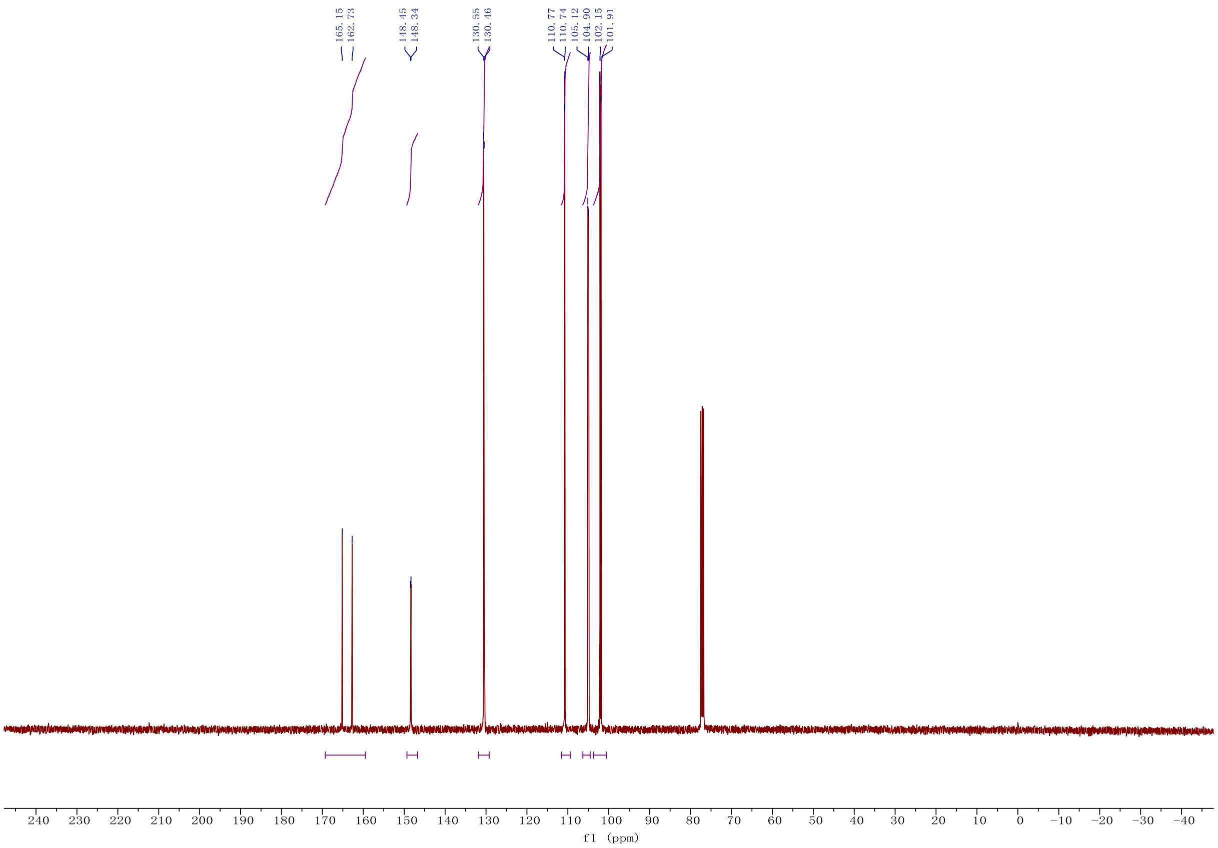

Figure S64 ^{13}C NMR for 3-fluoroaniline

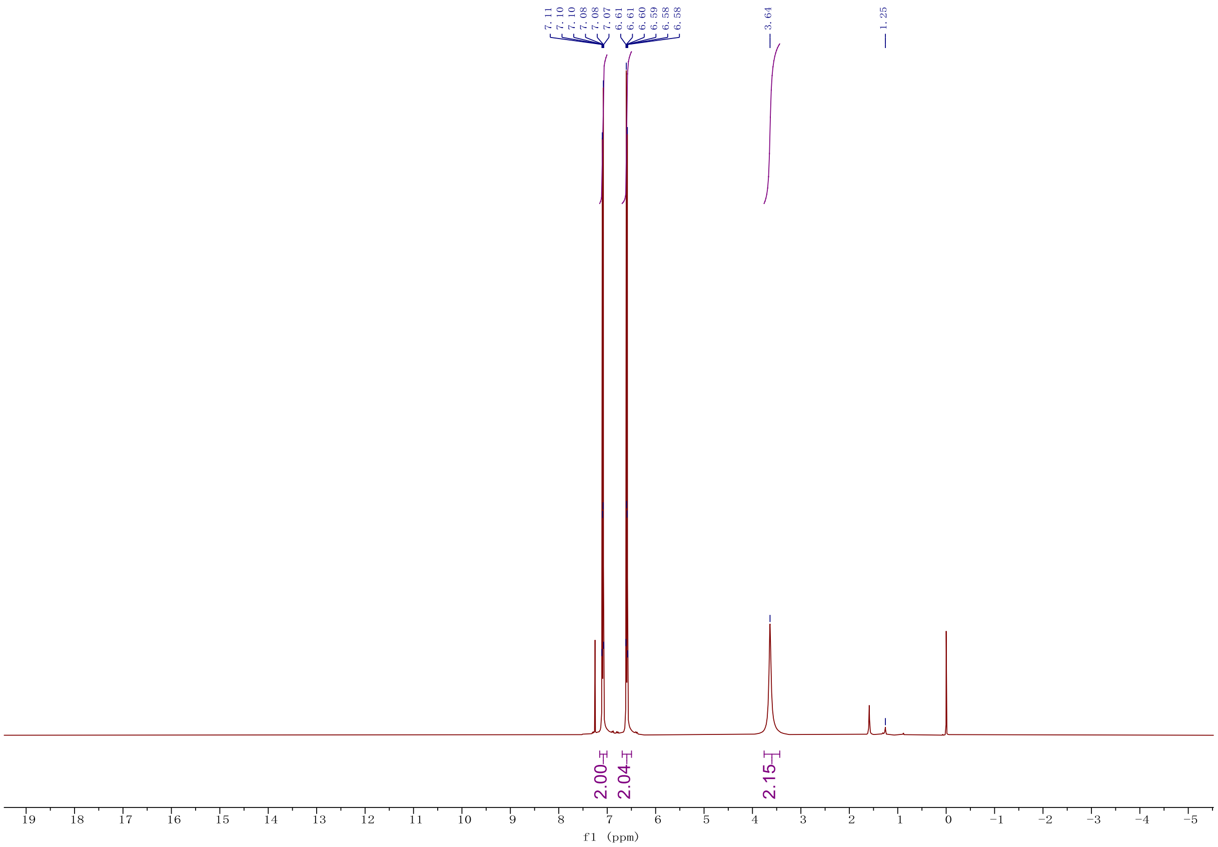

Figure S65 ^{1}H NMR for 4-chloroaniline

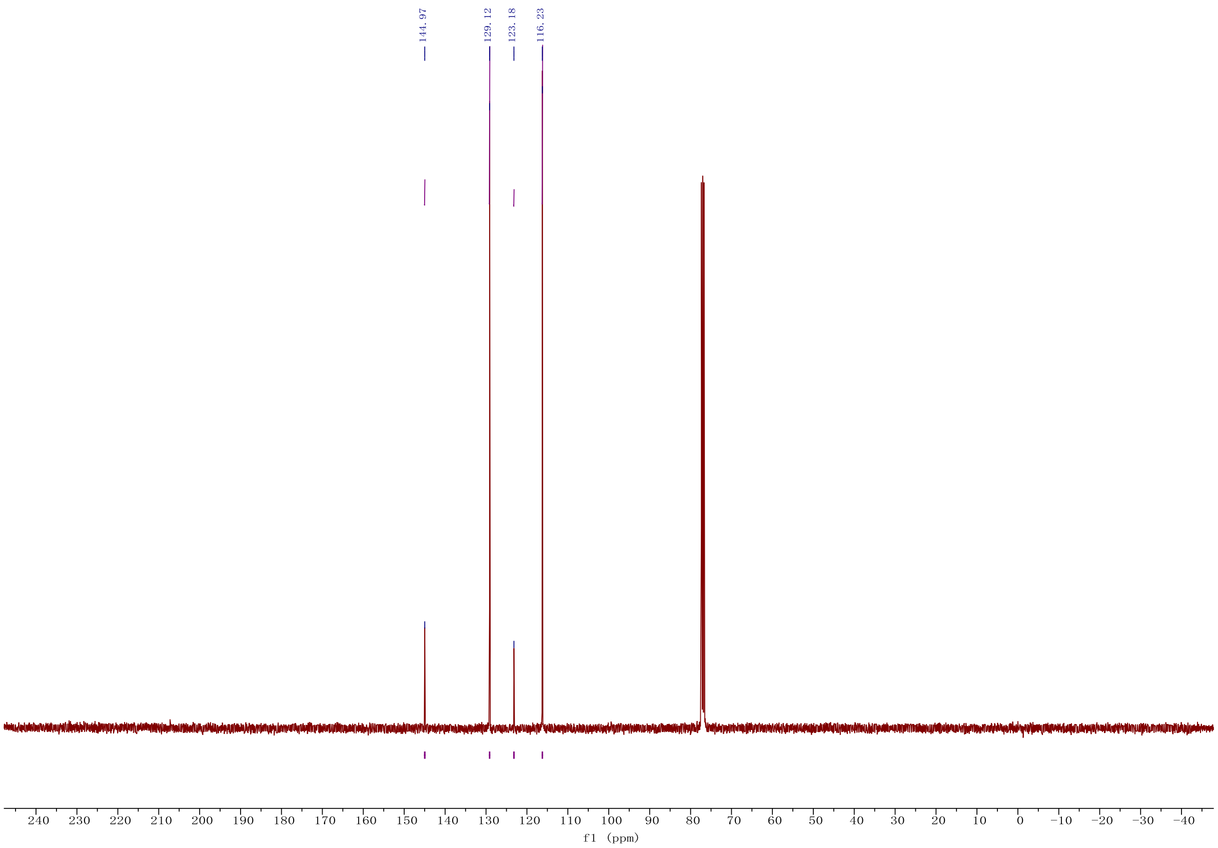

Figure S66 ^{13}C NMR for 4-chloroaniline

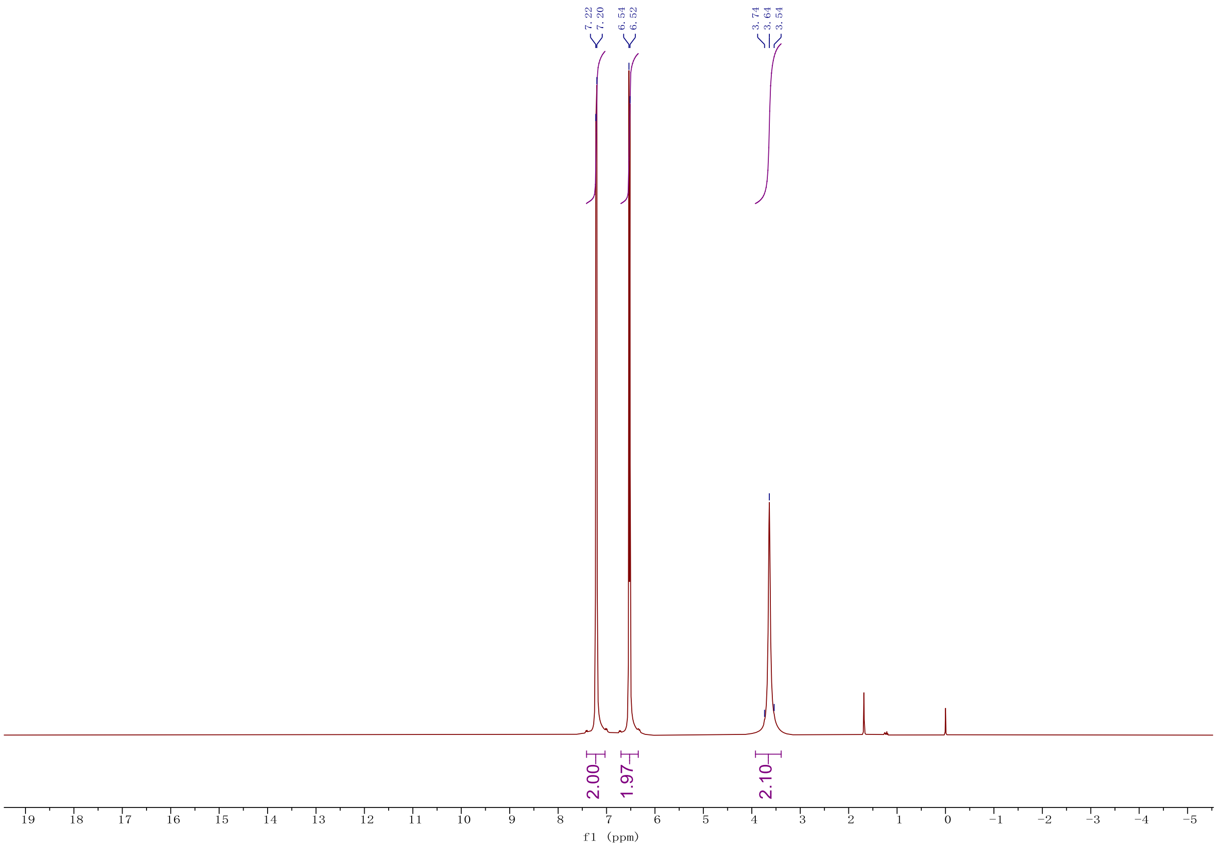

Figure S67 ^{1}H NMR for 4-bromoaniline

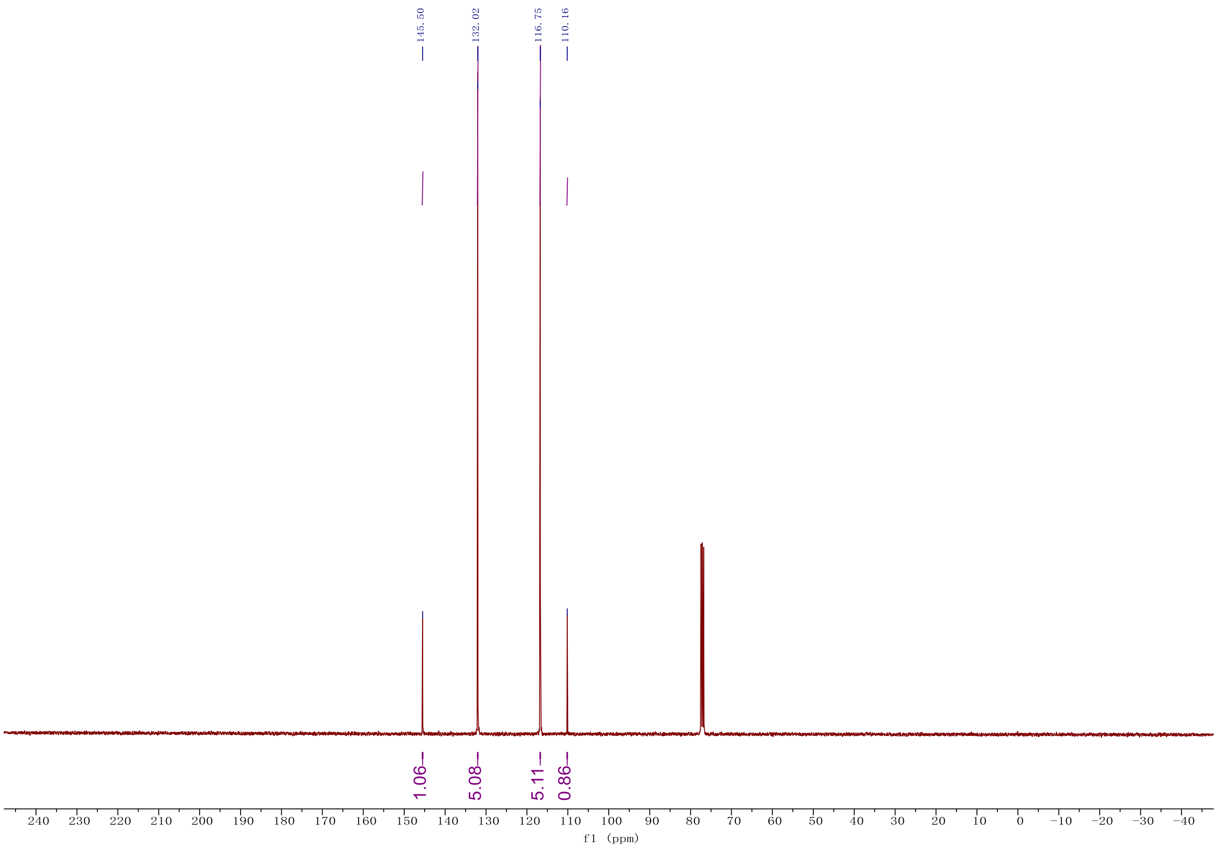

Figure S68 ^{13}C NMR for 4-bromoaniline

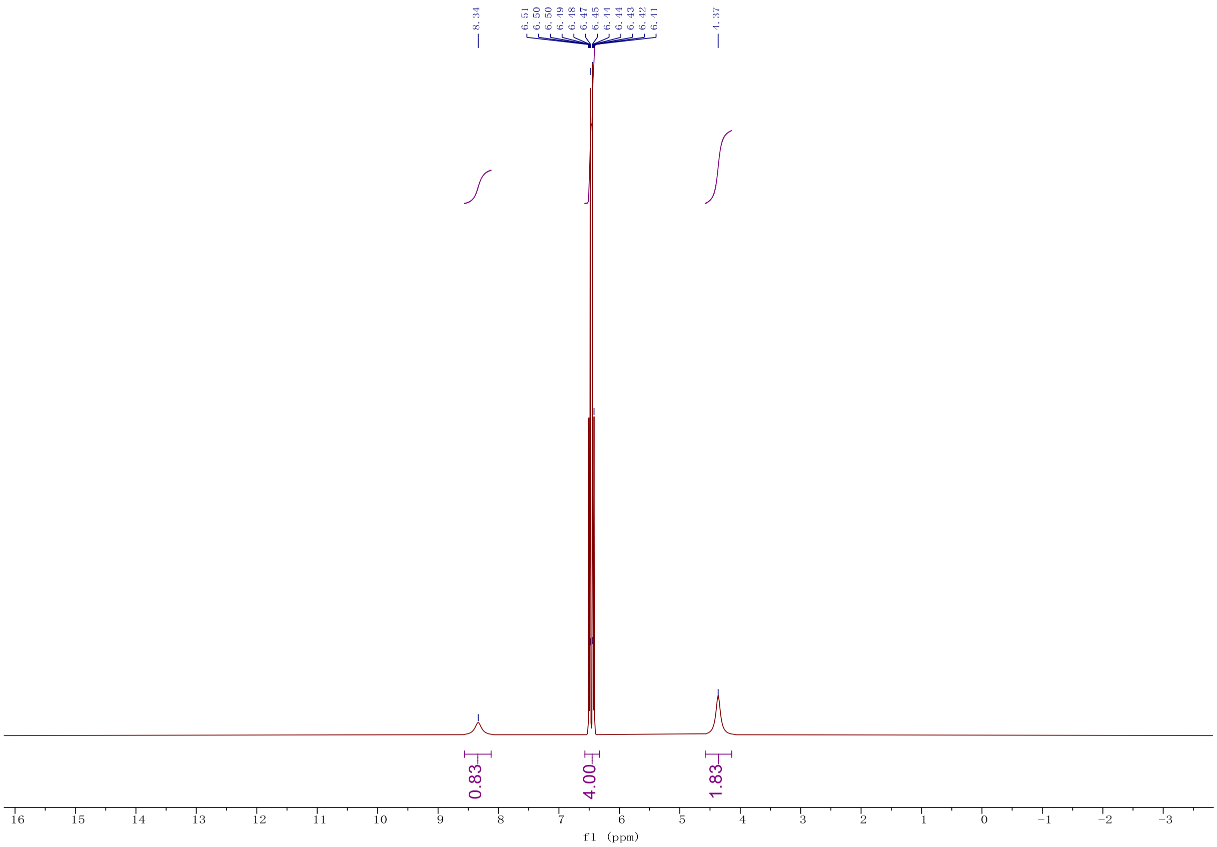

Figure S69 ^{1}H NMR for 4-aminophenol

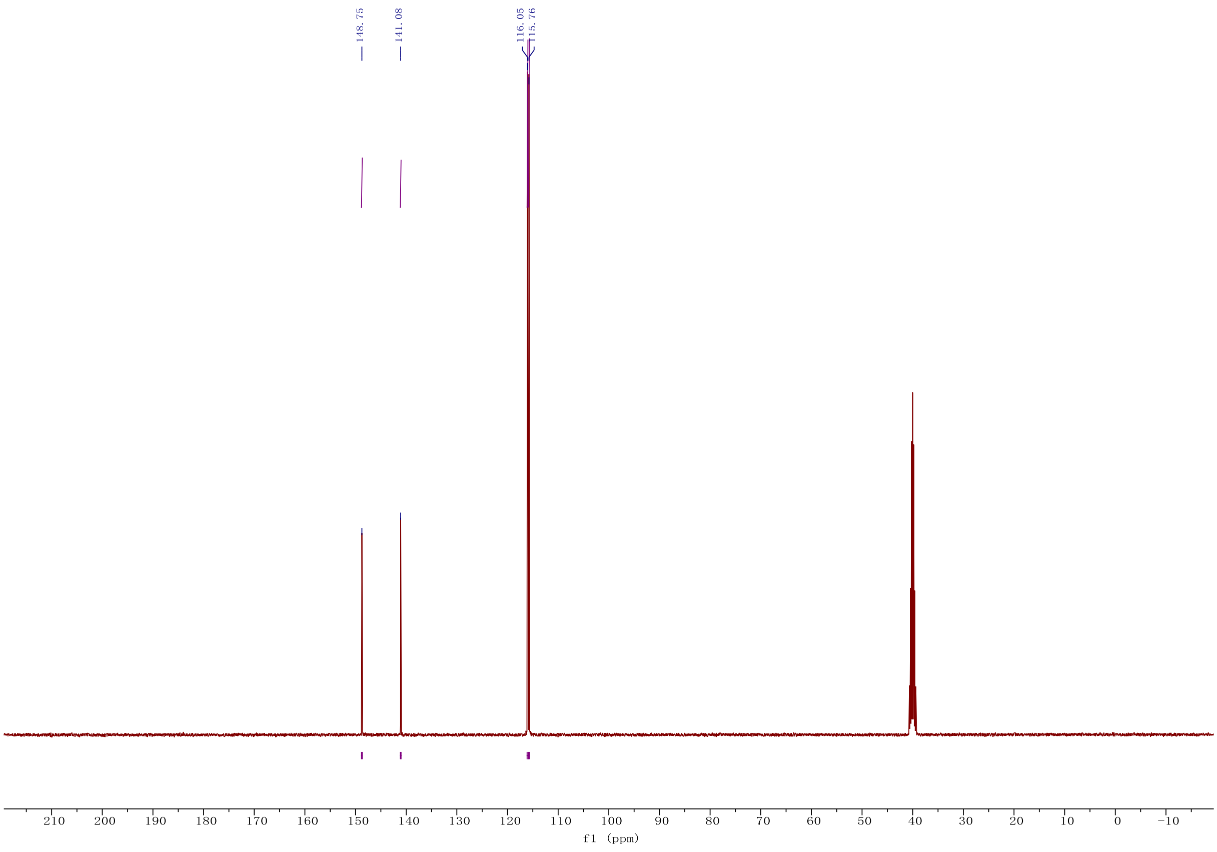

Figure S70 ^{13}C NMR for 4-aminophenol

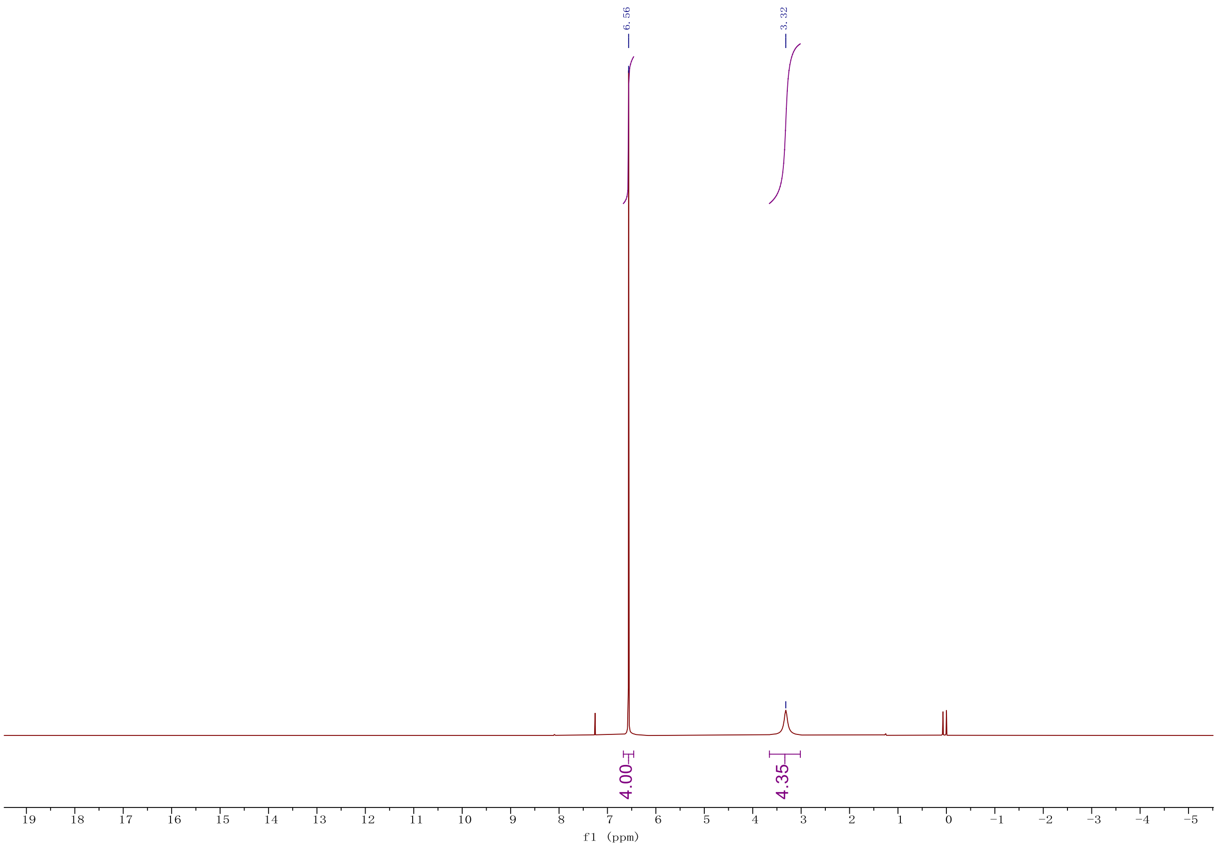

Figure S71 ^{1}H NMR for benzene-1,4-diamine

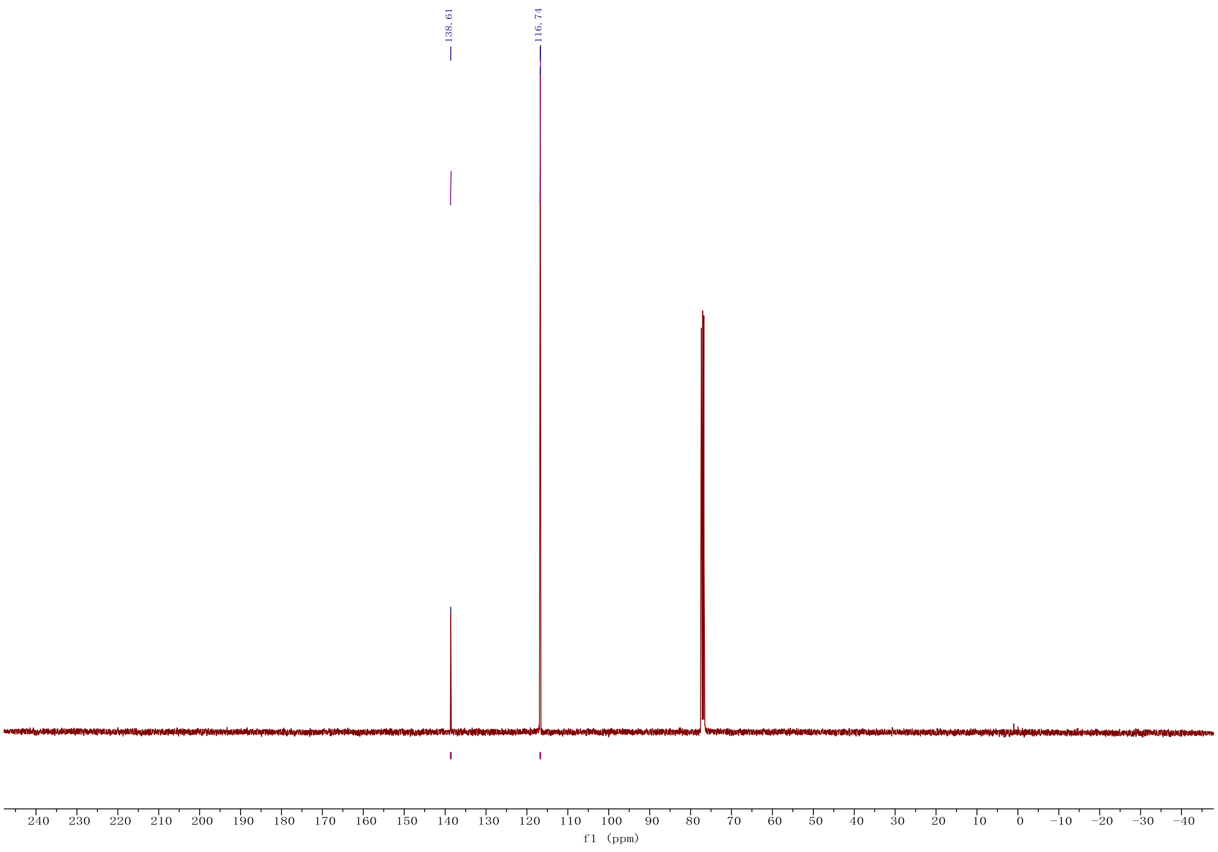

Figure S72 ^{13}C NMR for benzene-1,4-diamine
